# Supplementary material for: N4-acetylcytidine modification of ITGB5 mRNA mediated by NAT10 promotes perineural invasion in pancreatic ductal adenocarcinoma
Source: J Exp Clin Cancer Res. 2025 Mar 22;44:103. doi: 10.1186/s13046-025-03362-2 (PMC11929334; doi:10.1186/s13046-025-03362-2)
Supplement: Supplementary file 1 — Supplementary Material 1 [file 13046_2025_3362_MOESM1_ESM.pdf]

Supplementary uncropped WB images

Figure. 1I

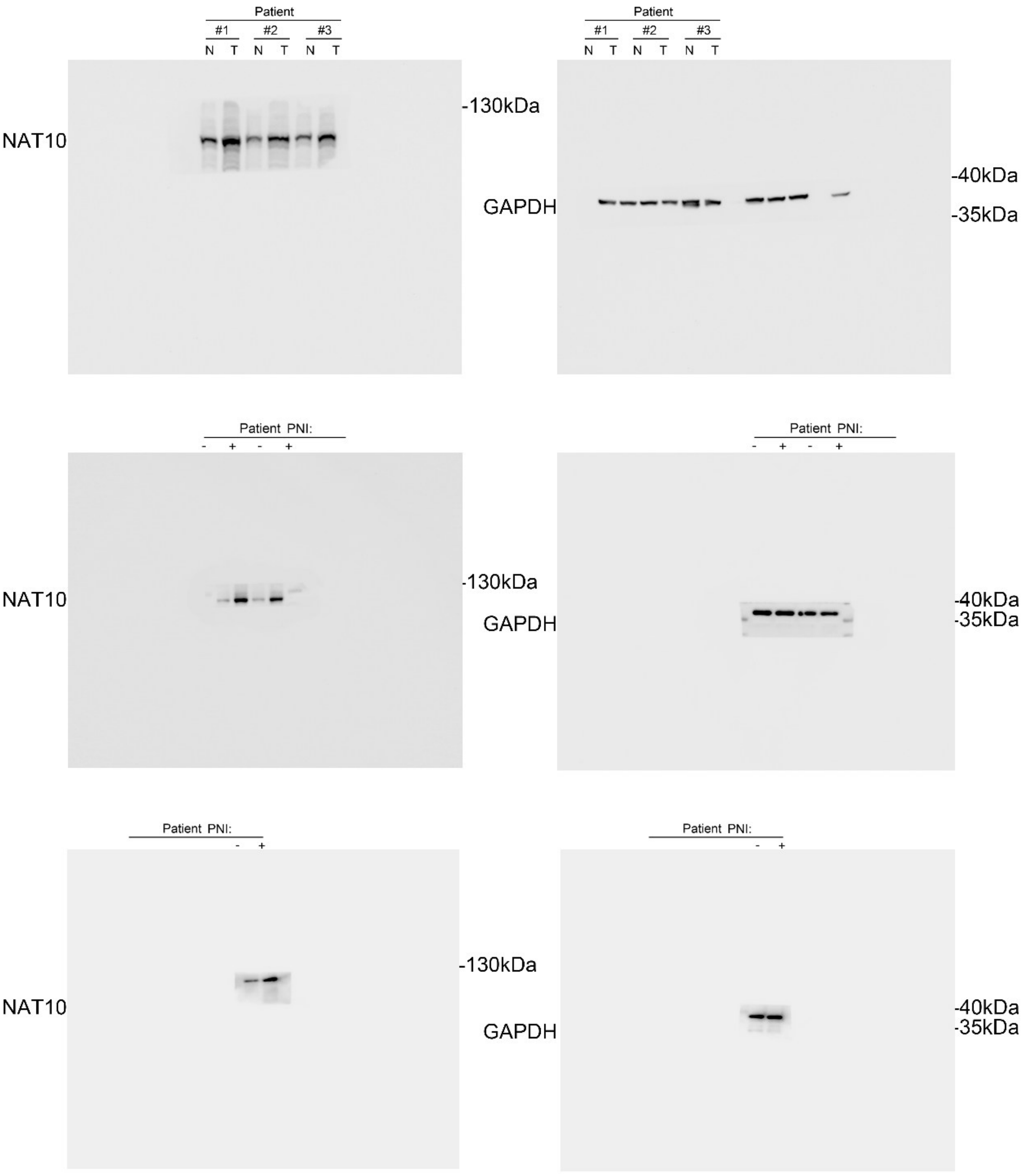

Figure. 2A

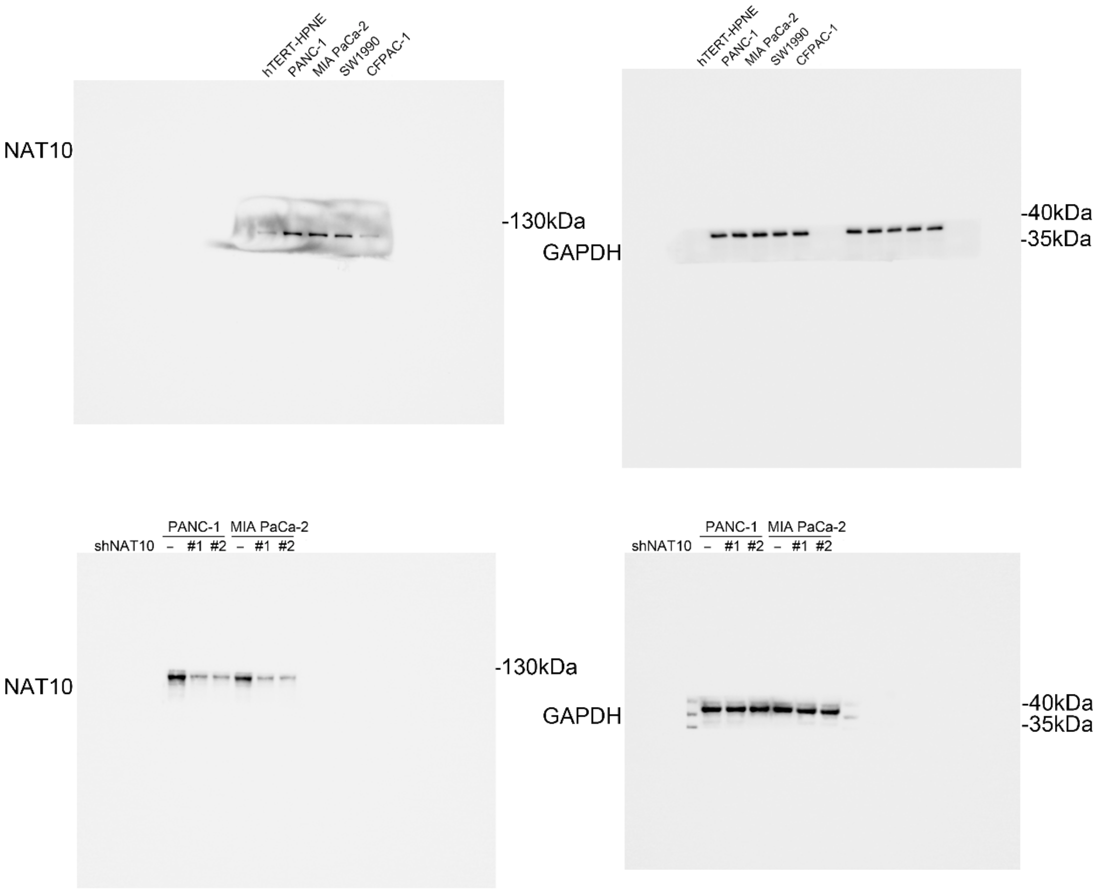

Figure. 3I

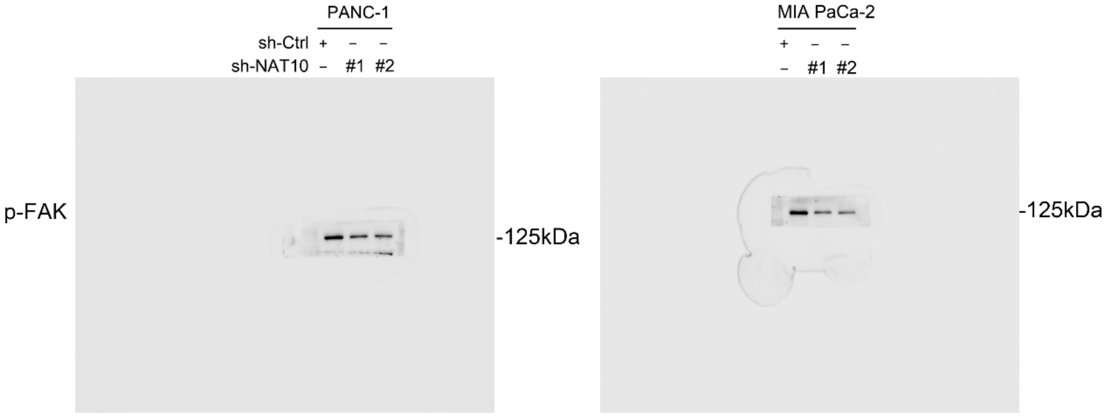

Figure. 3I

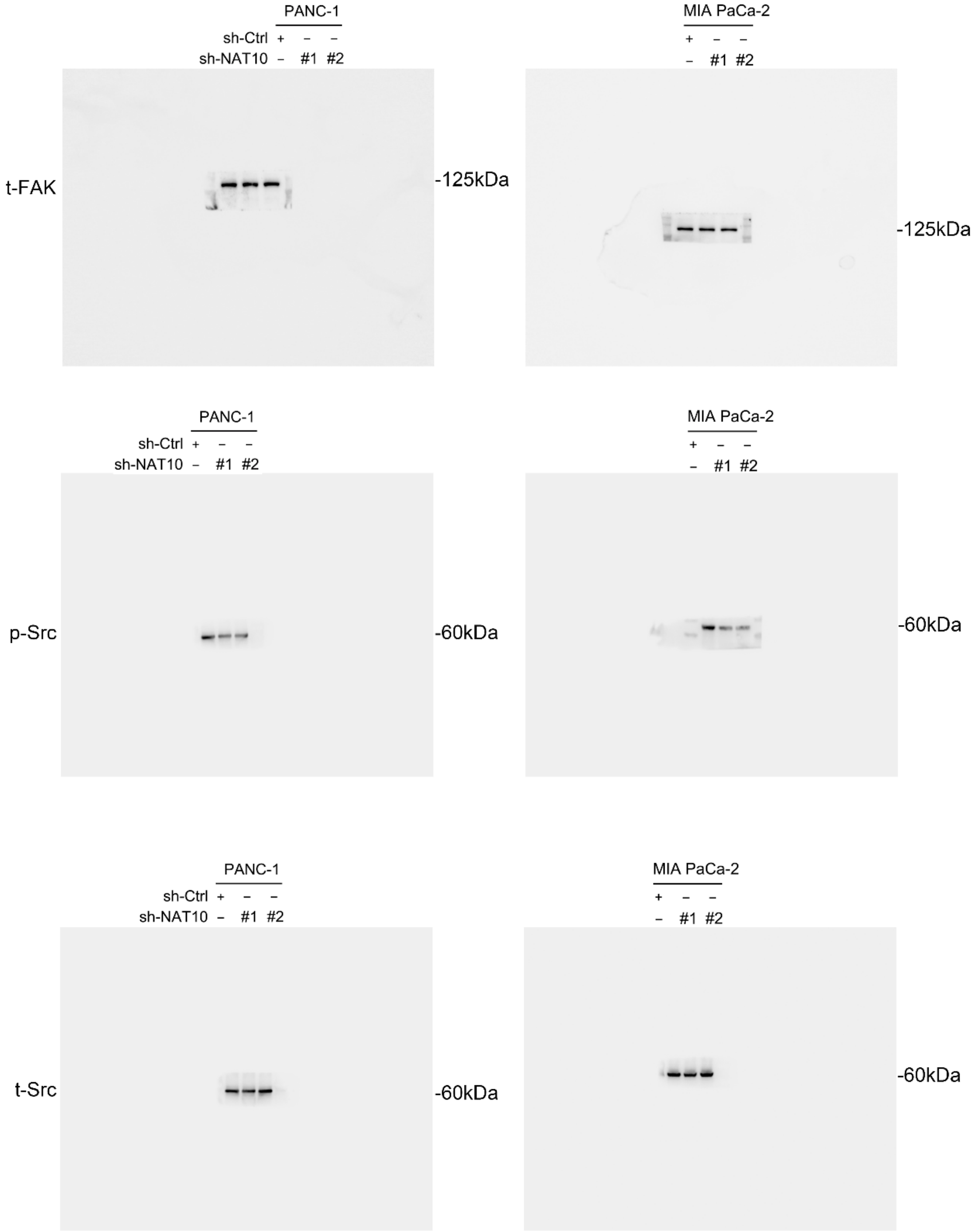
PANC-1
sh-Ctrl + – –
sh-NAT10 – #1 #2
MIA PaCa-2
+ – –
– #1 #2
t-FAK
-125kDa
-125kDa
PANC-1
sh-Ctrl + – –
sh-NAT10 – #1 #2
MIA PaCa-2
+ – –
– #1 #2
p-Src
-60kDa
-60kDa
PANC-1
sh-Ctrl + – –
sh-NAT10 – #1 #2
MIA PaCa-2
+ – –
– #1 #2
t-Src
-60kDa
-60kDa

Figure. 3I

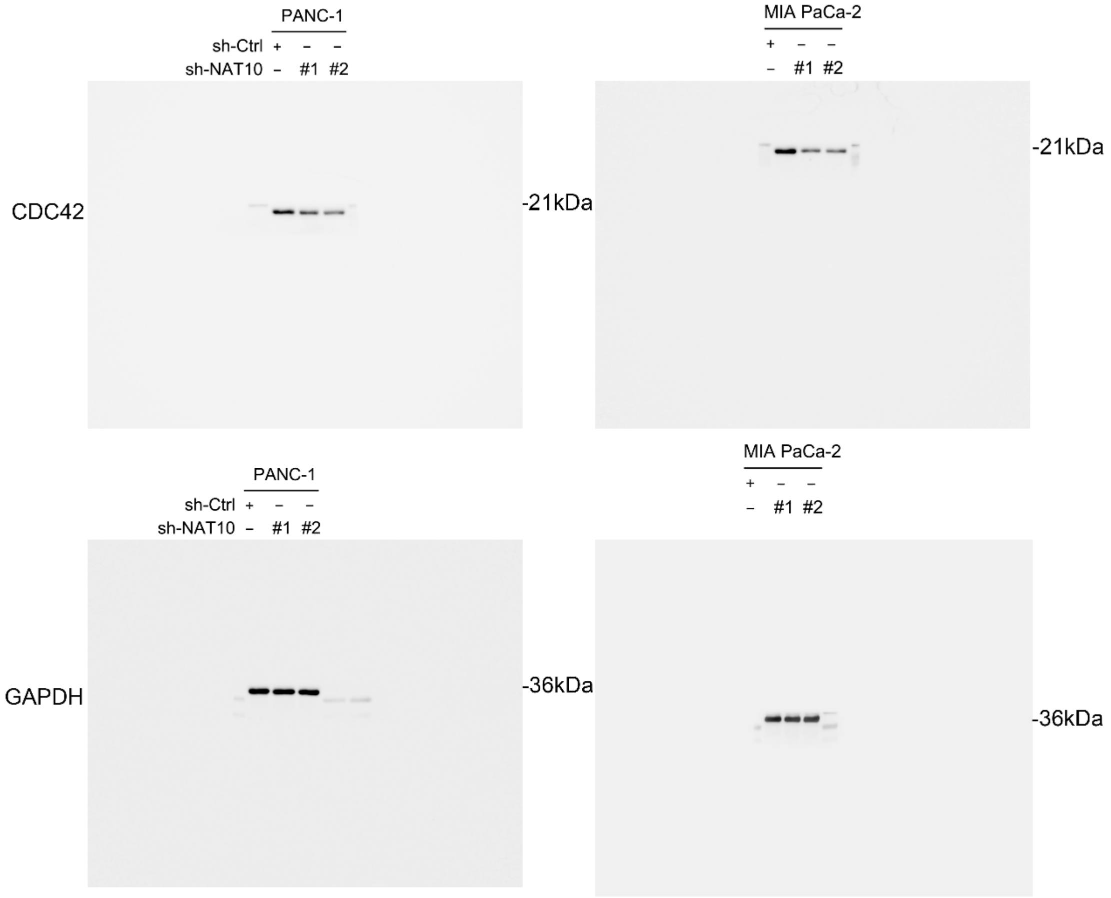

Figure. 3J

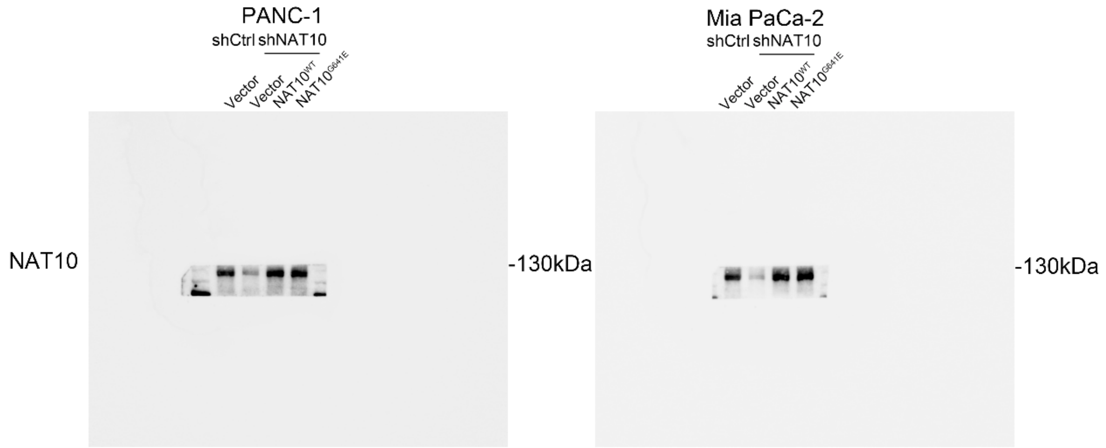

Figure. 3J

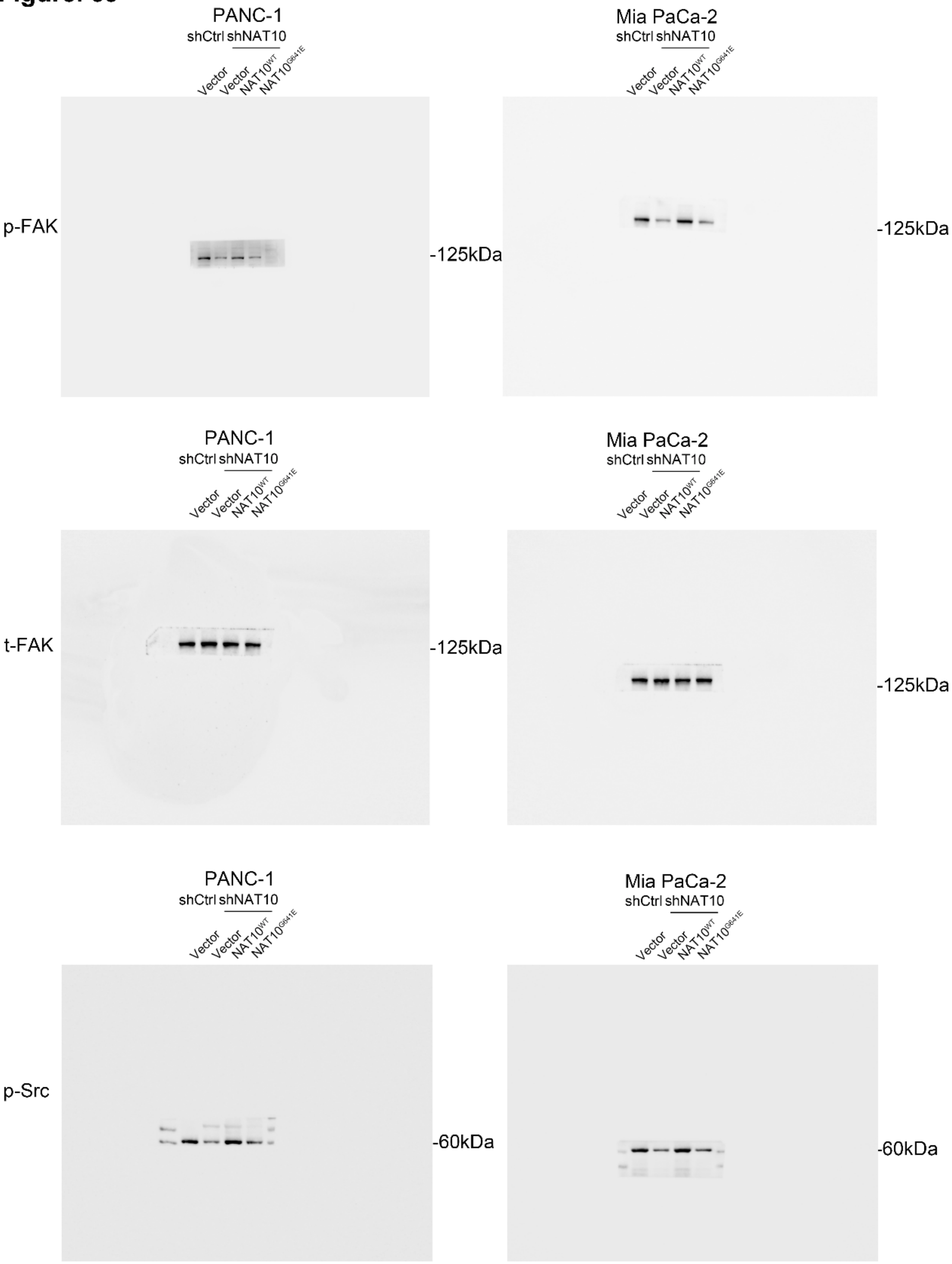
PANC-1
shCtrl shNAT10
Vector
Vector
NAT10^{WT}
NAT10^{G641E}
p-FAK
-125kDa
Mia PaCa-2
shCtrl shNAT10
Vector
Vector
NAT10^{WT}
NAT10^{G641E}
-125kDa
PANC-1
shCtrl shNAT10
t-FAK
-125kDa
Mia PaCa-2
shCtrl shNAT10
-125kDa
PANC-1
shCtrl shNAT10
p-Src
-60kDa
Mia PaCa-2
shCtrl shNAT10
-60kDa

Figure. 3J

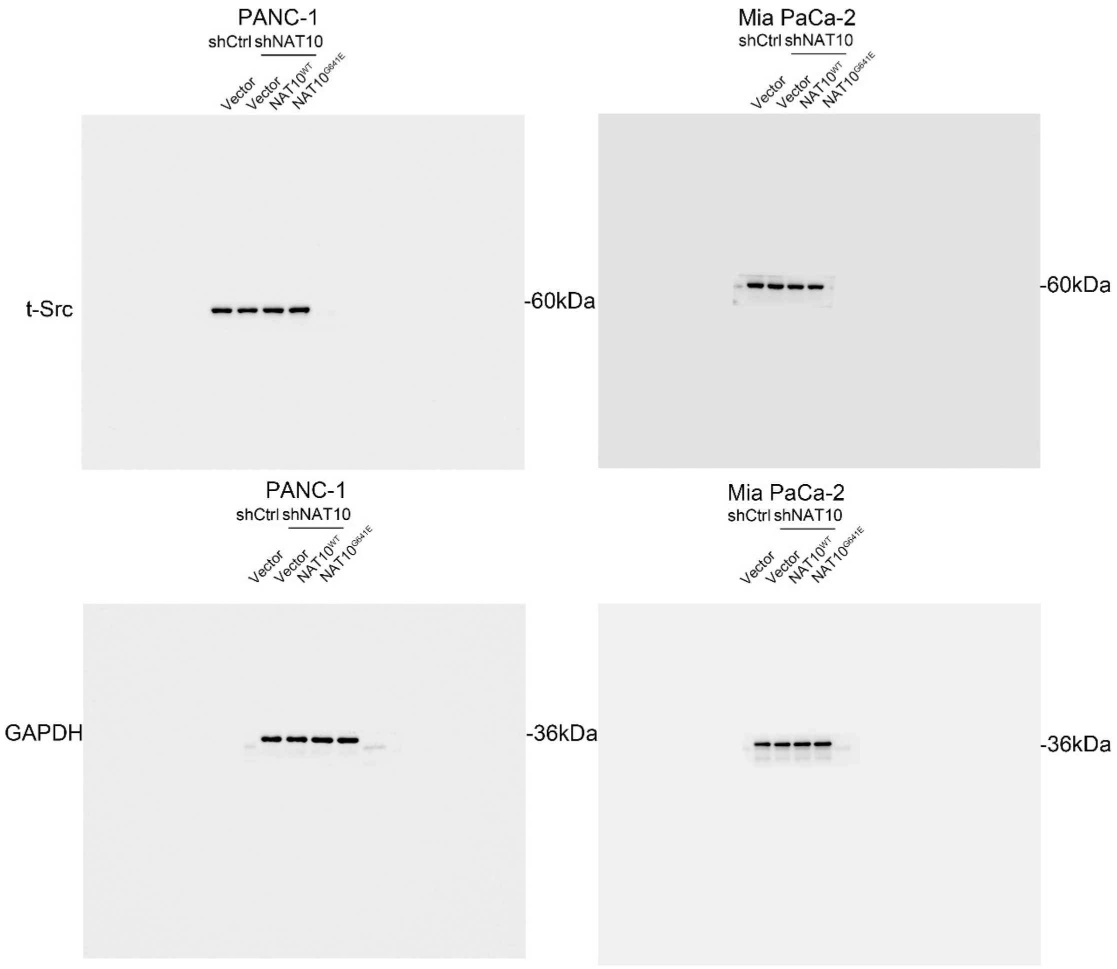

Figure. 4H

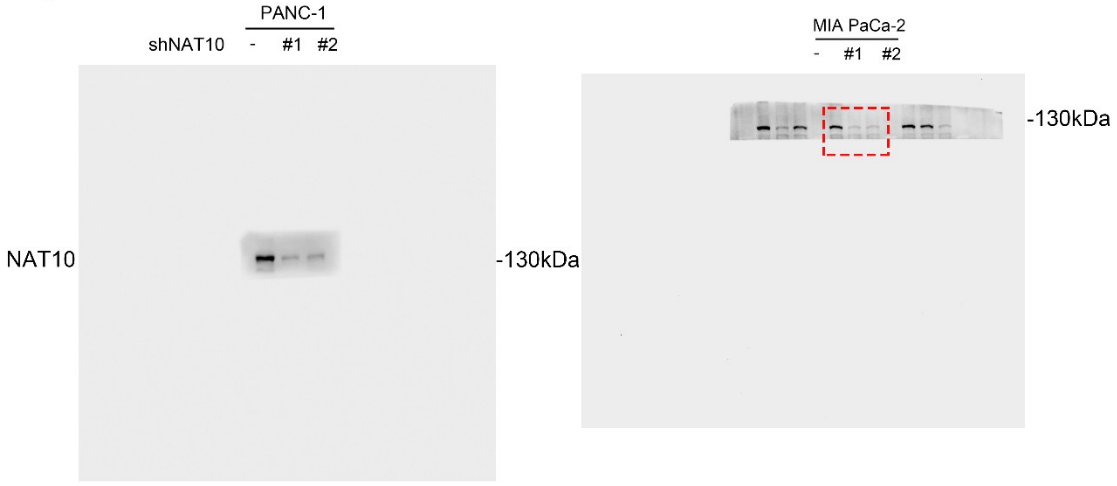

Figure. 4H

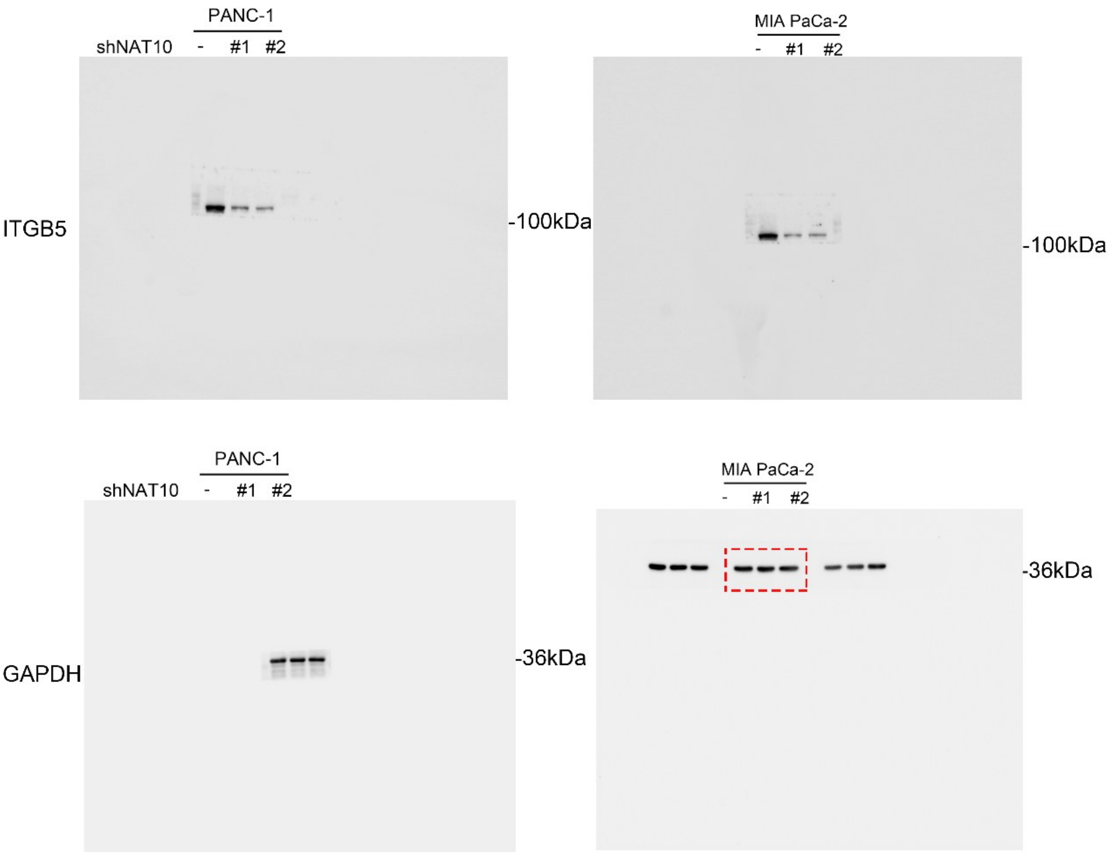

Figure. 4M

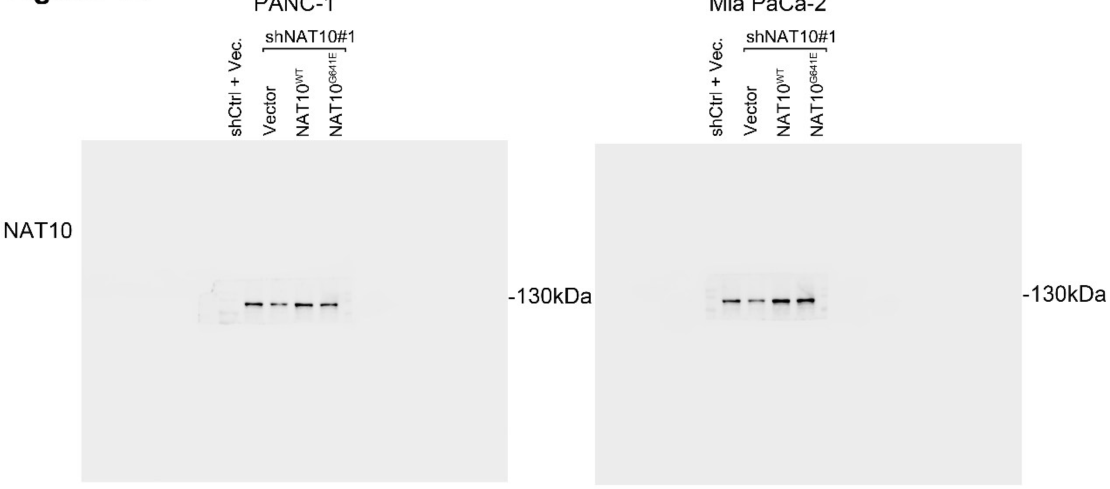

Figure. 4M

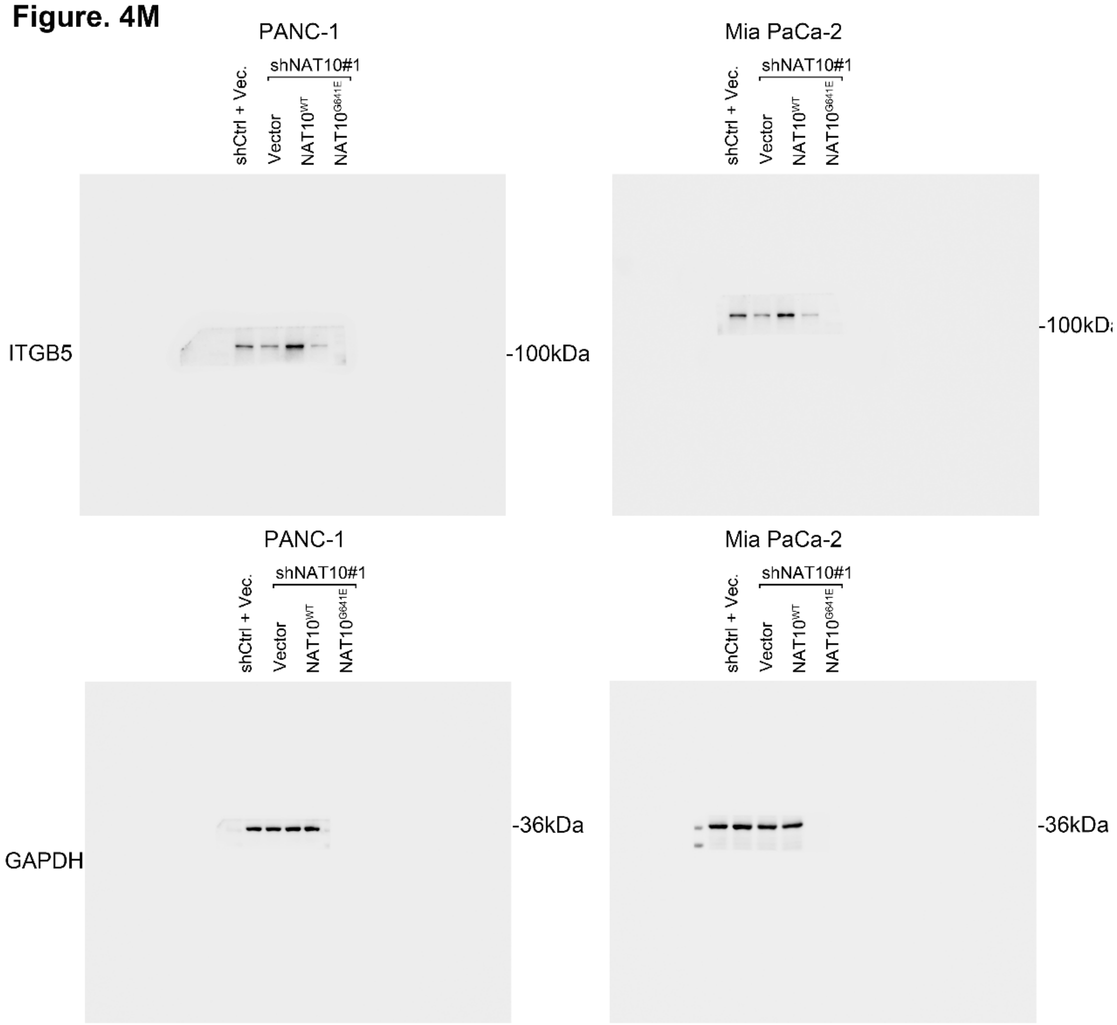

Figure. 5K

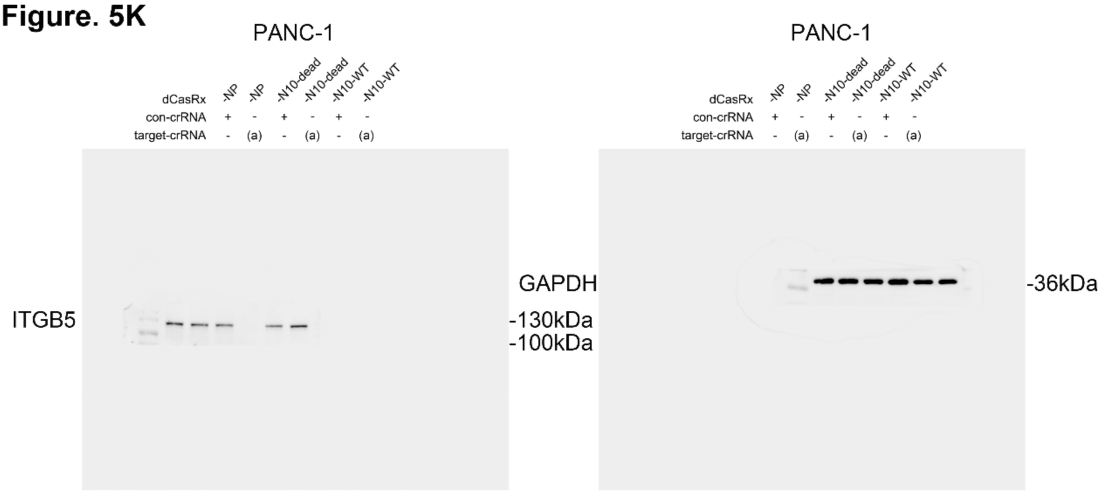

Figure. 6C

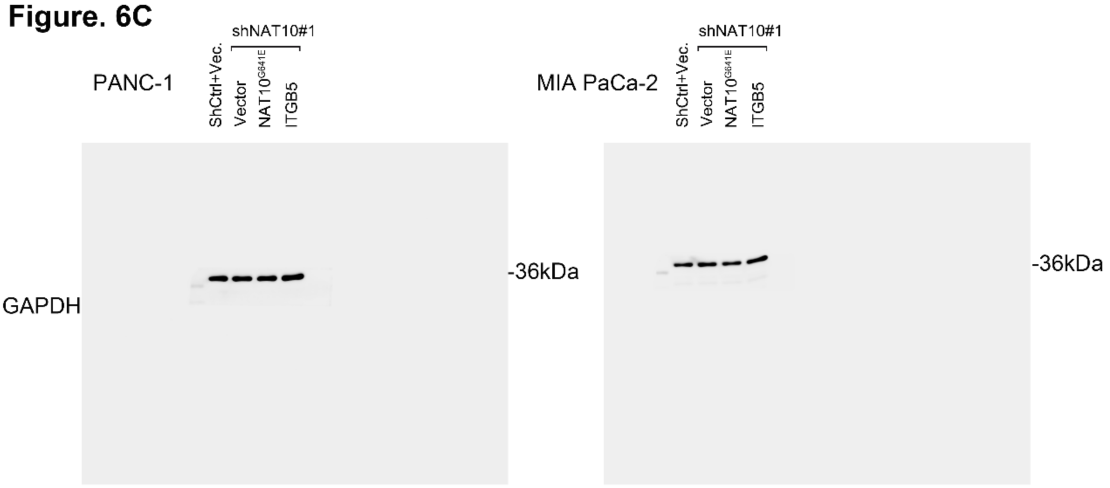

Figure. S2B

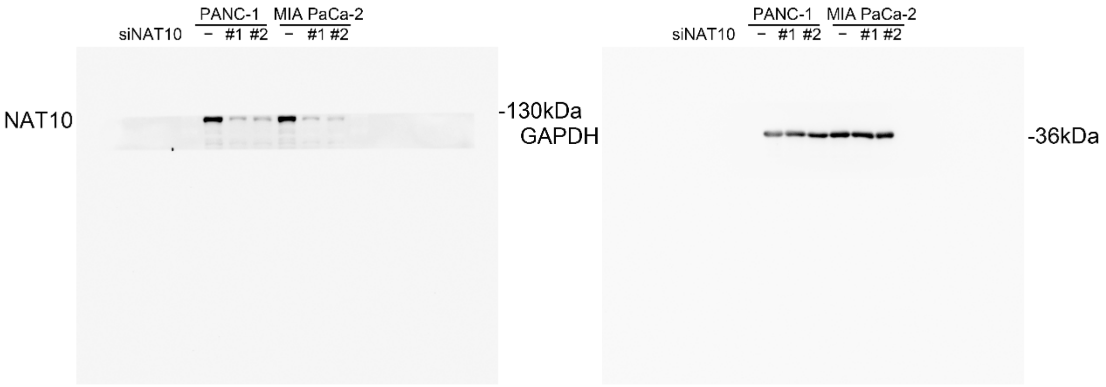

Figure. S3C

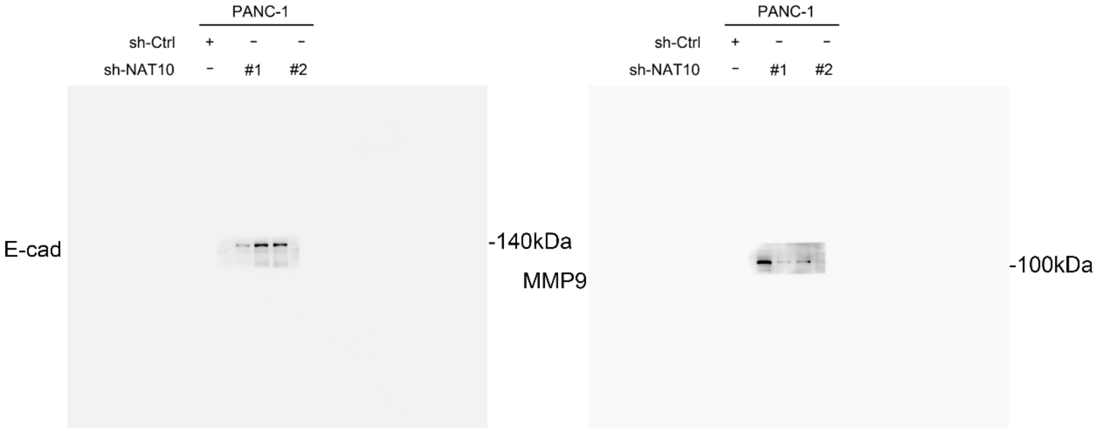

Figure. 5K

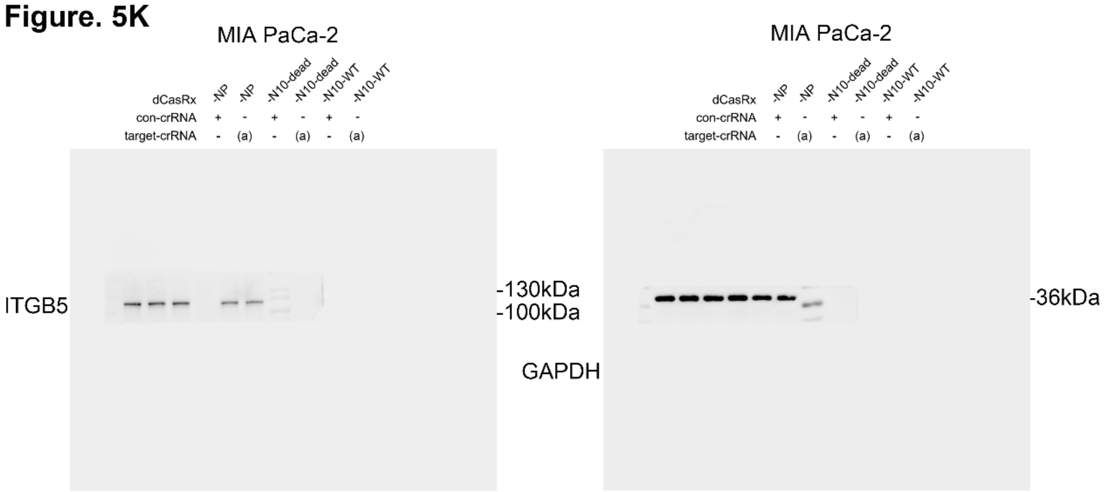

Figure. 6C

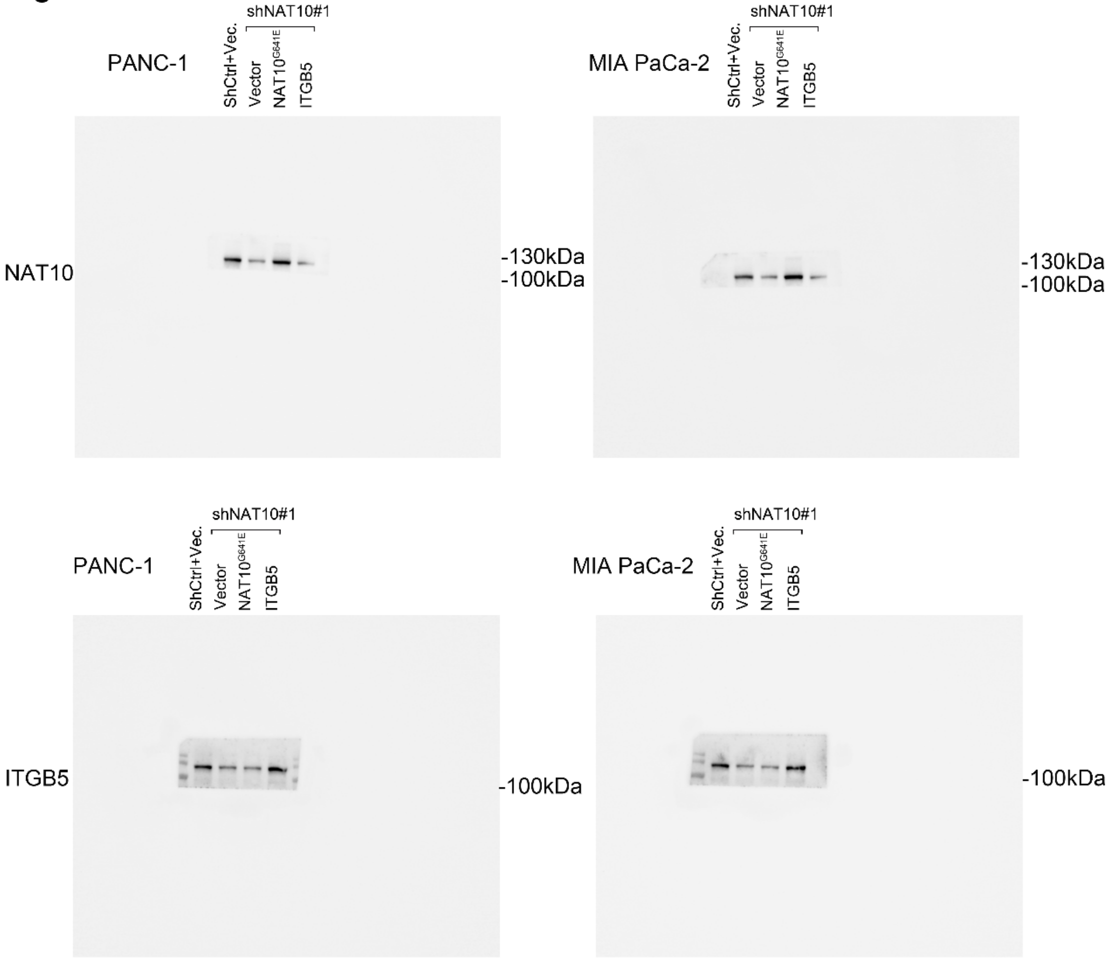

Figure. S6F

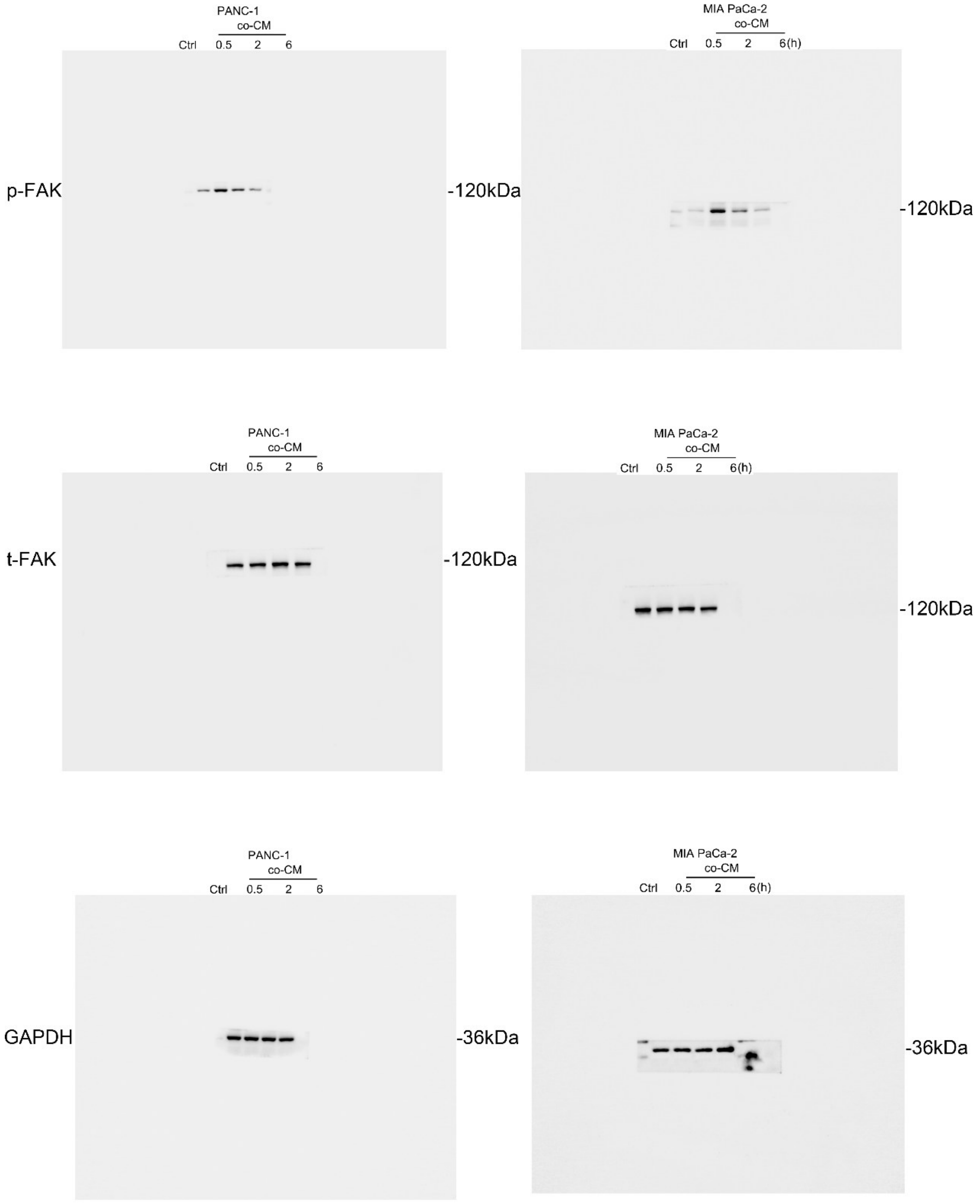
PANC-1
co-CM
Ctrl 0.5 2 6
p-FAK
-120kDa
MIA PaCa-2
co-CM
Ctrl 0.5 2 6(h)
-120kDa
PANC-1
co-CM
Ctrl 0.5 2 6
t-FAK
-120kDa
MIA PaCa-2
co-CM
Ctrl 0.5 2 6(h)
-120kDa
PANC-1
co-CM
Ctrl 0.5 2 6
GAPDH
-36kDa
MIA PaCa-2
co-CM
Ctrl 0.5 2 6(h)
-36kDa

Figure. S6G

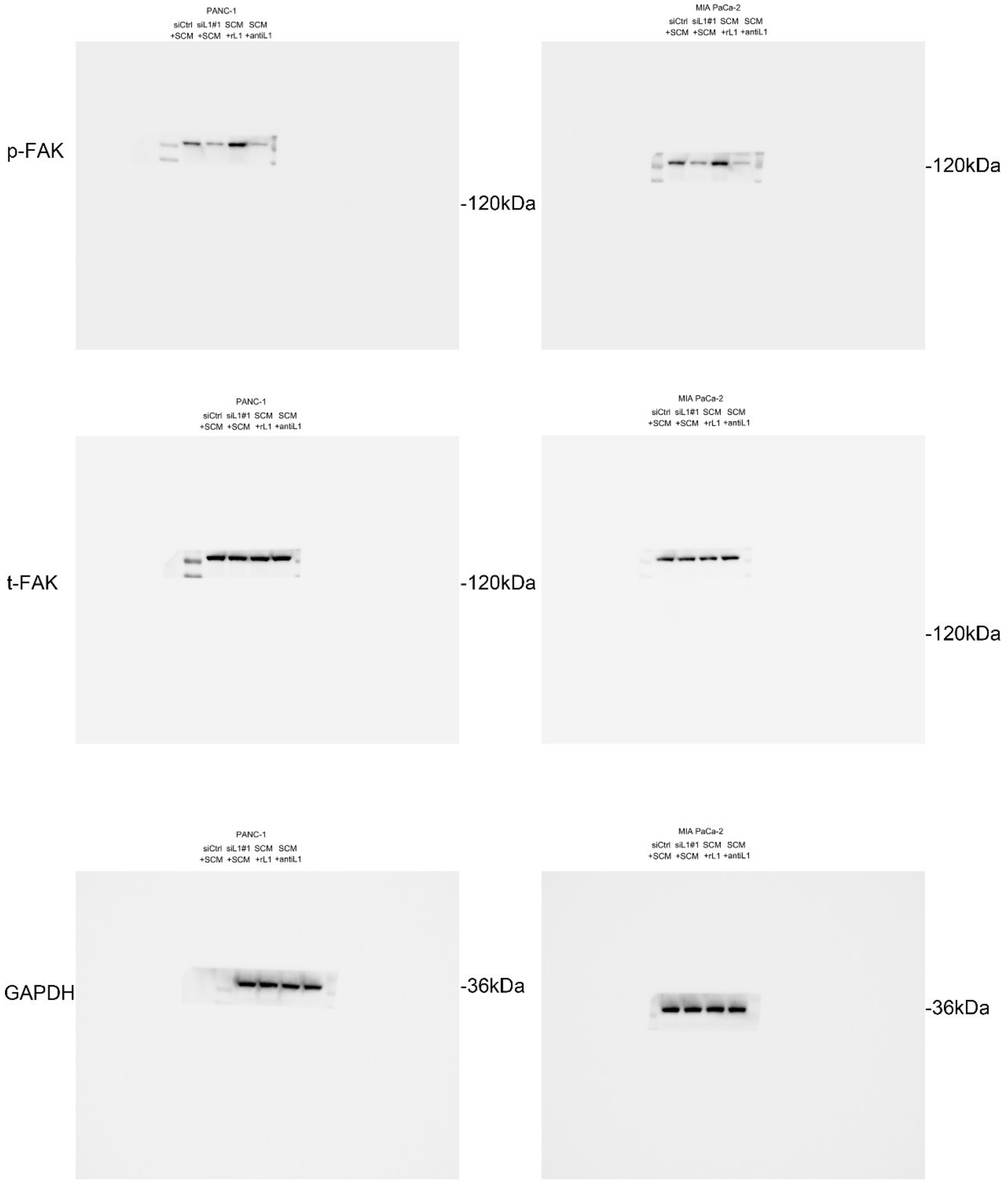
PANC-1
MIA PaCa-2
siCtrl +SCM
siL1#1 +SCM
SCM +rL1
SCM +antiL1
p-FAK
t-FAK
GAPDH
-120kDa
-36kDa

Figure. S7A

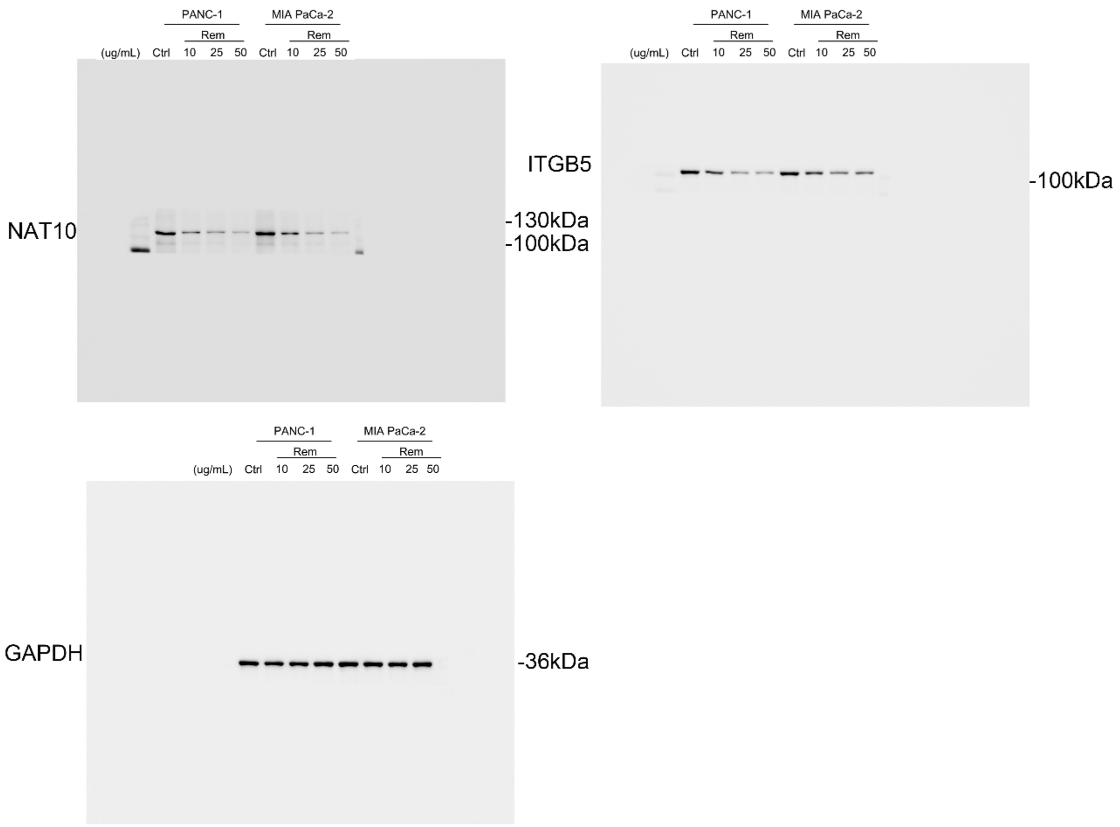
PANC-1
MIA PaCa-2
Rem
Rem
(ug/mL) Ctrl 10 25 50 Ctrl 10 25 50
NAT10
-130kDa
-100kDa
PANC-1
MIA PaCa-2
Rem
Rem
(ug/mL) Ctrl 10 25 50 Ctrl 10 25 50
ITGB5
-100kDa
PANC-1
MIA PaCa-2
Rem
Rem
(ug/mL) Ctrl 10 25 50 Ctrl 10 25 50
GAPDH
-36kDa
