# Supplementary material for: N4-acetylcytidine modification of ITGB5 mRNA mediated by NAT10 promotes perineural invasion in pancreatic ductal adenocarcinoma
Source: J Exp Clin Cancer Res. 2025 Mar 22;44:103. doi: 10.1186/s13046-025-03362-2 (PMC11929334; doi:10.1186/s13046-025-03362-2)
Supplement: Supplementary file 2 — Supplementary Material 2 [file 13046_2025_3362_MOESM2_ESM.pdf]

Supplementary Materials

N^4-acetylcytidine modification of *ITGB5* mRNA mediated by NAT10 promotes perineural invasion in pancreatic ductal adenocarcinoma

Huang et al.

MATERIALS AND METHODS

Cell Culture

Human pancreatic adenocarcinoma cell lines (PANC-1, MIA PaCa-2, CFPAC-1, and SW1990), human normal pancreatic duct cell line (hTERT-HPNE), human 293T cell lines, and human Schwann cell line sNF 96.2 were purchased from the ATCC (Rockville, MD, USA). PANC-1, MIA PaCa-2, 293T and sNF 96.2 were maintained in Dulbecco's modified Eagle's medium (DMEM). SW1990 was maintained in RPMI 1640 medium, and CFPAC-1 was in IMDM medium (Biological Industries). Primary KPC tumor cells were isolated from pancreatic cancer tissue of KPC mice, and cultured in DMEM until passage 5 or less. All these mediums were supplemented with 10% fetal bovine serum (Gibco) and 1% penicillin/streptomycin (Gibco) and cultured with 5% CO_2 in humidified air at 37°C. Mycoplasma screening is conducted every 1 to 2 months for all cell lines used and the cells are free of mycoplasma.

Plasmid construction and lentivirus infection

Short hairpin RNA (sh-RNA) lentivirus for human NAT10 was purchased from Gene-Pharma (Shanghai, China). The pCMV plasmid plasmids of human ITGB5 were synthesized by IGE biotechnology (Guangzhou, China). The plasmids of human NAT10, and NAT10 G641E mutant were synthesized by GeneBiogist biotechnology (Shanghai, China). For lentivirus conduction, psPAX2 and pMD2.G plasmid (IGE biotechnology) were transfected into HEK-293T using Lipofectamine 3000 (Invitrogen). For lentivirus infection, tumor cells were pre-seeded in 12-well plates at the day before infection. Polybrene was purchased from Gene-Pharma. All the sequences of oligonucleotides were listed in Table S5.

Cell transfection

Small interfering RNAs (siRNA) were transfected into cells with Lipofectamine® RNA Imax Reagent (Invitrogen, USA). Cells were harvested in 6-well plates when reached 60-80% confluence. RNA Imax Reagent and siRNA were diluted in Opti-MEM® Medium (Gibco, USA). Diluted siRNA was added to diluted RNA Imax Reagent in 1:1 ratio for 5 min at room temperature for incubation. Then the siRNA-lipid complex was added to cells. The results were analyzed after 48h. Plasmids were transfected into cells with Lipofectamine 3000 (Invitrogen, USA) as above mentioned. All siRNAs were purchased from Gene-Pharma (Shanghai, China) and sequences were listed in Table. S5.

Detection of mRNA modifications via LC-MS/MS

For sample preparation and extraction, 1 μg of mRNA was treated with S1 nuclease (180U/μL, Takara), Alkaline Phosphatase (30U/μL, Takara) and Phosphodiesterase I (0.002U/μL, Sigma-Aldrich), then incubated at 37°C until the mRNA was completely digested into nucleosides. Following digestion, the mixture was extracted with chloroform, and the aqueous layer was collected for LC-ESI-MS/MS analysis. Utilizing an ExionLC™ AD UPLC system coupled with an Applied Biosystems 6500 Triple Quadrupole mass spectrometer, samples were subjected to chromatographic separation on a Waters ACQUITY UPLC HSS T3 C18 column under a gradient program with water (2mM NH4HCO3) and methanol (2mM NH4HCO3) as solvents. Data acquisition was performed with Analyst software (V1.6.3), with specialized MRM

settings for mRNA modifications, further optimized for declustering potentials and collision energies. Quantification was conducted using Multiquant software (V3.0.3). This meticulous setup enabled comprehensive analysis of RNA modifications, which were carried out by MetWare Biotechnology Inc. (Wuhan, China) based on the AB Sciex QTRAP 6500 LC-MS/MS platform.

RNA isolation and quantitative real-time Polymerase Chain Reaction (qRT-PCR)

Fresh PDAC clinical samples were frozen with liquid nitrogen and thoroughly grinded into a fine powder. PDAC cells were washed with ice-cold Phosphate-Buffered Saline buffer (PBS) prior to collection. Total RNA was isolated using RNAiso Plus (TaKaRa, Japan) according to the manufacturer's protocol. mRNA was extracted from total RNA using Dynabeads mRNA Purification Kit (Thermo Fisher Scientific) as manufacturer's protocol. The total RNA was reverse transcribed to cDNA using PrimeScript RT Master Mix (TaKaRa, Japan). qRT-PCR was performed using SYBR® Green Premix *Pro Taq* HS qPCR Kit (AG, Accurate Biotechnology, China). The process was performed on LightCycler® 480 system (Roche, Switzerland). Thermal cycling reaction included two steps: pre-denaturation step at 95°C for 30sec and followed by 40 cycles of amplification step (denaturation at 95°C for 5sec, annealing and extension at 60°C for 30sec). Gene mRNA expression was normalized to housekeeping gene *GAPDH* and the relative expression were calculated using the $2^{\Delta\Delta}$CT method. All primers were listed in Table. S5.

RNA immunoprecipitation (RIP) using anti-ac4C antibody

We conducted RIP using anti-N4-acetylcytidine (ac4C) antibody and subsequently qRT-PCR to detect ac4C-modified mRNAs. The assay was performed using Imprint® RNA Immunoprecipitation Kit (Sigma-Aldrich), following the manufacturer's instructions. Briefly, ice-cold PBS washed cells (1×10^7) were lysed in complete RIP Lysis Buffer containing harsh lysis, protease inhibitor cocktail, and ribonuclease inhibitor. After washing with RIP wash buffer, protein A magnetic beads were pre-incubated with 5μg anti-N4-acetylcytidine (Abcam, ab252215) or IgG (Rabbit, I5006; Mouse, I5381) for 30min at room temperature. The RIP lysate was centrifuged, and 10% volume of the supernatant was for the input control. The remaining lysate was then incubated with antibody-bound magnetic beads overnight at 4°C with rotation. Each RIP reaction was filled with RNAiso Plus (TaKaRa, Japan) and1-bromo-3-chloropropane. Following centrifugation, the aqueous phase was mixed with linear acrylamide, 5 M ammonium acetate and 2-propanol for RNA precipitation. The acRIP results were analyzed with subsequently qRT-PCR and next-generation sequencing.

RNA-seq

Total RNA was extracted using TRIzol™ Reagent (Invitrogen) and detected by Bioptic Qseq100 Bio-Fragment Analyzer (Bioptic lnc.). DNase I (Invitrogen) treatment was adopted to remove DNA contamination. Additional phenol-chloroform isolation and ethanol precipitation treatments were performed to remove enzyme contamination. For RNA sequencing (RNA-seq), the VAHTS™ Stranded mRNA-Seq Library Prep Kit for Illumina® V2 (Vazyme) was used for the library preparation. About 1μg total RNA were used for mRNA capturing, fragmentation, the synthesis of cDNA, adapter ligation and library amplification. Illumina Novaseq 6000 system with paired-end 2×150 bp

read length was for sequencing.

acRIP-seq

For acetylated RNA immunoprecipitation sequencing (acRIP-seq), purified RNA was fragmented into ~200 nucleotide-long fragments. The Zymo RNA clean and concentrator-5 kit was used to purify fragmented total RNA (Zymo Research). The ac4C immunoprecipitation was performed by using Epi^{TM} ac4C immunoprecipitation kit (Epibiotek). Fragmented total RNA (Input) and immunoprecipitated RNA (IP) were subjected to library construction by using Epi^{TM} mini longRNA-seq kit (Epibiotek). Reverse transcription was performed using random primers, and the ribosome cDNA was removed after cDNA synthesis using probes specific to mammalian rRNA. Libraries quality was determined on Qseq100 Bio-Fragment Analyzer (Bioptic lnc.). Library was sequenced on Illumina Novaseq 6000 system with paired-end 2×150 bp read length.

ac4C-seq

The ac4C-seq experiment, along with high-throughput sequencing and data analysis, was carried out by Seqhealth Technology Co., LTD (Wuhan, China) with minor modifications as previously described[1,2]. 30 μg of total RNA was used to capture mRNA using the KAPA mRNA Capture Kit (KK8441). A small portion of the captured RNA was designated as "Con," while the remainder was treated with 100 mM NaBH4 at 55°C in the dark for 30 minutes. Reactions were adjusted to a volume of 200 μl using H2O, precipitated with 0.3M sodium acetate [pH 5.5], 15 mg/ml linear acrylamide (carrier), and 2.5X ethanol, briefly dried in a Speedvac, resuspended in 12 μl H2O, and quantified using Qubit4.0 with the Qubit™ RNA HS Assay Kit (Life Technologies, Q33224). Subsequently, the KC Digital Stranded mRNA Library Prep Kit for Illumina (DR085-02) was employed for library construction. This kit mitigates duplication bias in PCR and sequencing steps by employing unique molecular identifiers (UMIs) consisting of 8 random bases to label pre-amplified cDNA molecules. Library products corresponding to 200-500 base pairs were enriched, quantified, and finally sequenced on a Novaseq 6000 sequencer (Illumina) using the PE150 mode.

Data analysis

Raw sequencing data were initially filtered using fastp (version 0.23.1), discarding low-quality reads and trimming reads contaminated with adaptor sequences. UMI (version 1.0), the proprietary software developed by Seqhealth Co., Ltd, was utilized to eliminate duplicate reads. Sambamba (version 0.7.1) was employed for SAM/BAM format conversion and indexing. FeatureCounts (version 1.5.1) facilitated read distribution statistics. JACUSA (version 2.0.1) was applied to detect SNV sites and compile the corresponding pileup data. Rigel (version 1.0.0), another proprietary software, was used for statistical testing of detected SNV sites and identification of differential ac4C sites. Motif analysis was performed using Homer (version 4.10). Gene Ontology (GO) and Kyoto Encyclopedia of Genes and Genomes (KEGG) enrichment analyses for annotated genes were conducted using KOBAS (version 2.1.1), applying a corrected *P*-value cutoff of 0.05 for statistically significant enrichment.

dCasRx and sgRNA for editing ac4C

dCasRx-based ac4C editing tool were constructed by fusing acetyltransferase NAT10

wild-type or NAT10 with catalytic mutant G641E to SV40 dCasRx-2A-EGFP (GeneBiogist). The dCasRx-NAT10 (dead) conjugate contained a glycine to glutamate mutation at G641E to inhibit the N-acetyltransferase activity. Small-guide RNAs (sgRNAs) were designed at the cytosine of *ITGB5* mRNA based on our data of ac4C-seq. Sequence of sgRNAs were provided in Table. S5.

Immunohistochemistry (IHC)

The tissue sections were fixed in formalin and embedded in paraffin. After deparaffinized in xylene and rehydrating in graded alcohols, sections were soaked in 10 mmol/L citrate buffer (CB, pH=6.0, Boster Biological Technology, China) or Ethylene Diamine Tetraacetic Acid 9.0 (EDTA, pH 9.0, ZSGB bio, China), and heated in a pressure cooker for 10-15min. The tissues were pre-incubated with 3% hydrogen peroxide at room temperature for 10min and subsequently blocked with goat serum for 15min. The sections were incubated with primary antibodies for NAT10 (1:100), ITGB5 (1:300), or S100B (1:400) overnight at 4°C. After washing with phosphate-buffered saline (PBS), the samples were incubated with secondary antibodies (Servicebio, China) for 30min at room temperature and counterstained with hematoxylin. The proportion and staining intensity of NAT10 were as below: 0 score (negative), 1 score (0-10% positive cells; faintly positive), 2 score (10-25% positive cells; moderately positive), 3 score (25-50% positive cells; highly positive) and 4 score (more than 50% positive cells). All samples were examined and scored independently by two observers.

Immunofluorescence (IF)

For tumor cells, they were pre-seeded in a 35 mm laser confocal petri dish, and fixed in 4% paraformaldehyde for 20min at room temperature. Then cells were permeabilized in 0.5% Triton X-100 (ZSGB bio, China) for 20min and blocked with 1% BSA at room temperature for 1h. For frozen section, tissues were embedded in OCT compound. After incubating with primary antibody for NAT10 (1:100, Abcam), E-Cadherin (1:100, CST), p-FAK (1:300, CST) and p-Src (1:300, CST), S100B (1:400, Abcam), CK19 (1:500, Abcam) overnight at 4°C, samples were washed in tris buffered saline with 0.05% tween. The samples were incubated with corresponding secondary antibodies according to the primary antibody (donkey anti-rabbit/mouse Alexa Fluor 555, donkey anti-mouse/rabbit Alexa Fluor 488 [1:300]), and incubated for 1 h at room temperature protected from light. Cell nuclei were stained with DAPI for 10 min. The images were captured with Zeiss LAM900 focal Microscope (Carl Zeiss AG, Germany).

Actinomycin D assay

PANC-1, MIA PaCa-2, and SW1990 cells were pre-seeded in a 6-well plate and then treated with 5μg actinomycin D (Sigma) at indicated time points. After RNA extraction of cells in each well, the stability of ITGB5 mRNA was assessed by qRT-PCR assay.

Transwell assay

The Transwell assay was conducted using 8-μm 24-well Boyden chambers (Falcon, USA). The lower chambers were filled with 500μL DMEM medium containing 1% FBS. Treated cells were suspended into the upper chambers in 200μL serum-free culture medium, and then placed into the upper chambers. For invaded assays, the upper chambers were coated with Matrigel (BD biosciences, USA). PANC-1 and MIA PaCa-

2 cells were incubated for 14h, and 16h, respectively. The upper chambers were gently washed with PBS and fixed with 4% paraformaldehyde for 15min. After staining with 0.1% crystal violet, cells in the upper chambers were removed. Images were captured with Nikon Ni-U digital camera (Tokyo, Japan).

Western Blotting

The cells were washed with ice-cold PBS and lysed with RIPA buffer containing protease inhibitor and phosphatase inhibitor (Cwbio, China) for 30 min on ice. Ultrasonic disruption was used. The lysates were centrifuged at 12000×*g* for 20 min at 4°C, followed by calculation of protein concentration with bicinchoninic acid (BCA) Protein Assay Kit (Beyotime, China). The samples were boiled in SDS buffer (Invitrogen, USA) at 95°C for 10 min. For detection of ITGB5, samples were frozen at -80°C without boiling. 20-40μg of proteins were separated by SDS-PAGE gels and transferred to PVDF membranes. After blocking with 5% bovine serum albumin (BSA) for 1 h, the membranes were incubated with primary antibodies overnight at 4°C. After incubation of HRP-conjugated secondary antibodies, an enhanced chemiluminescence (ECL) kit (Thermo Scientific, USA) was used to detect bound antibodies. All the antibodies used in this study are listed in Table. S6.

Tumor cells treated with Schwann cell condition medium (SCM)

For collecting SCM, 1×10^6 sNF 96.2 cells were seeded in 10cm culture dishes. The cells were cultured in complete DMEM medium for 48 h to produce SCM. When the cell density reached 80% to 90%, the medium was replaced with serum-free RPMI 1640. After an additional 48 hours of incubation, SCM supernatant was collected and centrifuged at 2000 × g for 10 min to remove remaining cells or debris, then used immediately or store at −80 °C after aliquoting.

Prepare dorsal root ganglions (DRGs)

C57BL/6 mice aged two to three weeks, were anesthetized, fixed limbs, and disinfected the skin using 75% ethanol. After shaving the fur, a lateral incision was made along the midline of the skin. The skin was then retracted to expose the cervical, thoracic (T), and lumbar vertebrae. For coculture model, DRGs isolated from bilateral T8-10 vertebrae were used. Laminectomy was performed to remove the top of the spinal canal and exposed the spinal cord and DRGs. Then DRGs were promptly placed into 1640 medium on ice.

DRG-PDAC cells co-cultures

DRGs were embedded in 20μL Matrigel (BD biosciences, USA, 356230). To assess the PNI ability of NAT10-knockdown PDAC cells, PANC-1 or MIA PaCa-2 (2×10^5) were collected and resuspended in an equal volume of Matrigel, and then plated adjacent to the DRGs of each plate. Once Matrigel was solidified, DMEM medium with 10% FBS was added to each well and replaced every two days. On day 6, the recruitment of cancer cells was measured as previously reported[3]. The distance between PDAC and DRG was measured using ImageJ software (NIH, USA).

For treatments with small-molecular inhibitors, PDAC cells (5×10^4) were placed over each matrix-DRG assay after three days of DRG inoculation. Images were captured using an Olympus BX63 microscopy.

mRNAs dot blot assay

mRNAs were isolated from PDAC tissues and cell lines as above described. The diluted mRNA was heated at 95°C for 3min to disrupt secondary structures and immediately chilled on ice after denaturation. 2μL of mRNA was spotted onto a nitrocellulose membrane (Millipore, USA). Subsequently the membrane was transferred to UV Crosslinkers (XLE-1000, Spectronics, USA) equipped with 254nM bulb for crosslinking. The membrane was blocked with 5% BSA for 1 hour at room temperature and then incubated with anti-N4-acetylcytidine antibody (1:100, Abcam) overnight at 4°C. The membranes were washed with TBST and then incubated with second antibody (1:10000, Abcam) for 1 hour. The membrane was visualized using an ECL imaging system and subsequently stained with methylene blue as a loading control.

Treatment of Remodelin and defactinib

For DRG-Matrix model, DRGs were embedded into 20μg of Matrix gel. PANC-1 cells (3×10^4) were placed upon DRG-Matrix as previously reported[4]. Remodelin (50 μm, HY-16706, MedChemExpress, MCE, USA), Defactinib (20 μm, HY-12289, MCE, USA), Remodelin+Defactinb, or vehicle were added to culture medium after eight-day of inoculation. For small-molecule inhibitor treatment, 3-5 weeks old female C57bl/6 mice were injected pancreatic tumor cells (1×10^5) isolated from *Trp53*$^{em4(R172H)}$ *Kras*$^{em4(LSL-G12D)}$ Tg(Pdx1-cre)Smoc (KPC, Shanghai Model Organisms Center, Inc., China). After a week of tumor inoculation, Remodelin (60mg/kg, HY-16706, MCE, USA), Defactinib (30 mg/kg, HY-16706, MCE, USA), Remodelin+Defactinb, or vehicle was administered orally as indicated.

Polysome profiling assay

Cells for cytoplasmic ribosome analysis were treated with Cycloheximide (CHX, 50 μg/ml) prior to harvesting. The cell pellet was resuspended in 1 ml ice-cold buffer A1 (20 mM Tris-HCl pH 7.4, 130 mM KCl, 10 mM MgCl2, 50 μg/ml CHX) and centrifuged at 1200×g for 2 minutes at 4°C. The pellet was then lysed in 200 μl buffer A1 containing 0.5% Igepal CA-630, 0.5% sodium deoxycholate, 2.5 mM dithiothreitol (DTT), 0.2 mg/ml heparin, and 80 U/ml RiboLock (ThermoFisher, USA) for 10 minutes on ice. The lysate was cleared by centrifugation at 10,000×g for 10 minutes at 4°C. The supernatant was layered onto a 5–50% (v/v) sucrose gradient in 10 mM Tris-HCl pH 7.4, 60 mM KCl, 10 mM MgCl2, 1 mM DTT, 0.2 mg/ml heparin, and 0.01% Brij 35. Gradients were centrifuged at 160,000×g for 180 minutes at 4°C in a Beckman SW41Ti rotor. Linear gradient solution was prepared by Gradient Master Model 108 (BioComp, Canada). Fractions were collected using a Piston Gradient Fractionator (BioComp, Canada) and absorbance at 260 nm was measured.

Statistical analysis

All quantitative data were described as the mean ± SD. The statistical analysis was performed using SPSS Statistics 25.0 (IBM Corp., USA) and Prism 9.0.0 (GraphPad Software, USA). The correlation between NAT10 and clinicopathological characteristics was analyzed using the Chi-squared test (χ^2 test). Independent prognostic factors were identified using the Cox proportional hazards model. Survival times were calculated using the Kaplan-Meier method and compared by the log-rank test. Two-tailed Student's t-test was used for comparisons between two groups, while one-way analysis of variance (ANOVA) was used for comparisons among multiple

groups. Bonferroni correction was applied to adjust p-values for multiple comparisons. The statistical methods are indicated in the Fig legends. A *p*-value of <0.05 was considered statistically significant in all tests.

References:

1. Thalalla GS, Sas-Chen A, Schwartz S, Meier JL. Quantitative nucleotide resolution profiling of RNA cytidine acetylation by ac4C-seq. Nat Protoc. 2021; 16: 2286-307.
2. Arango D, Sturgill D, Yang R, Kanai T, Bauer P, Roy J, et al. Direct epitranscriptomic regulation of mammalian translation initiation through N4-acetylcytidine. Mol Cell. 2022; 82: 2797-814.
3. Deborde S, Omelchenko T, Lyubchik A, Zhou Y, He S, McNamara WF, et al. Schwann cells induce cancer cell dispersion and invasion. J Clin Invest. 2016; 126: 1538-54.
4. Huyett P, Gilbert M, Liu L, Ferris RL, Kim S. A Model for Perineural Invasion in Head and Neck Squamous Cell Carcinoma. J Vis Exp. 2017.

Supplementary Figures

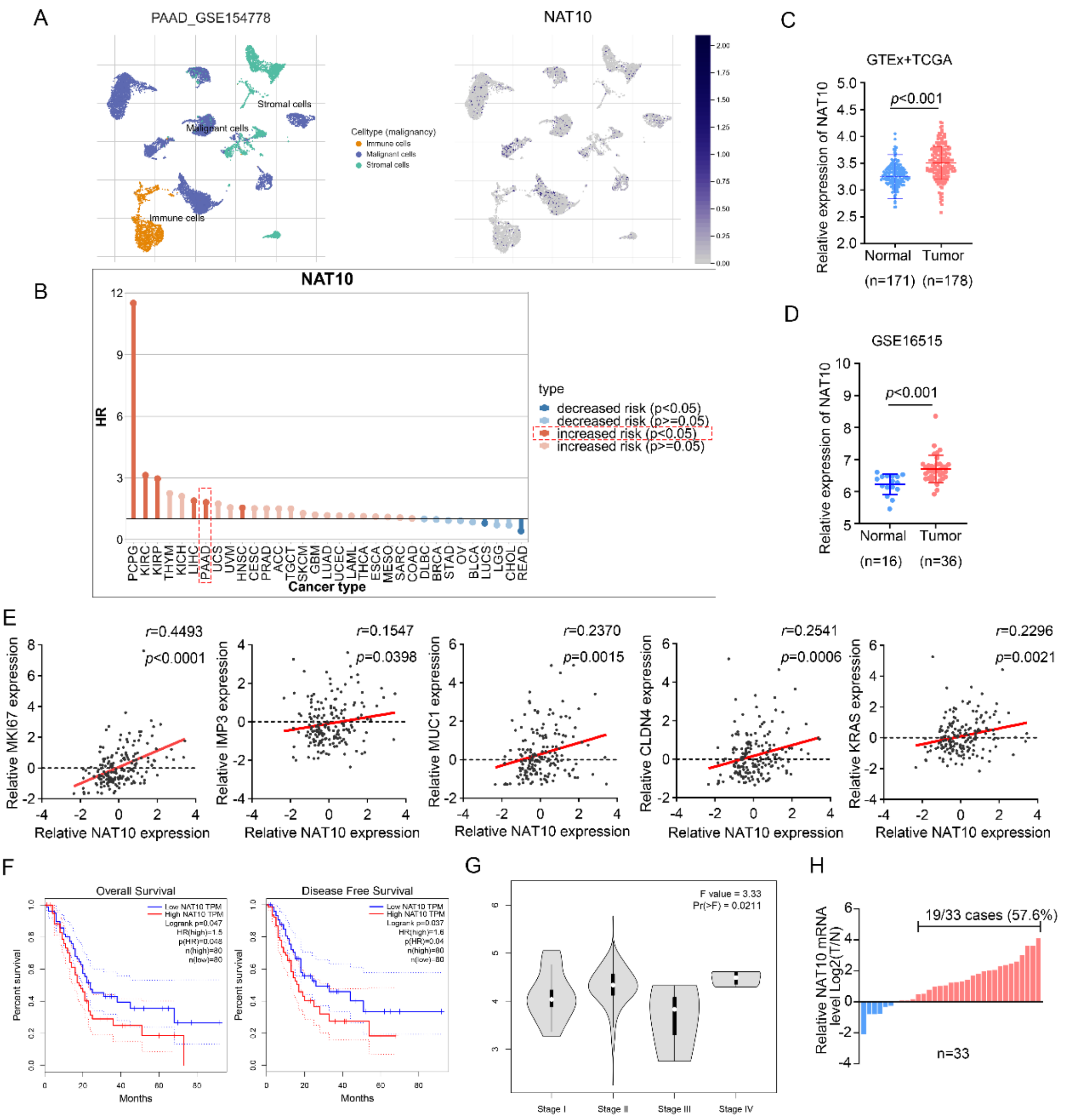

Supplementary Fig. 1 Related to Fig. 1

(A) UMAP analysis of 10 pancreatic primary tumor and 6 metastatic biopsies (GSE154778) showed NAT10 mainly expressed on malignant cells. **(B)** Analysis using TISCH showed the NAT10's hazard ratio (HR) and p-value across 33 cancer types in TCGA. **(C-D)** NAT10 expression levels in Pancreatic adenocarcinoma (PAAD) and normal tissue respectively from TCGA and GTEx database (C), and from GEO database GSE16515 (D). **(E)** Correlation analysis between NAT10 and biomarker of pancreatic cancer. MKI67, Marker of Proliferation Ki-67; IMP3, Insulin-like Growth Factor 2 mRNA Binding Protein 3; MUC1, Mucin 1; CLDN4, Claudin 4 and KRAS, Kirsten Rat Sarcoma Viral Oncogene. **(F-G)** Overall survival, disease-free survival analyses (F) and stage plot (G) correlated to NAT10 expression were conducted using GEPIA on PAAD patients from TCGA database. **(H)** Waterfall plot of the relative NAT10 mRNA levels from 33 paired samples of PDAC and normal tissue measured using qRT-PCR. (C), and (D), Mann-Whitney U-test.

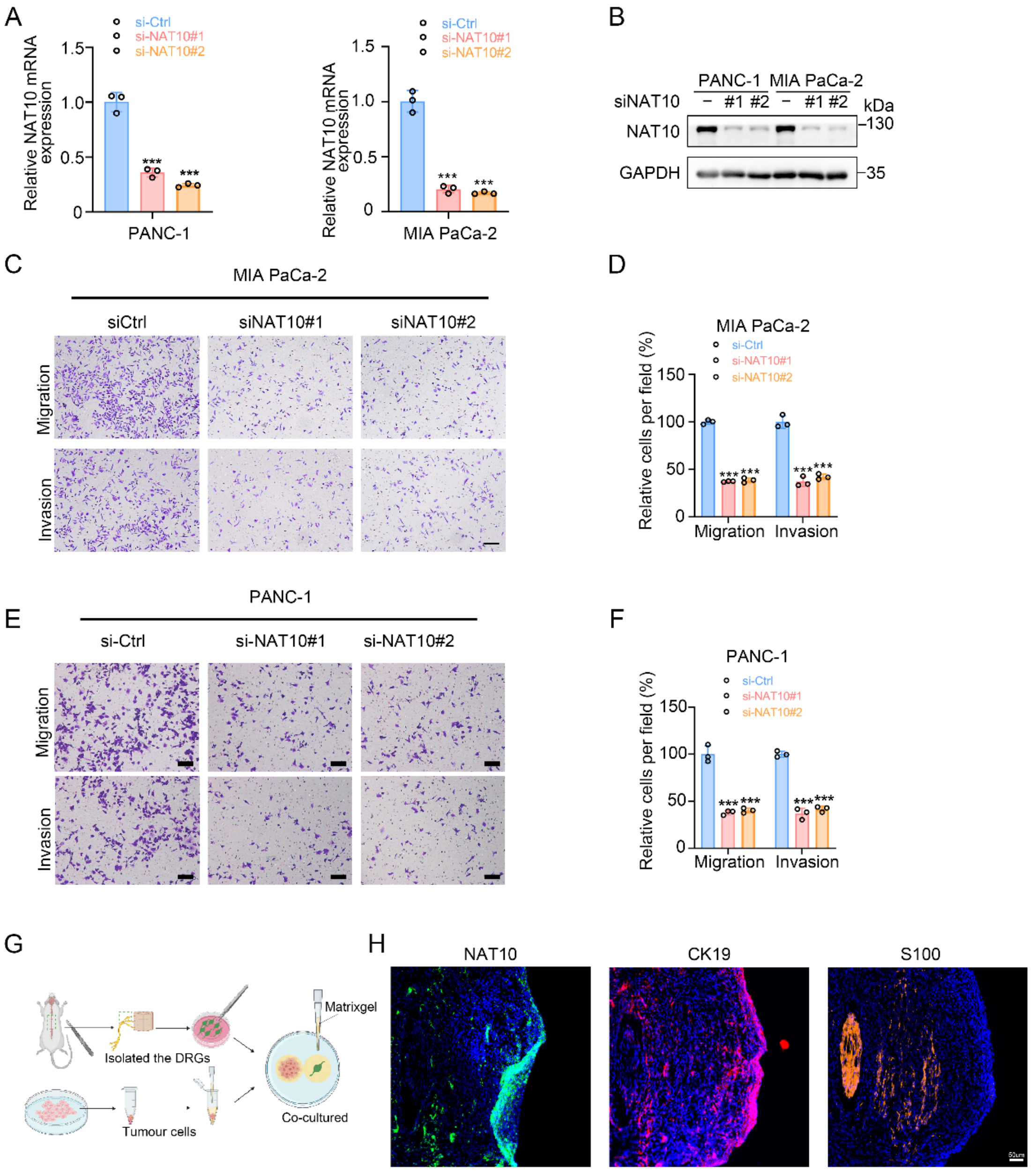

Supplementary Fig. 2 Related to Fig. 2

(A) Relative mRNA expression of NAT10 in NAT10 KD (via siRNA) and control PANC-1 (left) or MIA PaCa-2 (right) cells. **(B)** Representative immunoblotting image of NAT10 in NAT10 KD (via siRNA) and control PANC-1 or MIA PaCa-2 cells. **(C-F)** Representative cell migration and invasion image, and quantification of MIA PaCa-2 (C-D) or PANC-1 (E-F) cells expressing NAT10 siRNA. Scale bar: 200 μm **(G)** Scheme of DRG-PDAC cells coculture system. **(H)** Immunofluorescent image of NAT10, tumor marker CK19, and Schwann cells marker S100β in mice sciatic nerve. Scale bar: 50 μm. **p*<0.05, ***p*<0.01, ****p*<0.001. (A), (D), and (F), Student's t test.

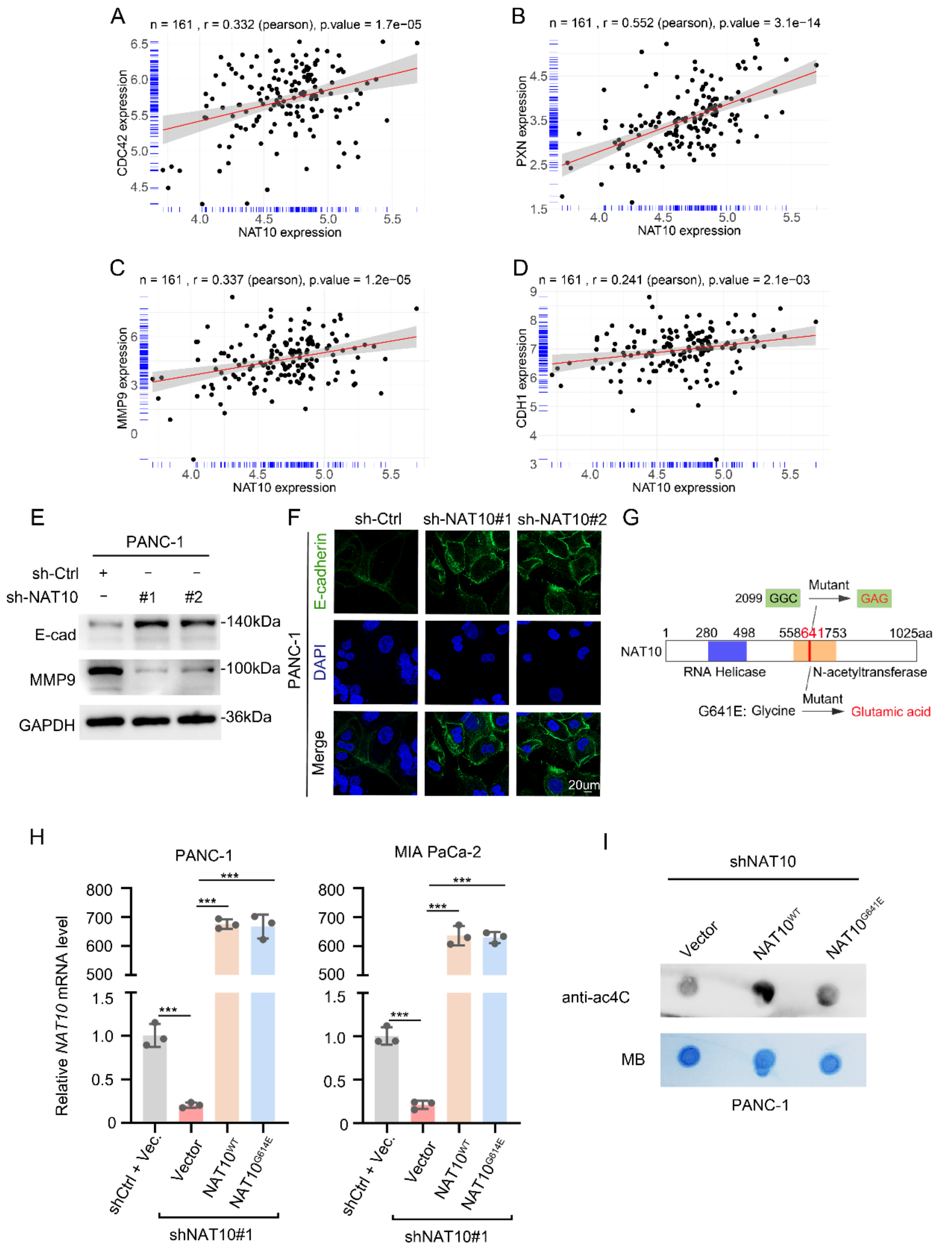

Supplementary Fig. 3 Related to Fig. 3

(A-D) Correlation between NAT10 expression and CDC42 (A), PXN (B), MMP9 (C), or CDH1 (D) from CPTAC database. **(E)** Immunoblots images of NAT10, E-cadherin, Matrix metalloproteinase-9 (MMP9) and GAPDH in NAT10 KD and control PANC-1 cells. **(F)** Immunofluorescent images of E-cadherin in NAT10 KD and control PANC-1 cells. Scale bar: 20 μm. **(G)** Schematic diagram for NAT10^{G641E} mutant. **(H)** Relative mRNA expression of NAT10 in NAT10 KD cells treated with overexpression of NAT10 wild-type (WT), overexpression of NAT10^{G641E} mutant, and control PDAC cells. **(I)**

RNA dot blot of ac4C level in NAT10 KD PDAC cells treated with overexpression of NAT10-WT and NAT10^{G641E} mutant. Methylene blue staining as loading control. *p<0.05, **p<0.01, ***p<0.001. (H), One-way ANOVA test.

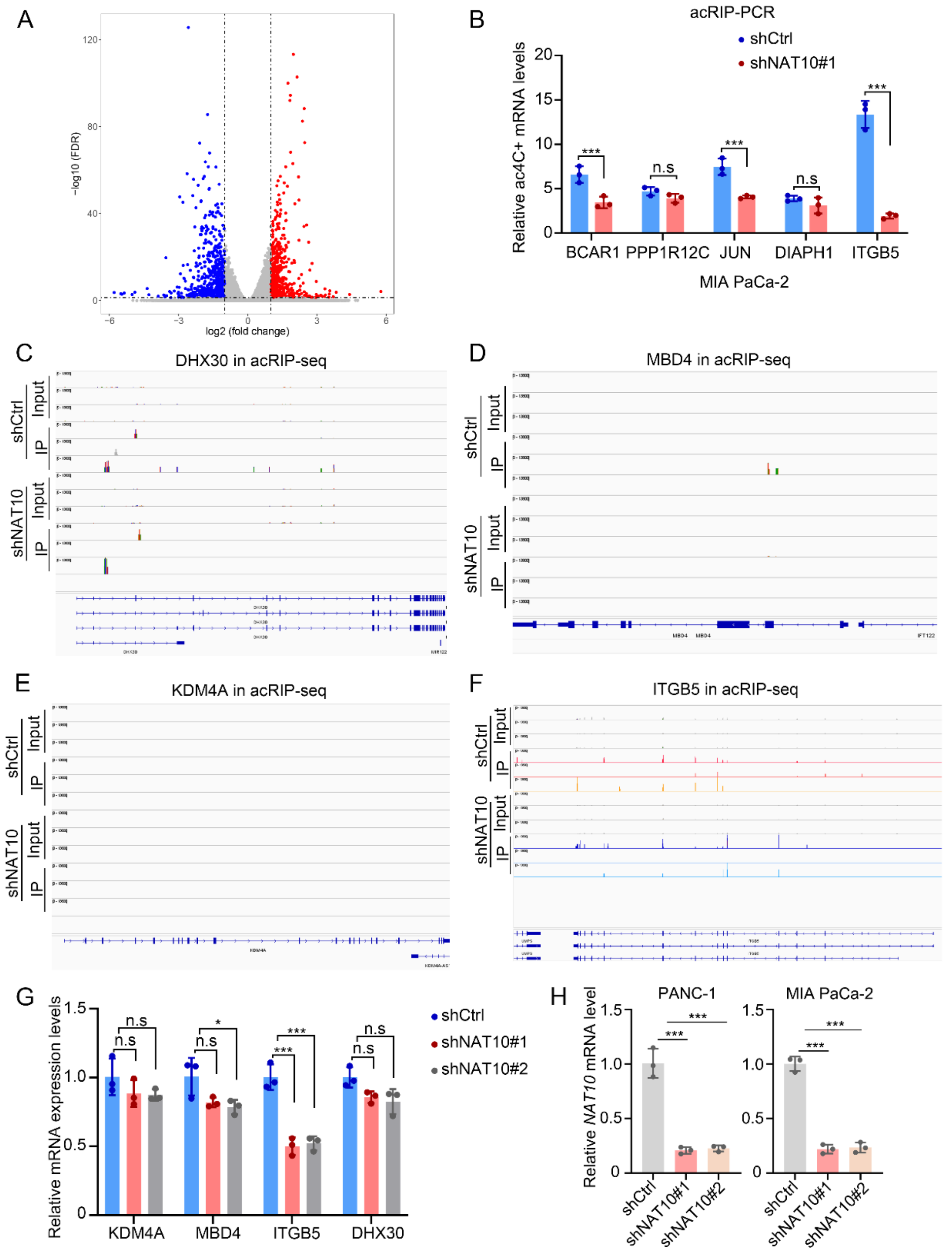

Supplementary Fig. 4 Related to Fig. 4

(A) Volcano plots of differentially expressed genes (DEGs) from RNA-seq (foldchange >2). n=1049 (423 upregulated and 626 downregulated genes). **(B)** acRIP analysis of merged genes from KEGG_focal adhesion pathway according to acRIP-seq and ac4C-seq. **(C-F)** Integrative Genomics Viewer (IGV) browser showing the ac4C

peaks in DHX30 (C), MBD4 (D), KDM4A (E) and ITGB5 (F). DHX30, DExH-Box Helicase 30; MBD4, Methyl-CpG Binding Domain 4; KDM4A, Lysine Demethylase 4A; ITGB5, Integrin Subunit Beta 5. **(G)** Relative mRNA expression of above genes in NAT10 KD and control PANC-1 cells. n=3. **(H)** Relative NAT10 mRNA expression in NAT10 KD and control PDAC cells. n=3. (B), and (G-H), One-way ANOVA test.

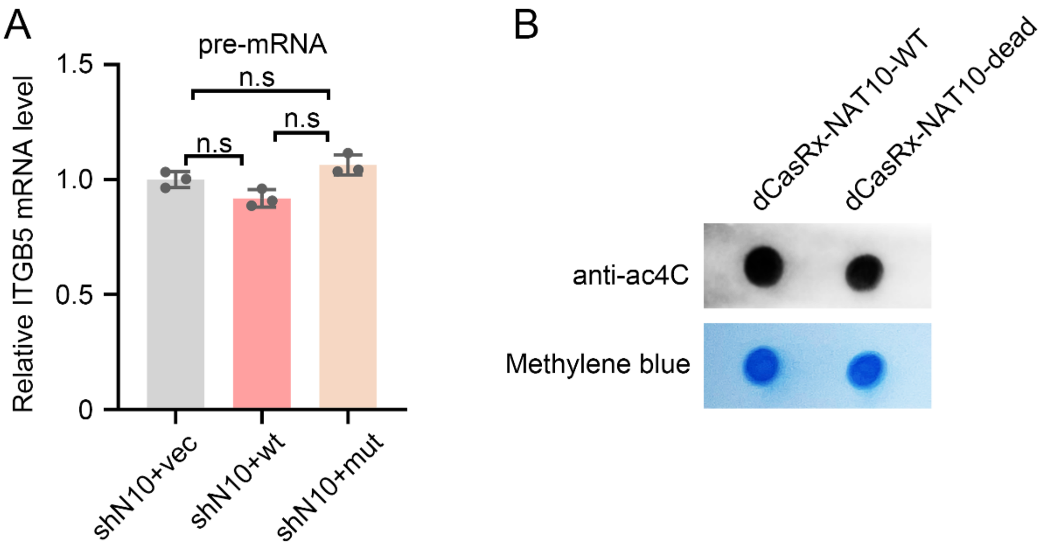

Supplementary Fig. 5 Related to Fig. 5

(A) qRT-PCR analysis showing the pre-mRNA level of ITGB5 in PANC-1 cells, which transfected with (1) NAT10 targeting shRNA (shN10) and vector plasmid (vec), (2) shN10 and overexpression NAT10 wild-type (wt) plasmid, or (3) shN10 and NAT10 G641E mutant (mut) plasmid. **(B)** RNA dot blot assay showing the ac4C level of total mRNA were not affected by dCasRx-NAT10-dead fusion protein. Methylene blue is as loading control. n.s, not significant. (A), One-way ANOVA test.

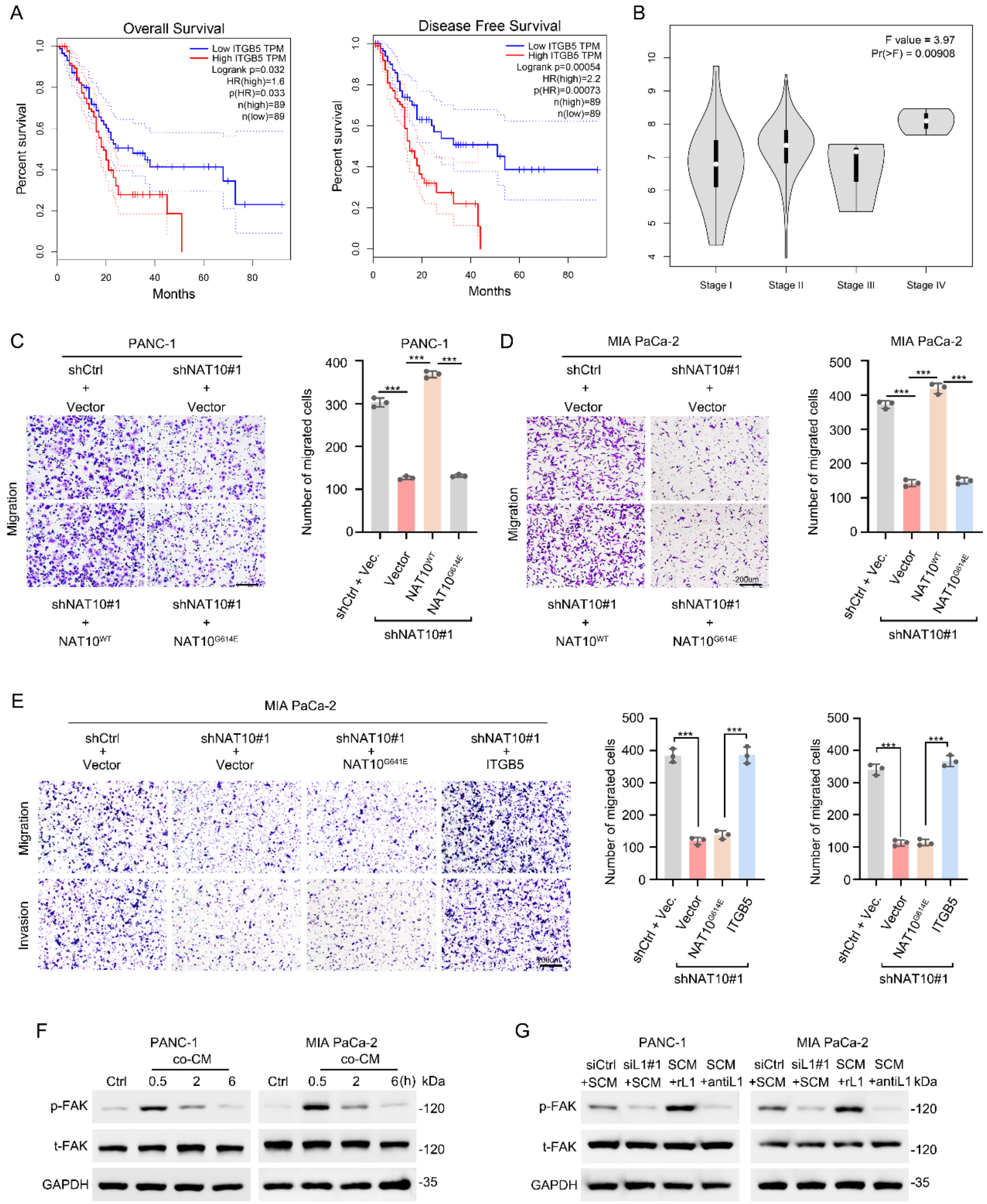

Supplementary Fig. 6 Related to Fig. 6

(A-B) Overall survival, disease-free survival analyses (A) and stage plot (B) correlated to ITGB5 expression were conducted using GEPIA on PAAD patients from TCGA database. **(C-D)** Representative image and quantification of Transwell migration in NAT10 G641E mutant of NAT10 KD PANC-1 (C) or MIA PaCa-2 (D) cells. Scale bar: 200 μm. **(E)** Representative image and quantification of Transwell in ITGB5 overexpression of NAT10 KD MIA PaCa-2. Scale bar: 200 μm. **(F)** Representative immunoblotting image of p-FAK and total-FAK in PANC-1 (left) and MIA PaCa-2 (right) after cocultured with medium from sNF 96.2 Schwann cells (co-CM) for indicated hours. **(G)** Representative immunoblotting image of p-FAK and total-FAK in

PDAC cells. PDAC cells were cocultured with Schwann cells condition medium (SCM) from sNF 96.2 treated with (i) siCtrl; (ii) siL1CAM#1, and PDAC cells treated with SCM and additive of (iii) human recombinant L1CAM and (iv) anti-L1CAM. *$p<0.05$, **$p<0.01$, ***$p<0.001$. (C-E), One-way ANOVA test.

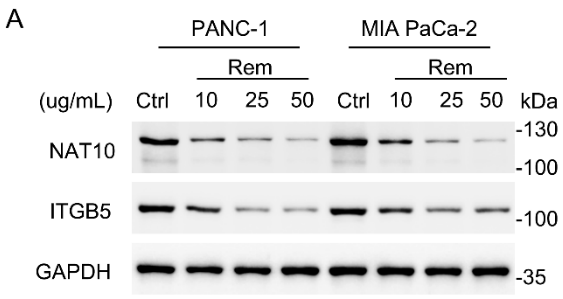

Supplementary Fig. 7 Related to Fig. 7

(A) Representative western blotting image in PANC-1 or MIA PaCa-2 cells treated with indicated concentration of Remodelin.

Supplementary Table

Table. S1 Clinicopathological correlation of NAT10 expression in PDAC patients from Sun Yat-sen Memorial Hospital Cohort.

Characteristics	Number of cases	NAT10 expression		*p*-value
		Low (n=30)	High (n=32)	
**Age (years)**				
<60	33	16(25.8%)	17(27.4%)	0.987
>=60	29	14(22.6%)	15(24.2%)	
**Gender**				
Male	34	14(22.6%)	20(32.3%)	0.211
Female	28	16(25.8%)	12(19.4%)	
**TNM**				
I-II	48	27(43.5%)	21(33.9%)	**0.022***
III-IV	14	3(4.8%)	11(17.7%)	
**Distant metastasis**				
No	59	29(46.8%)	30(48.4%)	1.000
Yes	3	1(1.6%)	2(3.2%)	
**Lymphovascular invasion**				
No	34	19(30.6%)	15(24.2%)	0.193
Yes	28	11(17.7%)	17(27.4%)	
**Lymph node metastasis**				
No	35	20(32.3%)	15(24.2%)	0.116
Yes	27	10(16.1%)	17(27.4%)	
**Perineural invasion**				
No	22	15(24.2%)	7(11.3%)	**0.021***
Yes	40	15(24.2%)	25(40.3%)	

- *Denotes statistical significance.
- *p*-values were calculated by Pearson's Chi-square test or Continuity Correction. *$p<0.05$.

Table. S2 Clinicopathological correlation of NAT10 expression in PDAC patients from Guangdong Provincial People's Hospital Cohort.

Characteristics	Number of cases	NAT10 expression		*p*-value
		Low (n=109)	High (n=123)	
**Age (years)**				
<60	90	48(20.7%)	42(18.1%)	0.123
>=60	142	61(26.3%)	81(34.9%)	
**Gender**				
Male	117	54(23.3%)	63(27.2%)	0.799
Female	115	55(23.7%)	60(25.9%)	
**TNM**				
I-II	187	90(38.8%)	97(41.8%)	0.476
III-IV	45	19(8.2%)	26(11.2%)	
**Distant metastasis**				
No	224	106(45.7%)	118(50.9)	0.852
Yes	8	3(1.3%)	5(2.2%)	
**Lymphovascular invasion**				
No	187	91(39.2%)	96(41.4%)	0.296
Yes	45	18(7.8%)	27(11.6%)	
**Lymph node metastasis**				
No	130	61(26.3%)	69(29.7%)	0.984
Yes	102	48(20.7%)	54(23.3%)	
**Perineural invasion**				
No	91	52(22.4%)	39(16.8%)	**0.013***
Yes	141	57(24.6%)	84(36.2%)	

- *Denotes statistical significance.
- *p*-values were calculated by Pearson's Chi-square test or Continuity Correction. *p<0.05.

Table. S3 Univariate and Multivariate analysis of Overall Survival (OS) in PDAC patients from Sun Yat-sen Memorial Hospital Cohort.

Variables	Univariate analysis			Multivariate analysis		
	HR	95%CI	*p*-value[A]	HR	95%CI	*p*-value[A]
Age (years) (<60 vs. >=60)	0.504	0.251-1.012	0.054			
Gender (Female vs. Male)	1.208	0.557-2.619	0.633			
TNM (III-IV vs. I-II)	3.482	1.184-10.237	**0.023***	2.756	1.170-6.492	**0.020***
Lymph node metastasis (Positive vs. Negative)	1.145	0.503-2.606	0.748			
Lymphovascular invasion (Positive vs. Negative)	1.846	0.855-3.989	0.119			
Perineural invasion (Positive vs. Negative)	2.505	1.129-5.560	**0.024***	2.411	1.074-5.414	**0.033***
Distance Metastasis (Positive vs. Negative)	10.141	2.172-47.338	**0.003****	4.816	1.272-18.230	**0.021***
NAT10 expression (High vs. Low)	2.669	1.194-5.965	**0.017***	2.779	1.330-5.808	**0.007****

- Abbreviations: HR = hazard ratio; 95%CI =95% confidence interval; TNM stage = tumor node metastasis stage; [A] Cox regression analysis.
- *p<0.05, **p<0.01.

Table. S4 Univariate and Multivariate analysis of Overall Survival (OS) in PDAC patients from Guangdong Provincial People's Hospital Cohort.

Variables	Univariate analysis			Multivariate analysis		
	**HR**	**95%CI**	***p*-value**[A]	**HR**	**95%CI**	***p*-value**[A]
Age (years) (<60 vs. >=60)	1.127	0.779-1.631	0.526			
Gender (Female vs. Male)	1.096	0.773-1.554	0.607			
TNM (III-IV vs. I-II)	0.860	0.543-1.361	0.519			
Lymph node metastasis (Positive vs. Negative)	1.476	0.969-2.248	0.070			
Lymphovascular invasion (Positive vs. Negative)	1.143	0.733-1.784	0.556			
Perineural invasion (Positive vs. Negative)	1.747	1.122-2.721	**0.014***	2.142	1.435-3.196	**<0.001*****
Distance Metastasis (Positive vs. Negative)	4.187	1.751-10.012	**0.001****	4.035	1.735-9.381	**0.001****
NAT10 expression (High vs. Low)	2.480	1.701-3.616	**<0.001*****	2.415	1.668-3.497	**<0.001*****

- Abbreviations: HR = hazard ratio; 95%CI =95% confidence interval; TNM stage = tumor node metastasis stage; [A] Cox regression analysis.
- *p<0.05, **p<0.01, ***p<0.001.

Table. S5 The sequence of oligonucleotides used in this study

**siRNAs**		
**Gene**	**Sense**	**Anti-sense**
Non-targeting siRNA	UUCUCCGAACGUGUCACGU	ACGUGACACGUUCGGAGAA
siNAT10#1	GCGGCAAAGAUCUCUCUUUTT	AAAGAGAGAUCUUUGCCGCTT
siNAT10#2	GCAUUUGGGUACUCCAAUATT	UAUUGGAGUACCCAAAUGCTT
siITGB5#1	GGAGGUUACUGAAUGACAAAC	UUGUCAUUCAGUAACCUCCUA
siITGB5#2	GCCAACGAGUACACUGCAUTT	AUGCAGUGUACUCGUUGGCTT
siL1CAM#1	GAACGGCAACCUCUACUUUTT	AAAGUAGAGGUUGCCGUUCTT
**shRNAs**	**Sequence (5' to 3')**	
Non-targeting shRNA	TTCTCCGAACGTGTCACGT	
NAT10 shRNA#1	GCTAGTGGTCATCCTCCTACG	
NAT10 shRNA#2	GCATTTGGGTACTCCAATATC	
**crRNA**	**Sequence (5' to 3')**	
Non-targeting sgRNA	TCACCAGAAGCGTACCATACTCACGAACAG	
ITGB5 sgRNA-1	GATCTTGCCAAACTCGCTCTCGAAGCAGGA	
ITGB5 sgRNA-2	CAGTGACACTCGCCATGGCCTGAGCAGAGG	
**Primer for qRT-PCR analysis**		
**Gene**	**Forward primer**	**Reverse primer**
GAPDH	GCTAAGCAGTTGGTGGTGCA	TCACCACCATGGAGAAGGC
β-actin	CATGTACGTTGCTATCCAGGC	CTCCTTAATGTCACGCACGAT
18S	GGCCCTGTAATTGGAATGAGTC	CCAAGATCCAACTACGAGCTT
NAT10	ATAGCAGCCACAAACATTCGC	ACACACATGCCGAAGGTATTG
ITGB5	TCTCGGTGTGATCTGAGGG	TGGCGAACCTGTAGCTGGA
pre-ITGB5	TCGGTTTGAGTGTGTGAG	TGAACTTGTTGAAGGTGAAG
L1CAM	TGTCATCACGGAACAGTCTCC	CTGGCAAAGCAGCGGTAGAT
GFAP	CTGCGGCTCGATCAACTCA	TCCAGCGACTCAATCTTCCTC
MBD4	CCGTCACCTCTAGTGAGCG	GCAGAAGCGATGGGTTCTTGTA
DHX30	CAGCCCAAAAATCTTCTCAACAG	GCTGCCATAGCCTTCTACCTC
KDM4A	GAAGCCACGAGCATCCTATGA	GCGGAACTCTCGAACAGTCA
JUN	TCCAAGTGCCGAAAAAGGAAG	CGAGTTCTGAGCTTTCAAGGT
PPP1R12C	CAGCCCTAAGAGTCCCGTG	GTCGGGGTAATTCTGGCAAGA
BCAR1	ATGGGCAGTACGAGAACAGC	GGCCAGGTCGTGGTCTATG
DIAPH1	CAGTTGGGTGCAAACATTTGG	TCCGGCTATCGTAACTCCCAG

Table. S6 The antibodies used in the experiments.

Product	Source	Catalogue
Mouse Monoclonal anti-GAPDH	Beijing Ray Antibody Biotech	RM2002
Mouse Monoclonal anti-β-tubulin	Beijing Ray Antibody Biotech	RM2003
Rabbit monoclonal [EPR18663] anti-NAT10	Abcam	ab194297
Rabbit polyclonal anti-NAT10	Proteintech	13365-1-AP
Rabbit monoclonal anti-Integrin beta 5	Cell Signaling Technology	3629
Rabbit polyclonal anti-Integrin beta 5	Proteintech	28543-1-AP
Mouse monoclonal anti-L1CAM	Abcam	ab24345
Mouse Monoclonal anti-CDH1	Cell Signaling Technology	14472
Rabbit monoclonal [EP1576Y] anti-S100B	Abcam	ab52642
Rabbit monoclonal [EP1580Y] to Cytokeratin 19	Abcam	ab52625
Rabbit monoclonal [EP1254] anti-MMP9	Abcam	ab76003
Rabbit polyclonal Phospho-FAK (Tyr397) antibody	Cell Signaling Technology	3283
Rabbit monoclonal Phospho-Src Family (Tyr416) antibody	Cell Signaling Technology	6943
Rabbit polyclonal anti-FAK	Cell Signaling Technology	3285
Rabbit monoclonal anti-Src	Cell Signaling Technology	2109
Rabbit monoclonal anti-PXN	Cell Signaling Technology	50195
Rabbit monoclonal anti-Cdc42	Cell Signaling Technology	2466
IgG from mouse serum	Sigma-Aldrich	I5381
IgG from rabbit serum	Sigma-Aldrich	I5006
Goat anti-Mouse IgG(H&L)-HRP	Beijing Ray Antibody Biotech	RM3001
Goat anti-Rabbit IgG(H&L)-HRP	Beijing Ray Antibody Biotech	RM3002
Rabbit monoclonal [EPRNCI-184-128] anti-ac4C	Abcam	ab252215
Mouse monoclonal anti-CDH1	Cell Signaling Technology	14472
Goat anti-Mouse IgG(H&L)-HRP	Abcam	ab205719
Goat anti-Rabbit IgG(H&L)-HRP	Abcam	ab205718
Goat Anti-Rabbit IgG H&L (Alexa Fluor® 488)	Abcam	ab150077

Goat Anti-Rabbit IgG H&L (Alexa Fluor® 555)	Abcam	ab150078
Goat Anti-Mouse IgG H&L (Alexa Fluor® 488)	Abcam	ab150113
