# Supplementary material for: N4-acetylcytidine modification of ITGB5 mRNA mediated by NAT10 promotes perineural invasion in pancreatic ductal adenocarcinoma
Source: J Exp Clin Cancer Res. 2025 Mar 22;44:103. doi: 10.1186/s13046-025-03362-2 (PMC11929334; doi:10.1186/s13046-025-03362-2)
Supplement: Supplementary file 4 — Supplementary Material 4 [file 13046_2025_3362_MOESM4_ESM.docx]

**Title:** **N^4^-acetylcytidine modification of *ITGB5* mRNA mediated by NAT10 promotes perineural invasion in pancreatic ductal adenocarcinoma**

**Authors:** Leyi Huang^1, 2, 3^†, Yanan Lu^4^†, Rihua He^1^†, Xiaofeng Guo^5^, Jiajia Zhou^2, 3^, Zhiqiang Fu^2,3^, Jingwen Li^6^, Jianping Liu^2,3^*, Rufu Chen^1^*, Yu Zhou^1^*, Quanbo Zhou^1, 7^*

**Affiliations:**

1 Department of Pancreas Center, Guangdong Provincial People's Hospital, Guangdong Academy of Medical Sciences, Southern Medical University, Guangzhou, Guangdong 519041, People’s Republic of China

2 Guangdong Provincial Key Laboratory of Malignant Tumor Epigenetics and Gene Regulation, Sun Yat-sen Memorial Hospital, Sun Yat-sen University, Guangzhou, Guangdong 510120, People’s Republic of China

3 Department of Pancreatobiliary Surgery, Sun Yat-sen Memorial Hospital, Sun Yat-sen University, Guangzhou, Guangdong 510120, People’s Republic of China

4 Department of Anesthesiology, Sun Yat-sen Memorial Hospital, Sun Yat-sen University, Guangzhou, Guangdong 510120, People’s Republic of China

5 Guangzhou Digestive Disease Centre, Guangzhou First People's Hospital, School of Medicine, South China University of Technology, Guangzhou, Guangdong 510180, People’s Republic of China.

6 Zhongshan School of Medicine, Sun Yat-sen University, Guangzhou, Guangdong 510275, People’s Republic of China.

7 Lead Contact

† These authors contributed equally to this work

*****Corresponding authors: Jianping Liu - [liuzhunlong@126.com](mailto:liuzhunlong@126.com), Rufu Chen - chenrufu@gdph.org.cn, Yu Zhou - [zhouyu@gdph.org.cn](mailto:zhouyu@gdph.org.cn), Quanbo Zhou - [zhouquanbo@gdph.org.cn](mailto:zhouquanbo@gdph.org.cn).

**Abstract**

**Background:** Perineural invasion (PNI) is a hallmark feature of pancreatic ductal adenocarcinoma (PDAC), which occurs at a high incidence and significantly contributes to PDAC lethality and poor survival. Despite its prevalence and association with poor prognosis, the molecular mechanisms underlying PNI in PDAC remain unclear.

**Methods:** We investigated clinical samples from two cohorts by UPLC/MS-MS to profiled significantly altered chemical RNA modifications in PDAC tissues with PNI lesions. Dorsal root ganglion coculture systems and sciatic nerve injection models validated PNI ability. We combined RNA-seq, acRIP-seq and ac4C-seq with CRISPR-based techniques to explore the regulatory mechanism of ac4C modification on the integrin beta 5 (*ITGB5*) transcript.

**Result:** We reported that N^4^-acetylcytidine (ac4C) is a significantly altered chemical RNA modification in PDAC tissues with PNI lesions. *In vitro* and *in vivo* models demonstrated that tumor cells overexpression of N-acetyltransferase 10 (NAT10), the writer enzyme of mRNA ac4C modification, enhances PNI in PDAC. Further analysis revealed decreased ac4C levels on transcripts of the focal adhesion pathway, particular on ITGB5, in NAT10-knockdown PDAC cells. This ac4C modification in the CDS region of ITGB5 mRNA promotes its stability, subsequently activating the ITGB5-pFAK-pSrc pathway. CRISPR-based analysis further confirmed the crucial role of NAT10-mediated ac4C modification in regulating ITGB5 expression. Combining small-molecule inhibitors targeting NAT10 and focal adhesion kinase (FAK) significantly attenuated PNI *in vivo*.

**Conclusion:** Our findings reveal a previously unrecognized ac4C-mediated epigenetic mechanism in PNI and propose a novel therapeutic strategy to improve survival in PDAC patients.

**One -sentence summary:** NAT10 promotes PNI via ac4C modification in PDAC

**Keywords**

Pancreatic ductal adenocarcinoma, Perineural invasion, RNA ac4C modification

**BACKGROUND**

Pancreatic ductal adenocarcinoma (PDAC) is a highly aggressive cancer with a dismal 5-year overall survival (OS) rate of only 13%[1]. Over 50% of patients are diagnosed at a locally advanced and unresectable stage, primarily due to the local invasion of adjacent structures[2]. Perineural invasion (PNI), a prominent feature of PDAC observed in nearly 100% of cases, represents a distinct mode of tumor dissemination and metastasis, ultimately causing poor clinical outcomes[3-5]. Despite its prevalence and importance, the mechanisms of PNI in PDAC remain poorly explored compared with other aspects of the tumor microenvironment. Recent advances in cancer neuroscience[6,7] highlight the crucial and complex role of tumor-nerve crosstalk in remodeling the tumor microenvironment and promoting tumor progression[8], offering new perspectives for understanding tumor biology and developing potential therapeutic interventions. Among solid tumors, PDAC has an exceptionally high rate of PNI, making it an ideal model for investigating the local remodeling of innervation by tumor cells. Understanding the regulatory mechanism of PNI is essential for developing effective therapeutic strategies and improving PDAC patient survival.

Epigenetic reprogramming is a crucial contributor to tumor plasticity and adaptation[9], including neural-related metastasis[10] and PNI[11]. Compared with DNA or histone modifications, RNA modifications offer unique opportunities for RNA-based therapies because of their efficacy and specificity[12]. Chemical RNA modifications, which are often dysfunctional in various tumors, provide a highly specific and efficient means of regulating RNA function, and present promising targets for cancer therapy[13]. For example, small-molecule inhibitors of N^6^-methyladenosine (m^6^A) modifiers, used alone or in combination therapies, have shown potential in preclinical cancer treatments[14]. Inhibition of m^6^A modification in mRNAs has also been reported to reduce PNI[15]. However, there are more than 170 types of RNA modifications, and the precise mechanisms by which alterations in RNA modification drive PNI in patients with PDAC remain unclear. Elucidating the epigenetic landscape of PDAC, specifically the role of RNA modification in PNI, could provide novel insights into the molecular mechanisms of PDAC aggressiveness and potentially identify new therapeutic strategies to inhibit PNI in patient with PDAC to improve survival.

In this study, we focused on the potential role and mechanism of altered RNA modification in PDAC patients with PNI. Using ultra-performance liquid chromatography/MS-MS (UPLC/MS-MS), we found that among 55 RNA modifications, N^4^-acetylcytidine (ac4C) was significantly altered in PDAC tissues from patients with PNI compared with those without PNI. ac4C is the first reported acetylated nucleoside and one of only three conserved cytidine modifications[16]. It was initially detected in tRNAs[17] and rRNAs[18] in eukaryotes and has recently been reported in mRNAs[19], where it promotes the progression of various tumors[20-23]. mRNA ac4C modification is catalyzed by the sole known writer, N-acetyltransferase 10 (NAT10)[19,21,22]. However, the oncogenic or tumor-suppressive role and underlying mechanism of the ac4C modification of mRNAs in PNI have not been reported.

Here, we identified significant alterations in ac4C modification in PDAC tissues with PNI. By elucidating the role and mechanism of aberrant mRNA ac4C modifications in PNI, this work aims to offer novel insights into the molecular mechanisms driving this process and to provide potential therapeutic strategies to reduce the aggressiveness of PDAC, ultimately improving patient outcomes and survival.

**MATERIALS AND METHODS**

**Patients and clinical samples**

Pancreatic ductal adenocarcinoma (PDAC) and adjacent tissues were collected from Sun Yat-sen Memorial Hospital (SYSMH) from January 2008 to July 2020, and from Guangdong Provincial People’s Hospital (GDPH) from January 2014 to July 2024. None of the patients received chemotherapy or radiotherapy before surgery. The detailed clinicopathological characteristics, univariate and multivariate analysis of SYSMH and GDPH cohorts are summarized in Table S1-4. The tumor-node-metastasis (TNM) stage was characterized in accordance with the 8^th^ edition of the American Joint Commission on Cancer (AJCC) guidelines. The study was approved by the Medical Ethics Committee of Sun Yat-sen Memorial Hospital (SYSEC-KY-KS-2021-177) and Guangdong Provincial People’s Hospital (KY2024-579-01), and informed consent for tissue collection was obtained from each patient.

**Animal experiments**

All the mice used in this study were grouped randomly and blindly. For the lung metastasis model, 3-5 weeks old female severe combined immunodeficient (SCID) mice were respectively injected with stably transduced luciferase vector PANC-1 cells (2×106 per mouse in 100μL PBS) into the tail vein. Six weeks after injection, the mice were anesthetized with isoflurane inhalation. VivoGlo luciferin (150mg/kg, Promega, USA) was injected i.p 10min before detection. The mice were imaged with an IVIS Spectrum Imaging System (Bruker, Germany) and IVScope 8500 (Clinx, China) to capture the image of lung metastasis.

In sciatic nerve invasion model, 3-5 weeks old female SCID mice were anesthetized, immobilized and surfaces sterilized, exposing the left and right sciatic nerves. PANC-1 lentivirus control (shCtrl), and knockdown NAT10 (shNAT10) cells (5×104) were respectively injected into left and right distal sciatic nerves under the epineurium using Hamiliton syringe. The mice were sacrificed after 28 days. The specimens were enucleated, embedded in paraffin or OCT, and examined via H&E staining, IHC or IF assay. The images were captured with Nikon Ni-U digital camera (Tokyo, Japan). All animal experiments were approved by the Animal Ethics Committee of the South China University of Technology (2021050).

Detailed methods and material used in this study were shown in the Supplementary Materials.

**RESULTS**

**ac4C modification and NAT10 levels are elevated in PNI of PDAC**

To explore the potential connection between RNA modification and PNI, we first analyzed RNA modifications in PDAC tissues with and without PNI using UPLC/MS-MS. Our finding from the Guangdong Provincial People’s Hospital (GDPH) cohort showed a significant upregulation of ac4C modification in the PNI group **(Fig. 1A)**. Consistent results were observed in the Sun Yat-sen Memorial Hospital (SYSMH) cohort **(Fig. 1B-C)**. RNA dot blot assays further confirmed higher ac4C levels in mRNA that isolated from PDAC tissues, particularly in those with PNI **(Fig. 1D)**.

We then explored the public databases of PDAC. Analysis with single-cell Hub 2 (TISCH2) and the Gene Expression Omnibus (GEO) database for PDAC (GSE154778) showed that NAT10, the only known RNA ac4C writer, was expressed primarily in malignant cells and correlated with increased pancreatic cancer risk **(Fig. S1A-B)**. *NAT10* levels were also increased in cancer tissues compared with those in non-tumor pancreas tissues, from the Genotype-Tissue Expression (GTEx), the Cancer Genome Atlas (TCGA), and GSE16515 datasets (**Fig. S1C-D**). NAT10 expression was positively correlated with known PDAC biomarkers, including MKI67, IMP3, MUC1, CLDN4, and KRAS, from the TCGA database **(Fig. S1E)**. High *NAT10* expression was correlated with worse OS, disease-free survival (DFS), and progression-free survival (PFS) from the TCGA and ArrayExpress (E-MTAB-6134) databases **(Fig. 1E-F)** and with worse pathological stage from the GEPIA database **(Fig. S1F-G)**.

To validate these findings, two independent cohorts from SYSMH and GDPH were included. In the SYSMH cohort, 57.6% of patients (33 pairs) expressed higher *NAT10* mRNA levels in tumor tissues than in adjacent normal pancreas tissues **(Fig. S1H)**. Immunofluorescence staining revealed that NAT10 staining was strongly positive in a large fraction of cytokeratin 19 (CK19) ^+^ tumor cells **(Fig. 1G)**. PNI is defined as the infiltration of tumor cells into nerve sheaths that occur within any of three anatomical layers: the epineurium, perineurium, and endoneurium[24]. Further analysis confirmed elevated NAT10 protein levels in tumor tissues **(Fig. 1H)**, especially in those with PNI **(Fig. 1I)**. We subsequently stained for NAT10 in clinical samples from patients with PDAC from both cohorts (n=62 from the SYSMH cohort, and n=232 from the GDPH cohort). We found that tumor cells with higher NAT10 expression had a greater propensity for nerve invasion **(Fig. 1J, Table S1-2)**. Patients with PNI exhibited higher NAT10 expression in tumor cells **(Fig. 1K-L)**.

Clinicopathological correlation analysis showed significant associations between high tumoral NAT10 and advanced tumor characteristics, including tumor node metastasis (TNM), lymph node metastasis and PNI in SYSMH cohort**,** and TNM and PNI in GDPH cohorts **(Table S1-2)**. Multivariate Cox analysis also revealed that high NAT10 expression and PNI were independent prognostic factor for OS in SYSMH and GDPH cohorts **(Table S3-4)**. Kaplan-Meier analysis further confirmed that higher NAT10 expression was significantly associated with poorer OS in PDAC patients (**Fig. 1M-N**). These data imply that elevated ac4C modification and its writer NAT10 are potential positive regulators in PDAC, correlating with poor prognosis and PNI progression.

**NAT10 knockdown (KD) impairs tumor migration, invasion and PNI in PDAC**

To elucidate the role of NAT10 in PDAC, we silenced NAT10 expression in human PDAC cell lines (i.e., PANC-1 and MIA PaCa-2 with naturally high endogenous NAT10 levels, **Fig. 2A**), using small interfering RNA (siRNA) and short hairpin RNA (shRNA). KD efficiencies were validated in PANC-1 and MIA PaCa-2 cells by quantitative reverse transcription polymerase chain reaction (qRT-PCR) and western blotting (**Fig. 2B-C, Fig. S2A-B**). Transwell assays showed that NAT10 KD using siRNA or shRNA could significantly attenuate tumor migration and invasion in PANC-1 (**Fig. 2D and Fig. S2C-D**) and MIA PaCa-2 (**Fig. 2E and Fig. S2E-F**) cells, indicating that NAT10 promotes PDAC migration and invasion *in vitro*.

Given the crucial role of PNI in tumor dissemination and its close association with poor prognosis in patients with PDAC[25], we next investigated the effect of stable NAT10 KD on the PNI ability of PDAC cells. We first isolated the dorsal root ganglion (DRG) from T8-T10 vertebrae of C57BL/6 mice and cocultured it with PDAC cells in Matrigel (**Fig. S2G**). By Day 6, NAT10 KD in PANC-1 and MIA PaCa-2 cells had significantly reduced tumor cells invasion towards the DRG (**Fig. 2F-G**). To further evaluate the PNI ability *in vivo*, we employed a murine sciatic nerve model of PNI using PANC-1 cells with or without NAT10 KD. Immunofluorescence analysis also showed that NAT10 was primarily expressed in tumor cells (**Fig. S2H**), as indicated above in human PDAC tissues. NAT10 KD significantly impaired the PNI process, indicating a decreased tumor infiltration into the endoneurium and perineurium of the nerve (**Fig. 2H**). Moreover, functional assays showed that NAT10 KD in PANC-1 significantly reduced the progressive ipsilateral hind limb paralysis on Day 28, as measured via the nerve function score **(Fig. 2I)** and sciatic nerve function index **(Fig. 2J)**. This finding suggests that NAT10 KD caused PNI suppression *in vivo*. These results indicate that NAT10 KD in tumor cells can impair PNI in PDAC.

**NAT10 expression in PDAC regulates focal adhesion in an ac4C-dependent manner**

NAT10 promotes tumor progression in an ac4C-dependent manner in various solid tumors[21,22,26]. To examine whether mRNA ac4C expression also participates in PDAC, we isolated mRNA from PDAC cell lines and confirmed that NAT10 KD in PANC-1 and MIA PaCa-2 cells significantly reduced ac4C mRNA levels (**Fig. 3A**). To further elucidate this mechanism, we used acRIP-seq and ac4C-seq assays in NAT10-KD and control PANC-1 cells. Both sequencing assays showed that most ac4C sites were found within classical consensus CXXCXX motifs (**Fig. 3B**). The NAT10-KD group displayed a significant reduction in cumulative ac4C abundance (**Fig. 3C**). Consistent with previous reports[19,21,22,27], ac4C marks were predominantly accumulated in the CDS regions (**Fig. 3D**).

Considering the susceptibility of false positives with conventional acRIP-seq and its inability to detect ac4C at nucleotide resolution[28], we combined it with single-nucleotide resolution ac4C-seq in NAT10-KD PANC-1 cells, to find the potential target of NAT10-modified mRNAs. In this method, ac4C was reduced to tetrahydro-ac4C using NaBH_4_, a borohydride that induce C to T mismatches at acetylated sites[20,29,30], a single nucleotide variation (SNV). Similar to acRIP-seq, the ac4C-seq data further showed that the major fraction of ac4C sites were within the CDS region (**Fig. 3E**). We observed a decreased number of C to T SNV within the CDS in the NAT10-KD group (**Fig. 3F**) as previously reported[20]. Given the functional changes following NAT10 KD, Kyoto Encyclopedia of Genes and Genomes (KEGG) enrichment analysis of pathways was performed, highlighting pathways such as focal adhesion and gap junction, which are strongly associated with cancer cell migration and invasion (**Fig. 3G-H**).

We next explored the correlation between NAT10 expression and the focal adhesion pathway activation. Analysis of 161 PDAC cases from the Clinical Proteomic Tumor Analysis Consortium (CPTAC) database revealed that NAT10 mRNA expression was positively correlated with CDC42, matrix metalloproteinase-9 (MMP9), and Paxillin (PXN), which are expressed upon the focal adhesion pathway activation **(Fig. S3A-C)**. Epithelial-to-mesenchymal transition (EMT) can be activated by focal adhesion pathway activation, which leads to cell migration. While NAT10 mRNA levels are positively correlated with CDH1 mRNA levels from database **(Fig. S3D)**, we found that NAT10-KD led to upregulation of the suppressor E-cadherin and reduction of the oncogene MMP9 **(Fig. S3E-F)**, indicating a potential role in activating FAK-Src signaling downstream.

FAK, a tyrosine kinase activated by integrin in the focal adhesion pathway, is a crucial signaling component and regulator of tumor cell migration, adhesion, and motility. Tyr397 phosphorylation of FAK facilitates Src binding, causing Src activation and a dual-activated FAK-Src signaling complex[31]. We observed that NAT10 KD inhibited FAK (Tyr397) and Src (Tyr416) phosphorylation, as well as the expression of downstream proteins in the focal adhesion pathway (**Fig. 3I**). To validate the role of RNA acetylation in FAK-Src activation, we rescued NAT10 with wild-type (NAT10^WT^) or catalytic (glycine-641 to glutamate)[27,32] NAT10 (NAT10^G641E^) mutant in NAT10-KD cells (**Fig. S3G**). The mutation showed no significant impact on NAT10 expression, as revealed by qRT-PCR (**Fig. S3H**). RNA dot blot assays showed that mRNA ac4C levels was reversed by ectopically expressing NAT10^WT^ but slightly by NAT10^G641E^ in NAT10-KD cells (**Fig. S3I**). FAK and Src phosphorylation were reduced upon NAT10-KD, which was rescued by reintroducing NAT10^WT^, but not NAT10^G641E^ (**Fig. 3J**). Dual immunofluorescence assays in PANC-1 and MIA PaCa-2 further confirmed that overexpression of NAT10^WT^, but not the catalytic mutant NAT10^G641E^, restored the inhibitory effect of NAT10 silencing on FAK and Src phosphorylation (**Fig. 3K-L**). Therefore, these data suggest that NAT10 promotes the activation of the focal adhesion pathway by its catalytic activity in acetylation.

**ITGB5 is the downstream target of ac4C modification in PDAC cells**

To investigate the transcriptional changes mediated by NAT10 in PDAC, we performed RNA-seq on stable NAT10-KD PANC-1 cells (**Fig. S4A**). Gene-set enrichment analysis (GSEA) of the GSE16515 database indicated that the focal adhesion pathway was elevated in high NAT10-expressing samples (**Fig. 4A**). Conversely, our RNA-seq data demonstrated a marked suppression of cell adhesion molecules in NAT10-KD PANC-1 cells (**Fig. 4B**). Gene Ontology (GO) analysis of downregulated transcriptions further confirmed the inhibition of several migratory pathways, including cell adhesion mediated by integrin (**Fig. 4C**). A heatmap of downregulated transcripts in the focal adhesion pathway from our RNA-seq data showed several key genes including *PXN*, *ITGB5* and *CDC42*(**Fig. 4D**), known genes activating the FAK-Src pathway and regulating cell migration[33,34]. These findings collectively suggested that transcription in the focal adhesion pathway is regulated by NAT10, potentially through ac4C modification.

To identify specific targets within the focal adhesion pathway regulated by ac4C modification, we overlapped acRIP-seq and ac4C-seq data, focusing on ac4C-positive genes from the focal adhesion pathway based on KEGG enrichment results **(Fig. 3G-H)**. This analysis highlighted *BCAR1*, *JUN*, *ITGB5*, *PPP1R12C*, and *DIAPH1* as potential candidates **(Fig. 4E)**. acRIP assays confirmed decreased mRNA ac4C enrichment for *ITGB5*, *JUN* and *BCAR1* in NAT10-KD cells **(Fig. S4B)**. This indicated that the focal adhesion pathway is regulated by ac4C modification. To further narrow down our transcriptional targets, we combined these sequencing data with our RNA-seq data, specifically examining downregulated transcripts associated with hypoacetylation (FDR<0.05, log2 (FC) <-1), which revealed a set of genes, including *MBD4*, *ITGB5*, *DHX30*, and *KDM4A* **(Fig. 4F)**, with *ITGB5* exhibiting the highest ac4C peak **(Fig. S4C-F)** among these transcripts. qRT-PCR analysis revealed that *ITGB5* exhibited the pronounced differences in mRNA level alterations **(Fig. S4G)**. FAK activation is critically dependent on integrin-mediated cell adhesion and Src activity triggered by integrin[31]. ITGB5 encodes the β5-subunit of the integrin, a transmembrane receptor that exclusively pairs the β5 subunit with the αv subunit, promotes tumor progression[35] and is associated with poor prognosis in PDAC[36]. ITGB5 have been reported to mediate FAK activity, promoting the Src phosphorylation[37]. Furthermore, we confirmed that ITGB5 mRNA expression (**Fig. 4G and Fig. S4H**) and protein expression (**Fig. 4H**) were significantly downregulated in NAT10-KD cells. Consistently, analysis of CPTAC databases showed a positive correlation between NAT10 and ITGB5 **(Fig. 4I)**, further supporting their relationship. Thus, we selected ITGB5 for downstream analysis.

To validate ITGB5 as a key target of ac4C modification, we employed the Analysis of Integrative Genomics Viewer browser and found two ac4C peaks within the CDS region of *ITGB5* mRNA in the control group, which decreased in the NAT10-KD group (**Fig. 4J**). acRIP assays validated that ac4C enrichment of *ITGB5* transcript was reduced upon NAT10-KD in PANC-1 and MIA PaCa-2 cells (**Fig. 4K**). To investigate the direct relationship between ITGB5 expression and NAT10-catalyzed acetylation, we assessed ITGB5 levels and found that overexpression of NAT10^WT^, but not its catalytically inactive NAT10^G641E^ mutant, markedly increased ITGB5 expression at both mRNA (**Fig. 4L**) and protein (**Fig. 4M**) levels when endogenous NAT10 was reduced. Consistently, the ac4C level of *ITGB5* was increased in the NAT10^WT^ but not in the NAT10^G641E^ group (**Fig. 4N**). These data collectively indicate that NAT10-mediated ac4C modification is crucial for regulation of ITGB5 expression, establishing ITGB5 as a key downstream target.

**Knockdown of ac4C modification mediated by NAT10 expedites the degradation of ITGB5 mRNA**

Given the established significance of NAT10-mediated ac4C in regulating ITGB5 expression, we initially assessed the impact of NAT10 on ITGB5 pre-mRNA and mature mRNA levels. While mature ITGB5 mRNA levels decreased in NAT10-KD PANC-1 cells, pre-mRNA levels showed slight changes (**Fig. 5A**). Similarly, overexpression of NAT10^WT^ or NAT10^G641E^ showed minimal changes in pre-mRNA (**Fig. S5A**), suggesting that NAT10 might primarily affect post-transcriptional processes. NAT10-mediated mRNA ac4C modification within the CDS can promote mRNA stability[19,27]. We treated PDAC cells with actinomycin D (Act. D) to inhibit transcription. We observed that NAT10-KD caused a faster *ITGB5* decay in PANC-1 and MIA PaCa-2 cells (**Fig. 5B**), indicating that NAT10 stabilized *ITGB5* mRNA. Polysome profiling analysis indicated that an elevated 80S peak coincided with diminished polysomes in NAT10-KD cells, suggesting a global translation reduction when NAT10 was knocked down (**Fig. 5C**). Further analysis of RNA fractions isolated using a sucrose density gradient showed decreased *ITGB5* mRNA translation in NAT10-KD cells (**Fig. 5D**).

To further confirm whether the stability of *ITGB5* mRNA is regulated by NAT10-mediated ac4C modification, we used a deactivated dCasRx-based ac4C-editing platform. Cytoplasmic mRNA decay is a significant post-transcriptional mechanism[38]. Similar to a previous study[39], we generated dCasRx fusion protein with either NAT10 or acetylase-dead NAT10 G641E (NAT10-dead), and designed two specific guide CRISPR RNAs (sgRNAs) targeting the *ITGB5* mRNA CDS region at different distances to C2032 target cytosine site (**Fig. 5E**). We first confirmed the expression and cytosolic localization of dCasRx-NAT10 and dCasRx-NAT10-dead through western blotting and immunofluorescence (**Fig. 5F-G**). To further assess whether site-specific ac4C modification was influenced, NAT10-KD PANC-1 and MIA PaCa-2 cells were transfected with plasmids expressing dCasRx-NAT10, dCasRx-NAT10-dead plus sgRNA-a, sgRNA-b, or control sgRNA. dCasRx-NAT10 overexpression slightly affected the ac4C levels of total mRNA when compared with those in the dCasRx-NAT10-dead group (**Fig. S5B**). Notably, compared to dCasRx-NAT10 and control sgRNA group, co-transfection of dCasRx-NAT10-dead with specific sgRNA-a/b caused a diminished ac4C modification of *ITGB5* mRNA (**Fig. 5H**). Concomitantly, the mature *ITGB5* mRNA was remarkedly downregulated (**Fig. 5I**) when functional sgRNA-a and dCasRx-NAT10-dead were in NAT10-KD PDAC cells, which was accompanied by a shorter *ITGB5* mRNA half-life (**Fig. 5J**) and decreased ITGB5 protein expression (**Fig. 5K**). These findings suggest that NAT10-mediated ac4C modification is crucial for maintaining *ITGB5* mRNA stability in PDAC cells.

**NAT10-mediated ac4C modification of ITGB5 promotes the migration, invasion, and metastasis of PDAC cells**

Previous studies have reported that upregulated expression of ITGB5 in tumor cells promote metastasis in PDAC[36,40]. ITGB5 was positively correlated with worse survival **(Fig. S6A)** and pathological stage **(Fig. S6B)** in PDAC patients according to public databases. We next examined the role of NAT10 mediated acetylation in PDAC cells. Transwell assays showed that overexpression of NAT10^WT^, but not NAT10^G641E^, enhanced the migratory **(Fig. S6C-D**) and invasive (**Fig. 6A-B)** abilities of PANC-1 and MIA PaCa-1 cells. Given our finding that ITGB5 is the downstream target of NAT10-mediated ac4C modification, we sought to investigate the function of the NAT10-ITGB5 axis in PDAC cells. We first overexpressed ITGB5 in NAT10-KD PANC-1 and MIA PaCa-2 cells (**Fig. 6C)**. Transwell assays revealed that the migration and invasion abilities of PDAC cells, inhibited by NAT10-KD or catalytical mutant, were rescued by ITGB5 overexpression (**Fig. 6D and Fig. S6E**).

To examine the PNI ability, we used a coculture system. Compared with NAT10-KD cells, the invasion of tumor cells toward DRG and tumor proliferation were significantly increased when ITGB5 was overexpressed (**Fig. 6E**). To further investigate if the NAT10-ac4C-ITGB5 axis could promote tumor metastasis *in vivo*, we generated luciferase-PANC-1 cells and injected the labeled-cells into the tail vein of severe combined immunodeficiency (SCID) mice. After 4 weeks of tumor inoculation, in vivo imaging was performed. Bioluminescence imaging showed that ITGB5 overexpression rescued the lung metastasis of NAT10-KD or catalytic mutant NAT10 PANC-1 cells (**Fig. 6F-G**). Similarly, enhanced lung metastatic tumors were also observed in mice from the ITGB5 overexpression group (**Fig. 6H-I**), suggesting that ITGB5 overexpression rescues the aggressive phenotype in NAT10-KD PDAC cells.

Integrin αvβ5, a membrane receptor, is characterized by its ability to bind with high affinity to ligands presenting an RGD (-Arg-Gly-Asp-) motif[41], such as L1CAM[42]. Soluble L1CAM promotes the migration of CHO cells by interacting with integrin αvβ5[43], acting as a vital mediator of cell motility. Schwann cells release soluble L1CAM to facilitate PNI in PDAC[44]. We previously found that the tumor-neuroglia cell coculture did not change *L1CAM* mRNA expression[4]. To assess the role of soluble L1CAM secreted by Schwann cells in NAT10-KD tumor cells, we collected the culture medium from human sNF96.2 Schwann cells (SCM) and cocultured it with PDAC cells. We observed that SCM-treated PDAC cells had elevated p-FAK levels (**Fig. S6F**). Furthermore, when we silenced L1CAM in Schwann cells, PDAC cells incubated with SCM from L1CAM-KD Schwann cells or with the addition of human recombined L1CAM, showed rescued FAK phosphorylation, while the anti-L1CAM blocked the effect on p-FAK (**Fig. S6G**). These findings suggest that NAT10 promotes PDAC migration, invasion, and metastasis via ITGB5 expression, highlighting the importance of the NAT10-ITGB5-focal adhesion axis in PDAC progression.

**NAT10-ITGB5-focal adhesion axis promotes PNI in PDAC**

Remodelin (Rem), a small-molecule inhibitor of NAT10, has been reported to inhibit tumor progression[26]. Our western blotting results revealed that Rem treatment diminished NAT10 and ITGB5 expression levels (**Fig. S7A**). ITGB5 is an essential transmembrane receptor in the focal adhesion pathway. Considering the potential for other compensatory mechanisms that could influence integrin-FAK signaling when only NAT10 is inhibited, we employed both NAT10 and FAK inhibitors to explore their potential therapeutic efficacy for PNI. Defactinib (DFN) is a novel FAK inhibitors that inhibits FAK phosphorylation at the Tyr397 site in PDAC[45]. Based on our above evidence implicating the NAT10-ITGB5 axis in promoting the progression of PDAC and PNI through activation of the focal adhesion pathway, we hypothesized that combining Rem with DFN may be a potential therapeutic strategy for PDAC.

To investigate the effects of Rem+DFN treatment on PNI, we cultured DRG in Matrigel and placed tumor cells over the matrix-DRG assay[46]. Sixteen hours after the tumor cell cultivation, the cocultured system was treated with dimethyl sulfoxide Rem, DFN, or Rem and DFN daily for 10 days. We observed that the combination treatment with Rem and DFN caused less tumor cell invasion in the combined treatment group than in the vehicle group (**Fig. 7A-B**). Next, to investigate the efficacy of combination treatment *in vivo*, we used immune-competent C57/Bl6 mice. Mouse pancreatic tumor cells isolated from Trp53^em4(R172H)^ Kras^em4(LSL-G12D^ Tg(Pdx1-cre)Smoc (KPC) genetically engineered mice were injected into the sciatic nerve. One week after tumor inoculation, mice were treated for 2 weeks with Rem, DFN, or a combination of both (**Fig. 7C**). Dual immunofluorescence analysis revealed that combination treatment markedly inhibited the infiltration of CK19^+^ tumor cells into the endoneurium and perineurium of the S100^+^ nerve (**Fig. 7D-E**).

We further employed multiple IHC (mIHC) staining to examine PNI and ITGB5 expression in human PDAC samples. Notably, we observed that in patients with high NAT10 expression, ITGB5 expression in CK19^+^ PDAC cells was positively associated with a pro-invasive phenotype towards S100B^+^ Schwann cells (**Fig. 7F-G**). By targeting both the upstream modifier and the downstream effector, we provide an approach to potentially impede PDAC progression and PNI. These data indicate that the NAT10-ITGB5-focal adhesion axis is a potential therapeutic target against PNI in PDAC.

**DISCUSSION**

PNI is a special mode of tumor dissemination and indicates poor survival in various gastrointestinal cancers[47], it is recognized as a crucial hallmark and a poor prognostic feature in patients with PDAC. While recent studies have suggested that epigenetic mechanisms in cancer neuroscience promote tumor metastasis[10,15], the specific mechanism of the RNA modification underlying PNI remains unclear. Here, we have demonstrated a significant role of NAT10 in regulating ac4C modification to promote the development of PNI. Our data show that NAT10 expression in tumor cells promotes PNI via the ac4C-ITGB5-pFAK-pSrc axis, highlighting the crucial role of ac4C modification in PNI, providing a therapeutic target for preventing PNI and improving the prognosis of patients with PDAC.

Tumor-induced PNI can be observed in over 80% of patients with PDAC, significantly impacting their prognosis. Distant metastasis of pancreatic cancer deprives patients of the potential benefits of radical surgery. Most patients with PDAC are diagnosed at an unresectable stage, and only 20% of newly diagnosed patients are available for curable resection[48], prompting the identification of pro-metastatic factors within tumor cells that could impede PNI and enhance the surgical resectability and survival of patients with PDAC. Moreover, our previous multicenter randomized controlled trial[49] in pancreatic head cancer, demonstrated improved DFS in patients who received extended pancreatoduodenectomy with modified retroperitoneal nerve resection compared to standard pancreatoduodenectomy, underscoring the importance of addressing PNI as a crucial aspect of pancreatic cancer intervention.

Recent advances in the field of cancer neuroscience[7,50,51] have demonstrate the complex mechanism of PNI, which may be induced by the inherent increased tumor cell invasiveness and the interaction with soluble substances received from Schwann cells. Previous studies have reported that RNA ac4C modification and its acetyltransferase NAT10 contributes to tumor metastasis in various solid tumors [21-23] which have great propensity to nerve invasion, suggesting a potential association between high NAT10 expression and increased tumor PNI potential. Upregulated NAT10 levels were also observed in mice with peripheral nerve injury[52], which is crucial in regulating the development of neuropathic pain. Here, we provide evidence that NAT10 promotes the expression of ITGB5, a vital receptor in the focal adhesion pathway, thereby activating this signaling pathway to promote tumor invasion. In addition, ITGB5 act as a receptor for soluble L1CAM, which is released by Schwann cells[42,44], triggering tyrosine phosphorylation of FAK and Src, synergistically promoting PNI in PDAC. These findings provide a deeper understanding of the regulatory mechanisms associated with local remodeling of innervation by tumor cells in PDAC.

RNA epitranscriptomics is a rapidly emerging field that is inspiring oncology research. The dynamic and reversible chemical modifications of RNAs provide highly specific and efficient means to regulate the biological function of tumor cells[13][13]. However, mRNA modifications have only been discovered and characterized in the last few decades and remain largely unknown. We found that ac4C modification exhibits the most significant alteration in PDAC with PNI **(Fig. 1A-B)**. The mRNA ac4C modification is catalyzed by the sole known N-acetyltransferase NAT10. Although the NAT10 signaling pathway reportedly acts as a pro-oncogenic factor in pancreatic cancer[23], the specific cytosine sites within mRNA regulated by NAT10 in PNI remain unreported. Exploring specific RNA epigenetic targets and their intervention strategies is essential because it may help develop reversible and highly targeted approaches for tumor metastasis. To address the limitation of conventional acRIP-seq, which have susceptibility of false positives and inability to detect ac4C at nucleotide resolution[28], we combined ac4C-seq with single-nucleotide resolution. By integrating transcriptomics (acRIP-seq, ac4C-seq, and RNA-seq), we found that NAT10 facilitates ac4C modification within the coding region of ITGB5 mRNA, enhancing its stability and causing subsequent upregulation of the key membrane receptor ITGB5 in the focal adhesion pathway. Similar to a previous study [39], we specifically interfered with the potential ac4C-modified cytosine (C2032) in the CDS region of ITGB5 mRNA using a dCasRx-based ac4C editing tool, revealing the significant impact of ac4C modification on ITGB5 mRNA stability and expression. Here, we expand the current understanding of RNA chemical modification in cancer neuroscience by demonstrating that NAT10 expression in tumor cells promotes the progression of PNI and metastatic phenotypes in PDAC.

Previous studies[12,53] have highlighted the clinical perspectives of targeting aberrant RNA modification in human gastrointestinal cancer. For example, various small-molecule inhibitors targeting RNA N^6^-methyladenosine modification have been demonstrated to reduce the progression of intrahepatic cholangiocarcinoma[54] and synergistically inhibit colon cancer with anti-PDL1 blockade[55] in mouse models. Moreover, clinical studies showed that FAK inhibitors will be more effective in combination with other agents[56]. Multiple clinical trials with a combination of FAK inhibitors and other agents remain in progress[56], while a phase II trial has shown no activity in combination with the MEK inhibitor trametinib in patients with advanced PDAC[57], indicating that further studies are required to explore anticancer combinations. Our findings suggest that the depletion of NAT10-mediated ac4C modification, including using NAT10 inhibitors include Rem, could significantly reduce tumor progression when combined with other small-molecular inhibitors or FDA-approved drugs. We reported that the combination of NAT10 inhibitors Rem, and FAK small-molecular inhibitors DFN significantly reduced PNI in the DRG-matrix model and *in vivo*.

Despite our study revealing an unreported role and mechanism of ac4C in PNI, which stabilizes *ITGB5* mRNA and activates the focal adhesion pathway, there are several limitations to consider. Because no eraser or reader of ac4C modification has been reported to date, further studies are required to explore the regulatory mechanism underlying RNA ac4C modification, and to provide a better strategy for depletion of ac4C, such as dCasRx fused with an ac4C eraser. Our research primarily relies on PDAC cell lines and two cohorts of patients, which may not fully represent the broader range of PDAC patients. Additionally, as the limitation of the present *in vivo* model of PNI, further studies using genetically modified mouse models with PNI *in situ* are needed to exploring the tumor-neuro microenvironment mediated by ac4C and assessing its therapeutic potential in real-world setting. Moreover, the safety, efficacy, and optimal delivery methods for targeting ac4C of *ITGB5* mRNA in patients present significant challenges for clinical translation.

**CONCLUSION**

In summary, we showed that NAT10 in tumor cells drive PNI progression in PDAC by mediating mRNA ac4C modification. Our study refined our understanding of the role of NAT10 in PNI and confirmed the potential of a previously unappreciated therapeutic target in ameliorating PDAC metastasis.

**LIST OF ABBREVIATIONS**

PDAC: Pancreatic ductal adenocarcinoma

PNI: Perineural invasion

ac4C: N^4^-acetylcytidine

acRIP: RNA immunoprecipitation using ac4C antibody

NAT10: N-acetyltransferase 10

GSEA: Gene Set Enrichment Analysis

TCGA: The Cancer Genome Atlas

GTEx: Genotype-Tissue Expression

GEO: Gene Expression Omnibus

CPTAC: Clinical Proteomic Tumor Analysis Consortium

ITGB5: Integrin beta 5

**DECLARATIONS**

**Ethics approval and consent to participate**

This study was approved by the Medical Ethics Committee of Guangdong Provincial People’s Hospital (KY2024-579-01), and Sun Yat-sen Memorial Hospital (SYSEC-KY-KS-2021-177) and informed consent for tissue collection was obtained from each patient. All animal experiments were approved by the Animal Ethics Committee of the South China University (2021050).

**Consent for publication**

The consent from all authors was obtained.

**Availability of data and material**

We deposited RNA-seq and acRIP-seq data to GSE185960, and ac4C-seq data to GSE275963. All other data are available on reasonable request to the corresponding author.

**Competing interests**

The authors declare no potential conflicts of interest.

**Funding**

This study was supported by grants from the National Natural Science Foundation of China (82073149, 82472893), and the Natural Science Foundation of Guangdong Province, China (2022A1515220219), and Research Funding for Full-time Talent Recruitment of Guangdong Provincial People’s Hospital (KY012023369).

**Authors' contributions**

Conceptualization, LH, YZ, and QZ; Methodology, LH, YZ, and QZ; Formal analysis, LH, YL, RH; Investigation, LH, YL, RH, XG, ZF, JZ, JWL, JPL, RC, YZ, and QZ; Resource, JPL, RC, YZ, and QZ; Funding acquisition, QZ; Validation, LH, YL, RH; Visualization, LH, YL, RH; Writing – original draft, LH, YL, RH; Writing – review and editing, LH, JPL, YZ, and QZ; Project administration, JPL, RC, YZ, and QZ; Supervision, QZ.

**References**

[1] Siegel RL, Giaquinto AN, Jemal A. Cancer statistics, 2024. CA Cancer J Clin 2024;74(1):12-49. 10.3322/caac.21820.

[2] Balaban EP, Mangu PB, Khorana AA, Shah MA, Mukherjee S, Crane CH, et al. Locally advanced, unresectable pancreatic cancer: american society of clinical oncology clinical practice guideline. J Clin Oncol 2016;34(22):2654-68. 10.1200/JCO.2016.67.5561.

[3] Amit M, Na'Ara S, Gil Z. Mechanisms of cancer dissemination along nerves. Nat Rev Cancer 2016;16(6):399-408. 10.1038/nrc.2016.38.

[4] Su D, Guo X, Huang L, Ye H, Li Z, Lin L, Chen R, Zhou Q. Tumor-neuroglia interaction promotes pancreatic cancer metastasis. Theranostics 2020;10(11):5029-5047. 10.7150/thno.42440.

[5] Zhang B, Guo X, Huang L, Zhang Y, Li Z, Su D, et al. Tumour-associated macrophages and schwann cells promote perineural invasion via paracrine loop in pancreatic ductal adenocarcinoma. Br J Cancer 2024;130(4):542-554. 10.1038/s41416-023-02539-w.

[6] Hanahan D, Monje M. Cancer hallmarks intersect with neuroscience in the tumor microenvironment. Cancer Cell 2023;41(3):573-580. 10.1016/j.ccell.2023.02.012.

[7] Winkler F, Venkatesh HS, Amit M, Batchelor T, Demir IE, Deneen B, et al. Cancer neuroscience: state of the field, emerging directions. Cell 2023;186(8):1689-1707. 10.1016/j.cell.2023.02.002.

[8] Mancusi R, Monje M. The neuroscience of cancer. Nature 2023;618(7965):467-479. 10.1038/s41586-023-05968-y.

[9] Lomberk G, Dusetti N, Iovanna J, Urrutia R. Emerging epigenomic landscapes of pancreatic cancer in the era of precision medicine. Nat Commun 2019;10(1):3875. 10.1038/s41467-019-11812-7.

[10] Pascual G, Dominguez D, Elosua-Bayes M, Beckedorff F, Laudanna C, Bigas C, et al. Dietary palmitic acid promotes a prometastatic memory via schwann cells. Nature 2021;599(7885):485-490. 10.1038/s41586-021-04075-0.

[11] Hurnik P, Chyra Z, Sevcikova T, Stembirek J, Trtkova KS, Gaykalova DA, Buchtova M, Hruba E. Epigenetic regulations of perineural invasion in head and neck squamous cell carcinoma. Front Genet 2022;13:848557. 10.3389/fgene.2022.848557.

[12] Delaunay S, Helm M, Frye M. Rna modifications in physiology and disease: towards clinical applications. Nat Rev Genet 2024;25(2):104-122. 10.1038/s41576-023-00645-2.

[13] Barbieri I, Kouzarides T. Role of rna modifications in cancer. Nat Rev Cancer 2020;20(6):303-322. 10.1038/s41568-020-0253-2.

[14] Deng X, Qing Y, Horne D, Huang H, Chen J. The roles and implications of rna m(6)a modification in cancer. Nat Rev Clin Oncol 2023;20(8):507-526. 10.1038/s41571-023-00774-x.

[15] Li F, He C, Yao H, Zhao Y, Ye X, Zhou S, et al. Glutamate from nerve cells promotes perineural invasion in pancreatic cancer by regulating tumor glycolysis through hk2 mrna-m6a modification. Pharmacol Res 2023;187:106555. 10.1016/j.phrs.2022.106555.

[16] Karthiya R, Wasil SM, Khandelia P. Emerging role of n4-acetylcytidine modification of rna in gene regulation and cellular functions. Mol Biol Rep 2020;47(11):9189-9199. 10.1007/s11033-020-05963-w.

[17] Sharma S, Langhendries JL, Watzinger P, Kotter P, Entian KD, Lafontaine DL. Yeast kre33 and human nat10 are conserved 18s rrna cytosine acetyltransferases that modify trnas assisted by the adaptor tan1/thumpd1. Nucleic Acids Res 2015;43(4):2242-58. 10.1093/nar/gkv075.

[18] Ito S, Horikawa S, Suzuki T, Kawauchi H, Tanaka Y, Suzuki T, Suzuki T. Human nat10 is an atp-dependent rna acetyltransferase responsible for n4-acetylcytidine formation in 18 s ribosomal rna (rrna). J Biol Chem 2014;289(52):35724-35730. 10.1074/jbc.C114.602698.

[19] Arango D, Sturgill D, Alhusaini N, Dillman AA, Sweet TJ, Hanson G, et al. Acetylation of cytidine in mrna promotes translation efficiency. Cell 2018;175(7):1872-1886.e24. 10.1016/j.cell.2018.10.030.

[20] Arango D, Sturgill D, Yang R, Kanai T, Bauer P, Roy J, Wang Z, Hosogane M, Schiffers S, Oberdoerffer S. Direct epitranscriptomic regulation of mammalian translation initiation through n4-acetylcytidine. Mol Cell 2022;82(15):2797-2814.e11. 10.1016/j.molcel.2022.05.016.

[21] Liao L, He Y, Li SJ, Yu XM, Liu ZC, Liang YY, et al. Lysine 2-hydroxyisobutyrylation of nat10 promotes cancer metastasis in an ac4c-dependent manner. Cell Res 2023;33(5):355-371. 10.1038/s41422-023-00793-4.

[22] Zhang Y, Jing Y, Wang Y, Tang J, Zhu X, Jin W, Wang Y, Yuan W, Li X, Li X. Nat10 promotes gastric cancer metastasis via n4-acetylated col5a1. Signal Transduction and Targeted Therapy 2021;6(1):173. 10.1038/s41392-021-00489-4.

[23] Zong G, Wang X, Guo X, Zhao Q, Wang C, Shen S, et al. Nat10-mediated axl mrna n4-acetylcytidine modification promotes pancreatic carcinoma progression. Exp Cell Res 2023;428(2):113620. 10.1016/j.yexcr.2023.113620.

[24] Liebig C, Ayala G, Wilks JA, Berger DH, Albo D. Perineural invasion in cancer: a review of the literature. Cancer-Am Cancer Soc 2009;115(15):3379-91. 10.1002/cncr.24396.

[25] Bapat AA, Hostetter G, Von Hoff DD, Han H. Perineural invasion and associated pain in pancreatic cancer. Nat Rev Cancer 2011;11(10):695-707. 10.1038/nrc3131.

[26] Wei W, Zhang S, Han H, Wang X, Zheng S, Wang Z, et al. Nat10-mediated ac4c trna modification promotes egfr mrna translation and gefitinib resistance in cancer. Cell Rep 2023;42(7):112810. 10.1016/j.celrep.2023.112810.

[27] Tsai K, Jaguva Vasudevan AA, Martinez Campos C, Emery A, Swanstrom R, Cullen BR. Acetylation of cytidine residues boosts hiv-1 gene expression by increasing viral rna stability. Cell Host Microbe 2020;28(2):306-312.e6. 10.1016/j.chom.2020.05.011.

[28] Thalalla GS, Sas-Chen A, Schwartz S, Meier JL. Quantitative nucleotide resolution profiling of rna cytidine acetylation by ac4c-seq. Nat Protoc 2021;16(4):2286-2307. 10.1038/s41596-021-00501-9.

[29] Sas-Chen A, Thomas JM, Matzov D, Taoka M, Nance KD, Nir R, et al. Dynamic rna acetylation revealed by quantitative cross-evolutionary mapping. Nature 2020;583(7817):638-643. 10.1038/s41586-020-2418-2.

[30] Thalalla GS, Sas-Chen A, Schwartz S, Meier JL. Quantitative nucleotide resolution profiling of rna cytidine acetylation by ac4c-seq. Nat Protoc 2021;16(4):2286-2307. 10.1038/s41596-021-00501-9.

[31] Mitra SK, Hanson DA, Schlaepfer DD. Focal adhesion kinase: in command and control of cell motility. Nat Rev Mol Cell Biol 2005;6(1):56-68. 10.1038/nrm1549.

[32] Larrieu D, Britton S, Demir M, Rodriguez R, Jackson SP. Chemical inhibition of nat10 corrects defects of laminopathic cells. Science 2014;344(6183):527-32. 10.1126/science.1252651.

[33] Mclean GW, Carragher NO, Avizienyte E, Evans J, Brunton VG, Frame MC. The role of focal-adhesion kinase in cancer - a new therapeutic opportunity. Nat Rev Cancer 2005;5(7):505-15. 10.1038/nrc1647.

[34] Webb DJ, Donais K, Whitmore LA, Thomas SM, Turner CE, Parsons JT, Horwitz AF. Fak-src signalling through paxillin, erk and mlck regulates adhesion disassembly. Nat Cell Biol 2004;6(2):154-61. 10.1038/ncb1094.

[35] Hurtado DMT, Mose ES, Botta GP, Braun GB, Kotamraju VR, French RP, et al. Tumor-penetrating therapy for beta5 integrin-rich pancreas cancer. Nat Commun 2021;12(1):1541. 10.1038/s41467-021-21858-1.

[36] Su D, Wang R, Chen G, Ding C, Liu Y, Tao J, et al. Fbxo32 stimulates protein synthesis to drive pancreatic cancer progression and metastasis. Cancer Res 2024. 10.1158/0008-5472.CAN-23-3638.

[37] Pang X, He X, Qiu Z, Zhang H, Xie R, Liu Z, Gu Y, Zhao N, Xiang Q, Cui Y. Targeting integrin pathways: mechanisms and advances in therapy. Signal Transduct Target Ther 2023;8(1):1. 10.1038/s41392-022-01259-6.

[38] Schoenberg DR, Maquat LE. Regulation of cytoplasmic mrna decay. Nat Rev Genet 2012;13(4):246-59. 10.1038/nrg3160.

[39] Liu XM, Zhou J, Mao Y, Ji Q, Qian SB. Programmable rna n(6)-methyladenosine editing by crispr-cas9 conjugates. Nat Chem Biol 2019;15(9):865-871. 10.1038/s41589-019-0327-1.

[40] Su L, Chen Y, Huang C, Wu S, Wang X, Zhao X, et al. Targeting src reactivates pyroptosis to reverse chemoresistance in lung and pancreatic cancer models. Sci Transl Med 2023;15(678):eabl7895. 10.1126/scitranslmed.abl7895.

[41] Hatley R, Macdonald S, Slack RJ, Le J, Ludbrook SB, Lukey PT. An alphav-rgd integrin inhibitor toolbox: drug discovery insight, challenges and opportunities. Angew Chem Int Ed Engl 2018;57(13):3298-3321. 10.1002/anie.201707948.

[42] Romano NH, Madl CM, Heilshorn SC. Matrix rgd ligand density and l1cam-mediated schwann cell interactions synergistically enhance neurite outgrowth. Acta Biomater 2015;11:48-57. 10.1016/j.actbio.2014.10.008.

[43] Mechtersheimer S, Gutwein P, Agmon-Levin N, Stoeck A, Oleszewski M, Riedle S, et al. Ectodomain shedding of l1 adhesion molecule promotes cell migration by autocrine binding to integrins. J Cell Biol 2001;155(4):661-73. 10.1083/jcb.200101099.

[44] Na'Ara S, Amit M, Gil Z. L1cam induces perineural invasion of pancreas cancer cells by upregulation of metalloproteinase expression. Oncogene 2019;38(4):596-608. 10.1038/s41388-018-0458-y.

[45] Le Large T, Bijlsma MF, El HB, Mantini G, Lagerweij T, Henneman AA, et al. Focal adhesion kinase inhibition synergizes with nab-paclitaxel to target pancreatic ductal adenocarcinoma. J Exp Clin Cancer Res 2021;40(1):91. 10.1186/s13046-021-01892-z.

[46] Huyett P, Gilbert M, Liu L, Ferris RL, Kim S. A model for perineural invasion in head and neck squamous cell carcinoma. J Vis Exp 2017(119). 10.3791/55043.

[47] Vaes N, Idris M, Boesmans W, Alves MM, Melotte V. Nerves in gastrointestinal cancer: from mechanism to modulations. Nat Rev Gastroenterol Hepatol 2022;19(12):768-784. 10.1038/s41575-022-00669-9.

[48] Speer AG, Thursfield VJ, Torn-Broers Y, Jefford M. Pancreatic cancer: surgical management and outcomes after 6 years of follow-up. Med J Aust 2012;196(8):511-5. 10.5694/mja11.10890.

[49] Lin Q, Zheng S, Yu X, Chen M, Zhou Y, Zhou Q, et al. Standard pancreatoduodenectomy versus extended pancreatoduodenectomy with modified retroperitoneal nerve resection in patients with pancreatic head cancer: a multicenter randomized controlled trial. Cancer Commun (Lond) 2023;43(2):257-275. 10.1002/cac2.12399.

[50] Amit M, Na'Ara S, Gil Z. Mechanisms of cancer dissemination along nerves. Nat Rev Cancer 2016;16(6):399-408. 10.1038/nrc.2016.38.

[51] Vaes N, Idris M, Boesmans W, Alves MM, Melotte V. Nerves in gastrointestinal cancer: from mechanism to modulations. Nat Rev Gastroenterol Hepatol 2022;19(12):768-784. 10.1038/s41575-022-00669-9.

[52] Zhang M, Yang K, Wang QH, Xie L, Liu Q, Wei R, et al. The cytidine n-acetyltransferase nat10 participates in peripheral nerve injury-induced neuropathic pain by stabilizing syt9 expression in primary sensory neurons. J Neurosci 2023;43(17):3009-3027. 10.1523/JNEUROSCI.2321-22.2023.

[53] Lin S, Kuang M. Rna modification-mediated mrna translation regulation in liver cancer: mechanisms and clinical perspectives. Nat Rev Gastroenterol Hepatol 2024;21(4):267-281. 10.1038/s41575-023-00884-y.

[54] Xu QC, Tien YC, Shi YH, Chen S, Zhu YQ, Huang XT, Huang CS, Zhao W, Yin XY. Mettl3 promotes intrahepatic cholangiocarcinoma progression by regulating ifit2 expression in an m(6)a-ythdf2-dependent manner. Oncogene 2022;41(11):1622-1633. 10.1038/s41388-022-02185-1.

[55] Liu Y, Liang G, Xu H, Dong W, Dong Z, Qiu Z, et al. Tumors exploit fto-mediated regulation of glycolytic metabolism to evade immune surveillance. Cell Metab 2021;33(6):1221-1233.e11. 10.1016/j.cmet.2021.04.001.

[56] Dawson JC, Serrels A, Stupack DG, Schlaepfer DD, Frame MC. Targeting fak in anticancer combination therapies. Nat Rev Cancer 2021;21(5):313-324. 10.1038/s41568-021-00340-6.

[57] Aung KL, Mcwhirter E, Welch S, Wang L, Lovell S, Stayner LA, et al. A phase ii trial of gsk2256098 and trametinib in patients with advanced pancreatic ductal adenocarcinoma. J Gastrointest Oncol 2022;13(6):3216-3226. 10.21037/jgo-22-86.

**FIGURE LEGENDS**

**
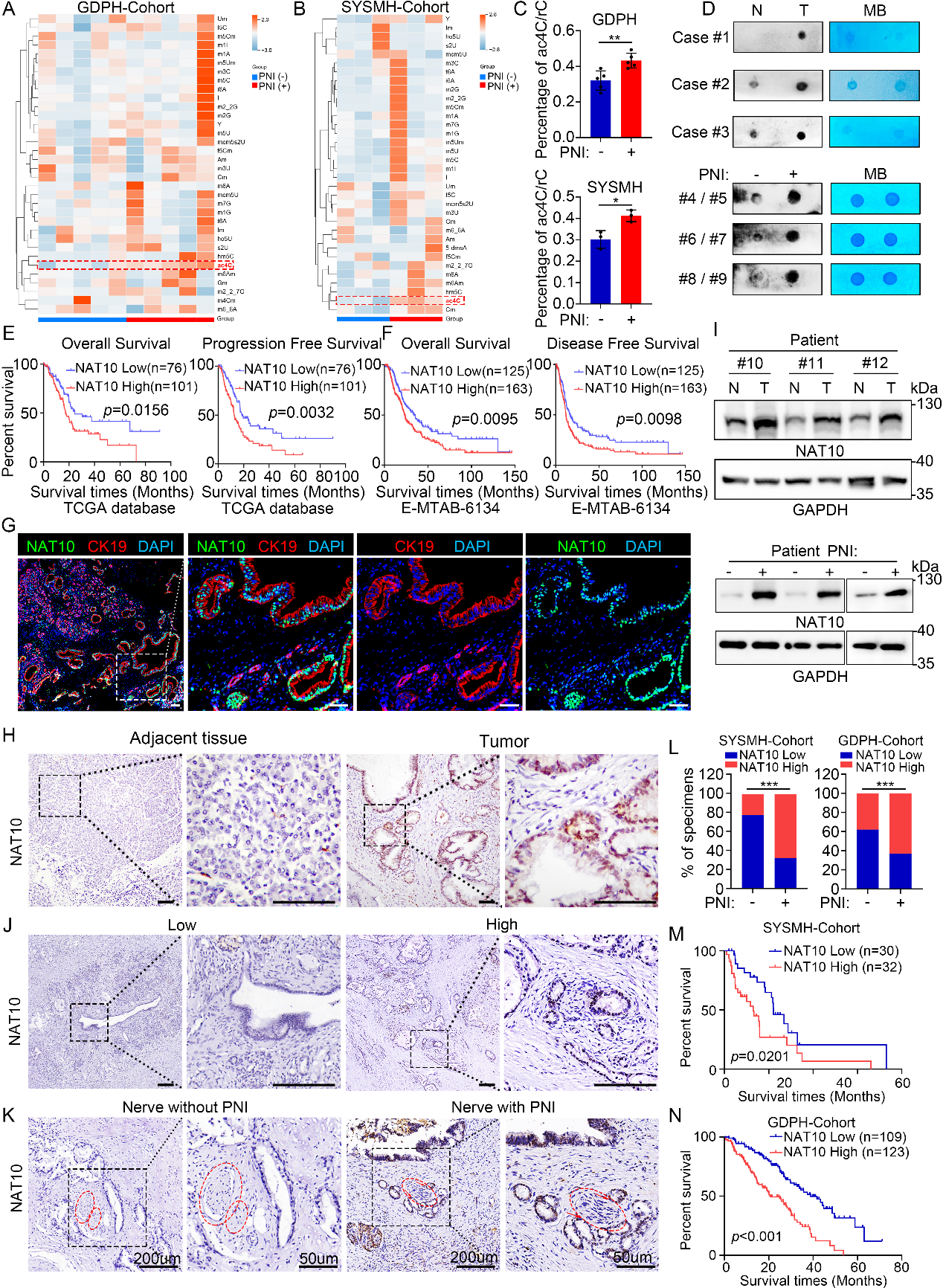
**

**Fig. 1. Elevated ac4C modification and increased NAT10 expression are correlated with enhanced PNI and poor prognosis in PDAC. (A-B)** Heatmap of UPLC/MS-MS showing upregulation of RNA ac4C levels in PDAC tissues with PNI compared to those without PNI from GDPH cohort (A, *p*=0.007) and SYSMH-cohort (B, *p*=0.023). **(C)** Quantification of ac4C level in PDAC tissue with or without PNI from UPLC/MS-MS analysis. **(D)** RNA dot blot assays indicating a consistent increase in ac4C levels in three paired PDAC tissues (T) when compared to levels in paired normal (N) pancreas (upper panel). The lower panel compares between PDAC tissues with PNI and those without PNI. **(E)** Kaplan-Meier curves for OS and PFS in patients with PAAD from TCGA database with higher (n=101) or lower (n=76) expression of NAT10 mRNA. **(F)** Kaplan-Meier curves for OS and DFS in patients with pancreatic cancer from the E-MTAB-6134 database with higher (n=163) or lower (n=125) expression of NAT10 mRNA. **(G)** Representative immunofluorescence staining of NAT10 (Green) and CK19 (Red) in PDAC. The nuclei are stained with DAPI (blue). Scale bar, 50 μm. **(H)** Representative immunohistochemistry images of NAT10 protein in clinical PDAC tissues. Scale bar: 50 μm. **(I)** Immunoblotting of NAT10 in three paired samples of PDAC and normal tissue (upper), and another six PDAC tissues with (n=3) or without (n=3) PNI (lower). **(J)** Representative immunohistochemistry images of NAT10 in PDAC cases from our cohort with low and high expression. Red circle, perineural nerve in the pancreas. Scale bar: 50 μm. **(K)** Representative immunohistochemistry images of NAT10 staining in clinical samples with or without PNI. Red circle indicates the nerve. Scale bar: 200 μm (left); 50 μm (right). **(L)** Percentages of PNI and NAT10 expression in PDAC from different clinical cohorts. n=62 in the SYSMH cohort; n=232 in the GDPH cohort. **(M-N)** Kaplan-Meier analysis for OS in patients with PDAC from the SYSMH (M) and GDPH (N) cohort with higher or lower NAT10 expression. **p*<0.05, ***p*<0.01, ****p*<0.001. (C), Student’s t-test. (E-F) and (M-N), log-rank test. (L), χ^2^ test.


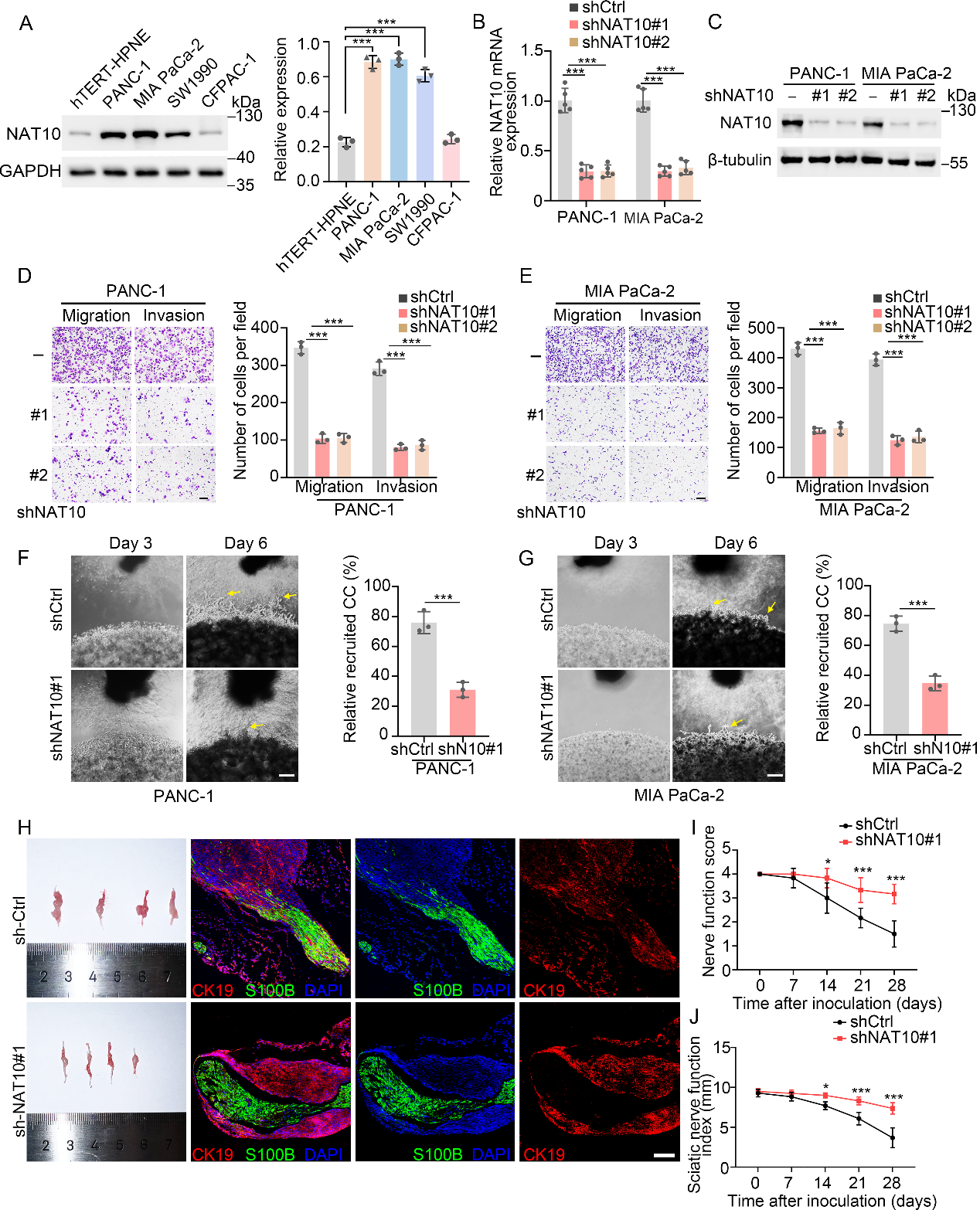


**Fig. 2.** **NAT10 KD impairs tumor migration, invasion, and PNI in PDAC. (A)** Representative immunoblots (left) and quantification (right) of NAT10 in human pancreatic ductal epithelial (hTERT-HPNE) and human PDAC cell lines. **(B-C)** Relative mRNA expression (B) and representative immunoblots (C) of NAT10 in NAT10 KD (via shRNA) and control PANC-1 or MIA PaCa-2 cells. **(D-E)** Representative image and quantification of Transwell migration and invasion in NAT10 KD and control PANC-1 (D) or MIA PaCa-2 (E) cells. Scale bar: 200 μm. **(F-G)** PANC-1 (F) or MIA PaCa-2 (G) cells were cocultured with DRGs into Matrigel. Representative images (left) on Day 3 and 6 are shown. The arrowhead indicates tumor cell invasions. Quantification (right) of relative recruited cancer cells on Day 6 is shown. Scale bar: 200 μm. (**H**) NAT10 KD or control PANC-1 cells were injected into the sciatic nerve of SCID mice (n=4). Representative immunofluorescence image of the Schwann cell marker S100β and tumor marker CK19 reveals nerve invasion of NAT10-KD or control PANC-1 cells. Scale bar: 100 μm. **(I-J)** Nerve function score (hind limb function, I) and sciatic nerve function index (hind paw span, J) were measured every 7 days. **p*<0.05, ***p*<0.01, ****p*<0.001. (A-B), and (D-E), One-way ANOVA. (F-G), Student’s t-test.


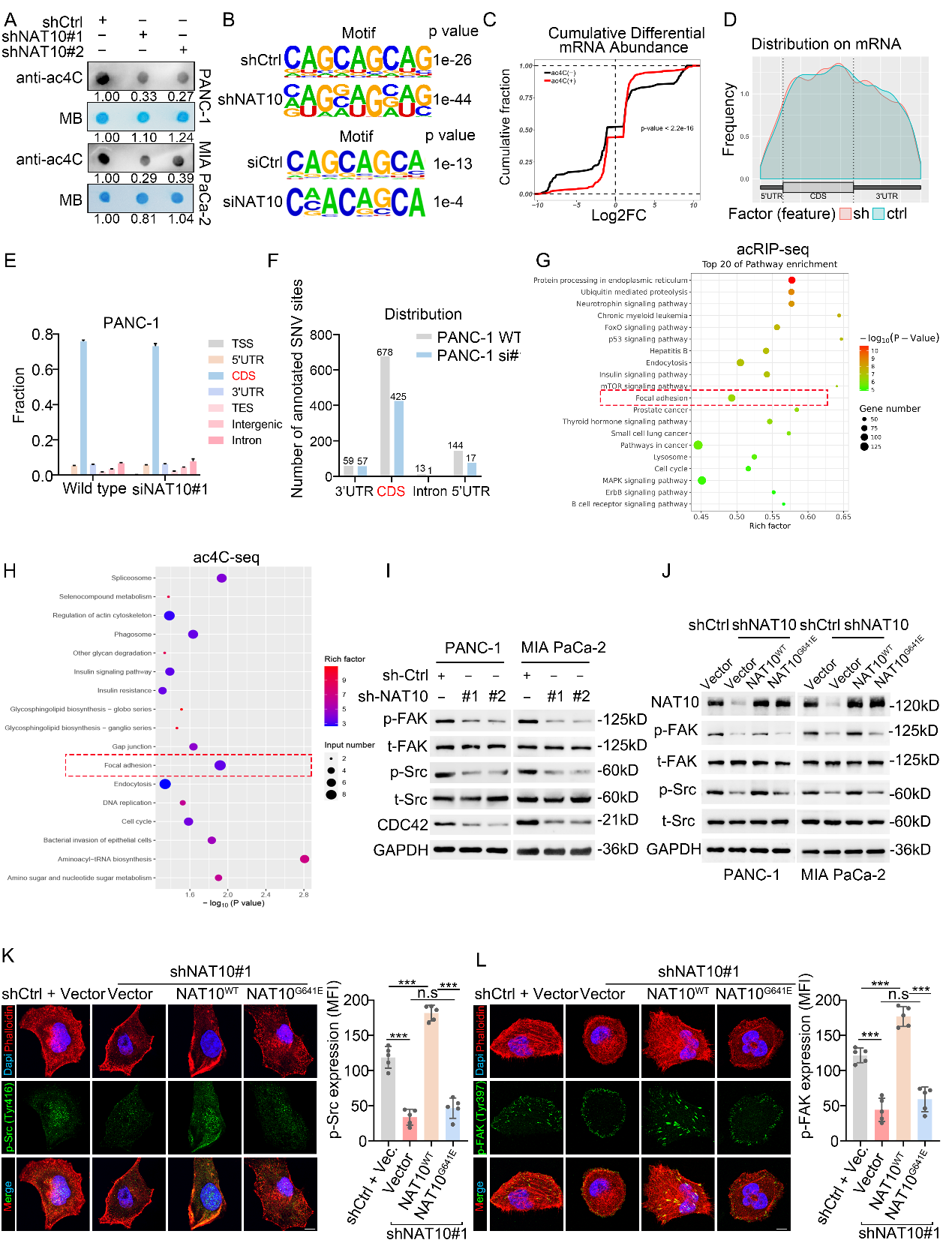


**Fig. 3. NAT10 expression in PDAC regulates focal adhesion in an ac4C-dependent manner. (A)** RNA dot blot of ac4C level in NAT10-KD PDAC cells. Methylene blue was used as a loading control. **(B)** HOMER motif analysis for ac4C sites with acRIP-seq (upper) and ac4C-seq (lower) in NAT10 KD and control PANC-1 cells. **(C)** Cumulative distribution showing differential expression of ac4C transcripts in NAT10 KD and control PANC-1 cells, based on acRIP-seq. Kolmogorov-Smirnov test (n=3). **(D)** Frequency distribution of ac4C+ transcript in PANC-1 cells from acRIP-seq. **(E)** Read distribution in ac4C-seq. n=3. **(F)** Number of C to T single nucleotide variation in ac4C-seq. **(G-H)** Kyoto Encyclopedia of Genes and Genomes (KEGG) pathway analysis of acRIP-seq and ac4C-seq data indicates the enrichment of the focal adhesion pathway. **(I)** Immunoblot images of FAK, Src, phospho-FAK, phospho-Src, CDC42 and GAPDH, in NAT10 KD and control PDAC cells. **(J)** Immunoblotting image of NAT10, FAK, Src, phospho-FAK, phospho-Src and GAPDH, in NAT10 KD cells with overexpression of NAT10 wild-type (WT), overexpression of NAT10-G641E mutant and control PDAC cells. **(K-L)** Representative immunofluorescent staining (left) and mean fluorescence intensity (MFI) (right) of phosphor-Src (K) and phosphor-FAK (L) in PANC-1 cells. Scale bar: 10 μm. **p*<0.05, ***p*<0.01, ****p*<0.001. (K-L), One-way ANOVA.

**
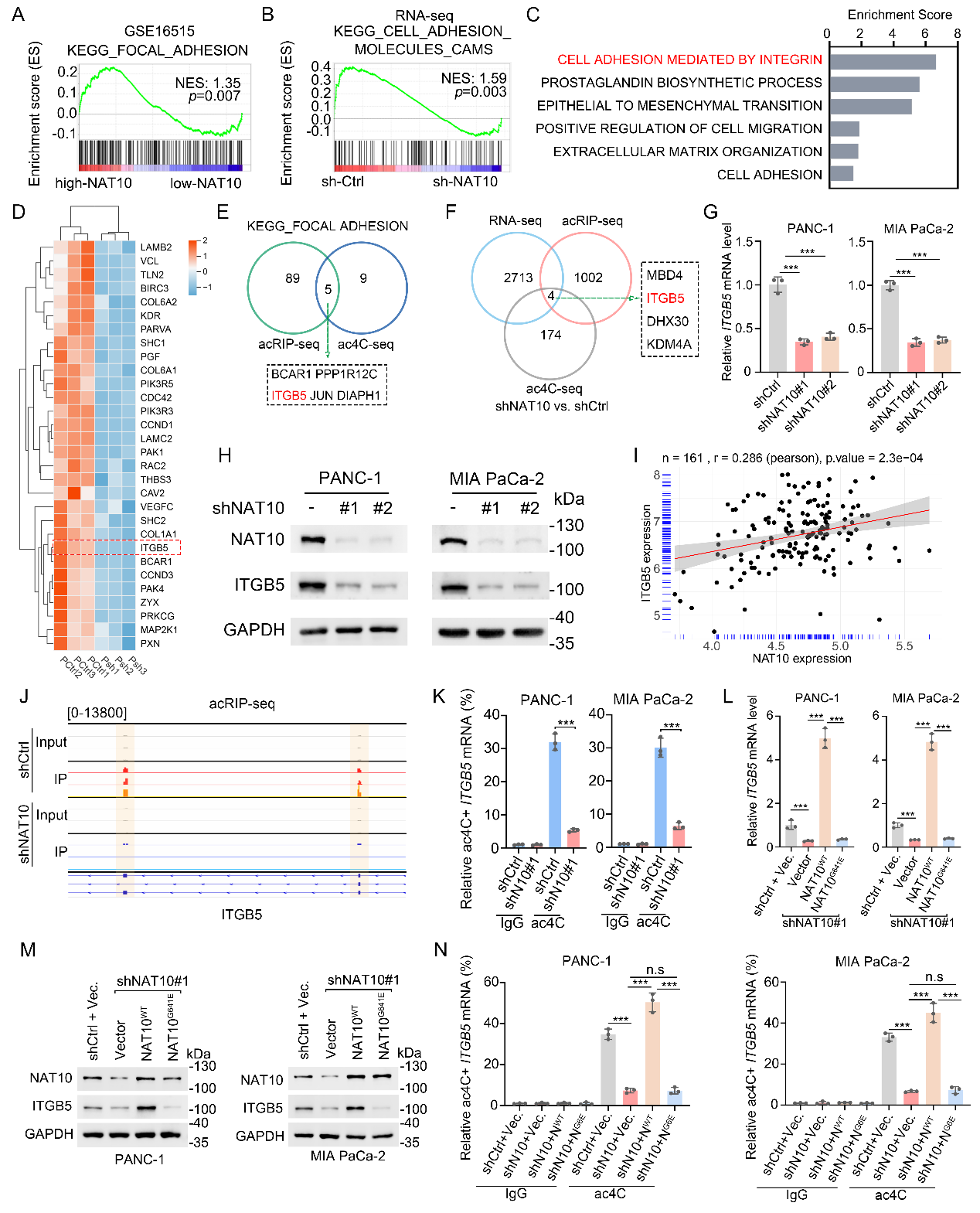
**

**Fig. 4. *ITGB5* is the downstream target of ac4C modification in PDAC cells. (A-B)** Gene-set enrichment analysis (GSEA) from GSE16515 (A) and our RNA-seq data (B) revealed that the focal adhesion pathway and related cell adhesion molecules were enriched in high NAT10 group. **(C)** Gene ontology (GO) analysis of our RNA-seq data showing enrichment of NAT10 KD cells, indicating that downregulated genes are related to cell adhesion mediated by integrin. **(D)** Heap map of focal adhesion pathways genes from RNA-seq analysis. Z-score was used. **(E)** Venn diagram of overlapping enriched genes of the focal adhesion pathway (KEGG) from acRIP-seq and ac4C-seq data. **(E)** Venn diagram of overlapping genes from acRIP-seq, RNA-seq, and ac4C-seq showing the downregulated genes in NAT10 knockdown PANC-1 cells. **(F-H)** Relative NAT10 and ITGB5 mRNA (F-G) and protein (H) expression in NAT10 KD and control PDAC cells. n=3. (I) CPTAC database showing the correlation between NAT10 and ITGB5 mRNA levels. **(J)** Integrative Genomics Viewer (IGV) browser showing the ac4C peaks in *ITGB5* CDS region. **(K)** ac4C RNA-immunoprecipitation (acRIP) followed by qRT-PCR analysis showing the downregulated ac4C level of *ITGB5* in NAT10 KD cells. **(L-M)** Relative ITGB5 mRNA (K) and NAT10 and ITGB5 protein (L) expression in NAT10 KD PDAC cells with overexpression of the NAT10-WT and NAT10-G641E mutant. **(N)** acRIP-PCR analysis indicating the ac4C positive level of ITGB5 mRNA in NAT10 KD treated with overexpression of NAT10 wild-type (WT), overexpression of NAT10-G641E mutant, and control PANC-1 (left) and MIA PaCa-2 (right) cells. **p*<0.05, ***p*<0.01, ****p*<0.001. (G), (L), and (N), One-way ANOVA. (K), Student’s test.

**
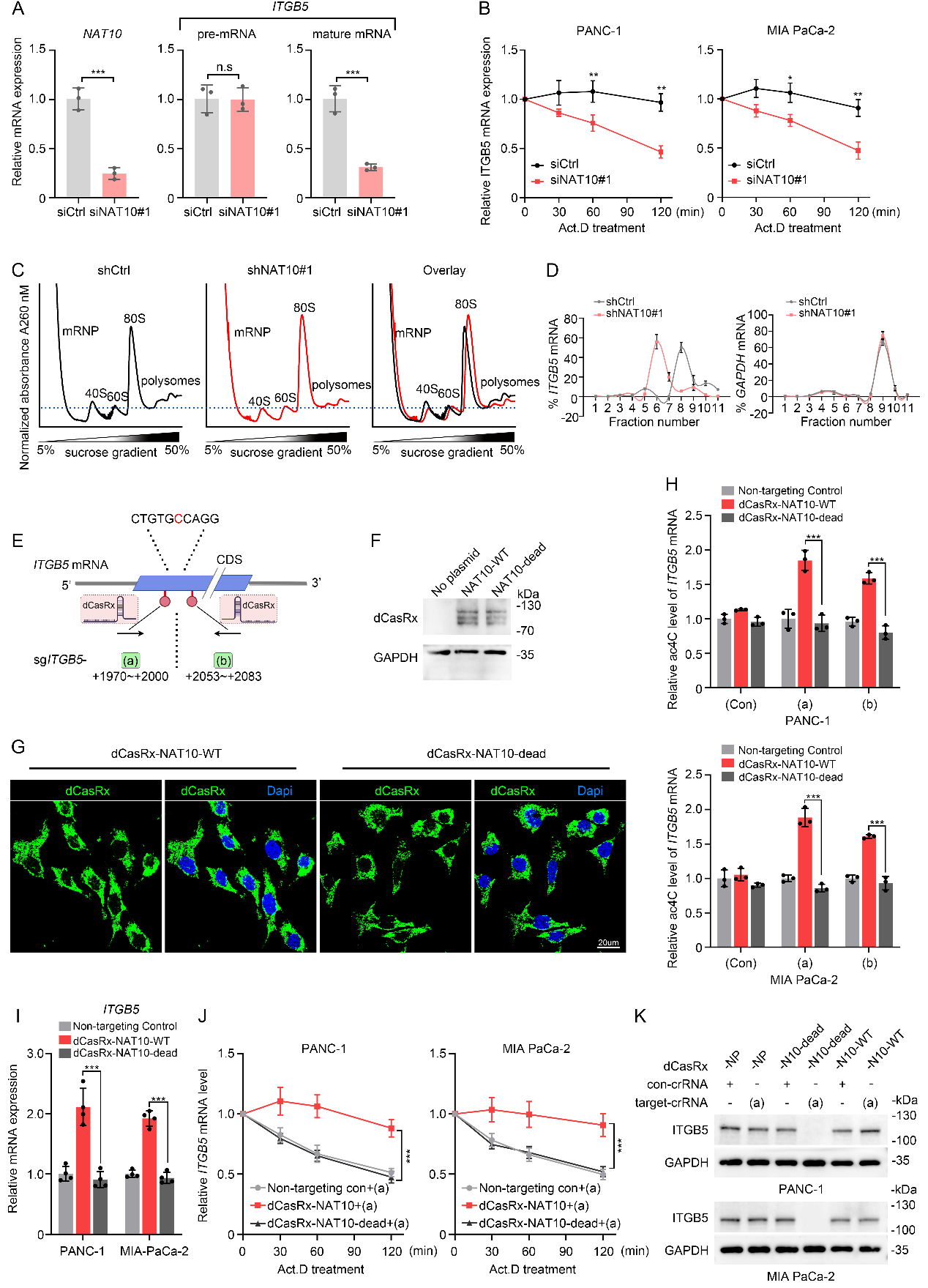
**

**Fig. 5. Knockdown of ac4C modification mediated by NAT10 expedites the *ITGB5* mRNA degradation. (A)** qRT-PCR analysis showing the specified mRNA level in PANC-1 cells expressing NAT10 siRNA (siNAT10) or control siRNA (siCtrl) for 72 h. **(B)** The half-life of ITGB5 mRNA in PANC-1 and MIA PaCa-2 cells transfected with siCtrl or siNAT10. PDAC cells were treated with actinomycin D (Act.D, 5μg/mL) and collected at the indicated time for qRT-PCR analysis. **(C)** PANC-1 cells were transfected with indicated shRNAs. Polysome profiling was performed using 5-50% sucrose gradient fractionation and the absorption of the gradient at 260 nm was recorded. Representative images are shown. **(D)** Quantification of ITGB5 mRNA in 11 gradient fractions using qRT-PCR, from three replicates of polysome profiling. **(E)** Schematic illustration of the CRISPR-dCasRx method to engineer ac4C writer NAT10. Effective small guide RNA (sgRNA) targeting the CDS in the ITGB5 mRNA. sgRNA (a) and (b) were targeting the potential ac4C positive cytosine (red). **(F)** NAT10-KD PANC-1 cells were transfected with CRISPR/dCasRx protein fused with NAT10 or acetylase-dead NAT10 G641E (NAT10-dead). Immunoblotting showing the dCasRx fusion protein. **(G)** Subcellular localization of dCasRx-NAT10 and dCasRx-NAT10-dead fusion protein in NAT10-KD PANC-1 cells. Scale bar: 20μm. **(H)** acRIP analysis followed by qRT-PCR, showing the ac4C levels on endogenous *ITGB5* mRNA in NAT10-KD PANC-1 (upper) and MIA PaCa-2 (lower) cells, which were transfected with indicated dCasRx fusion proteins and sgRNA (a), sgRNA (b), or control (con) sgRNA. **(I)** Relative ITGB5 mRNA expression in NAT10-KD PDAC cells transfected with indicated dCasRx fusion proteins and sgRNA (a). **(J)** *ITGB5* mRNA decay in NAT10-KD PDAC cells transfected with indicated dCasRx fusion proteins and sgRNA (a). **(K)** Immunoblotting images showing endogenous ITGB5 protein expression when PDAC cells were treated with indicated dCasRx proteins and sgRNAs for 48 h. **p*<0.05, ***p*<0.01, ****p*<0.001. (A), Student’s t-test. (B) and (I), One-way ANOVA. (H) and (J), Two-way ANOVA.

**
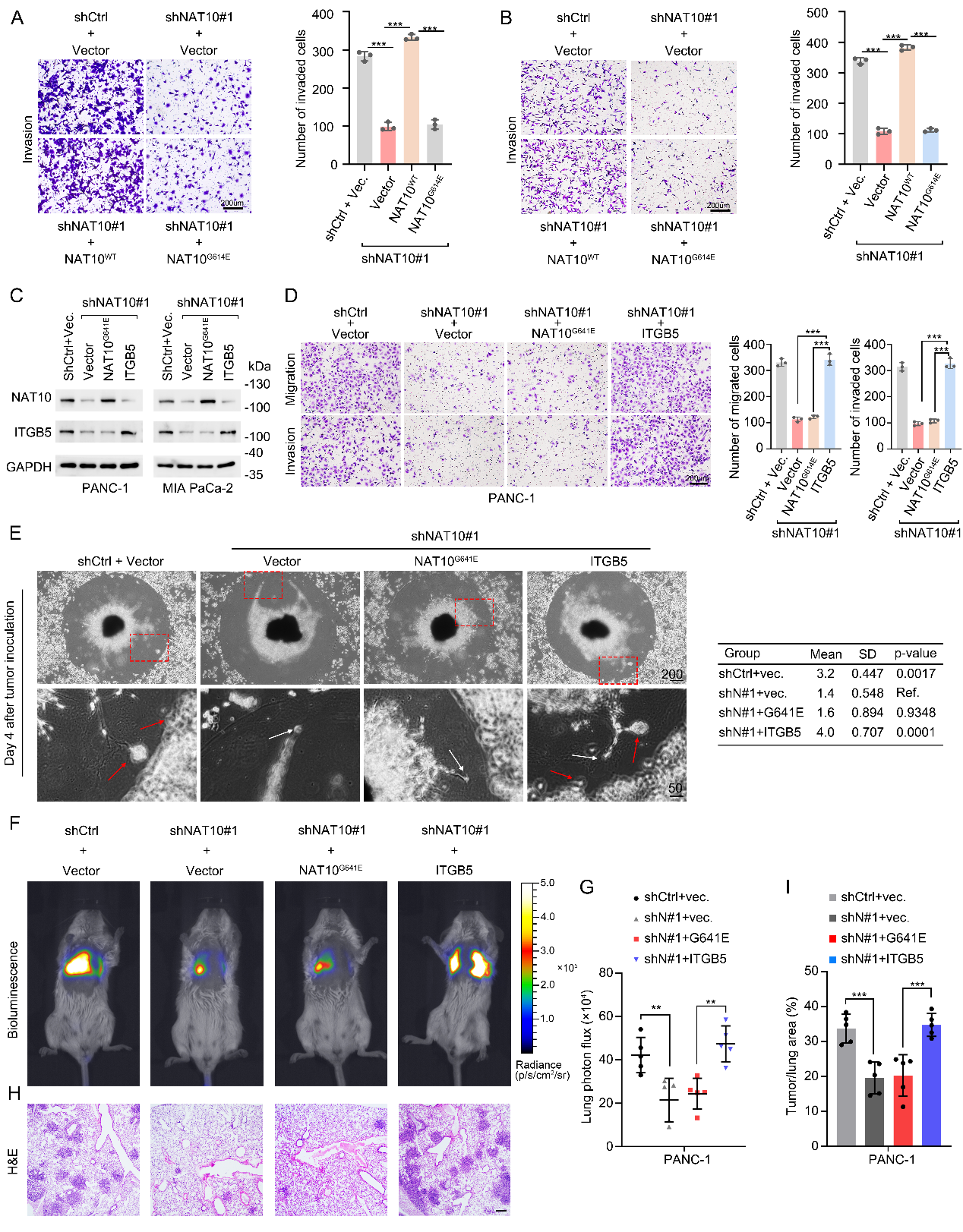
**

**Fig. 6. NAT10-mediated ac4C modification promotes migration, invasion, and metastasis via ITGB5. (A-B)** Representative image and quantification of Transwell invasion in the NAT10 ^G641E^ mutant of NAT10-KD PANC-1 (A) or MIA PaCa-2 (B) cells. Scale bar: 200 μm. **(C)** Representative immunoblotting image of NAT10 and ITGB5 in PANC-1 (left) and MIA PaCa-2 cells (right). **(D)** Representative image and quantification of Transwell assay results for ITGB5-overexpressing NAT10-KD PANC-1 cells. Scale bar: 200 μm. **(E)** Representative image (left) and quantification (right) of DRG model in NAT10 G641E mutant and ITGB5-overexpressing PANC-1 cells. Upper scale bar: 200 μm; lower scale bar: 50 μm. **(F-G)** Representative images (F) and quantification (G) of lung metastasis detected via bioluminescence imaging. **(H-I)** Representative H&E images (H) and the area of the tumor (I) of indicated groups. Scale bar: 200 μm. **p*<0.05, ***p*<0.01, ****p*<0.001. (A-B), (D-E), (G), and (I), One-way ANOVA.

**
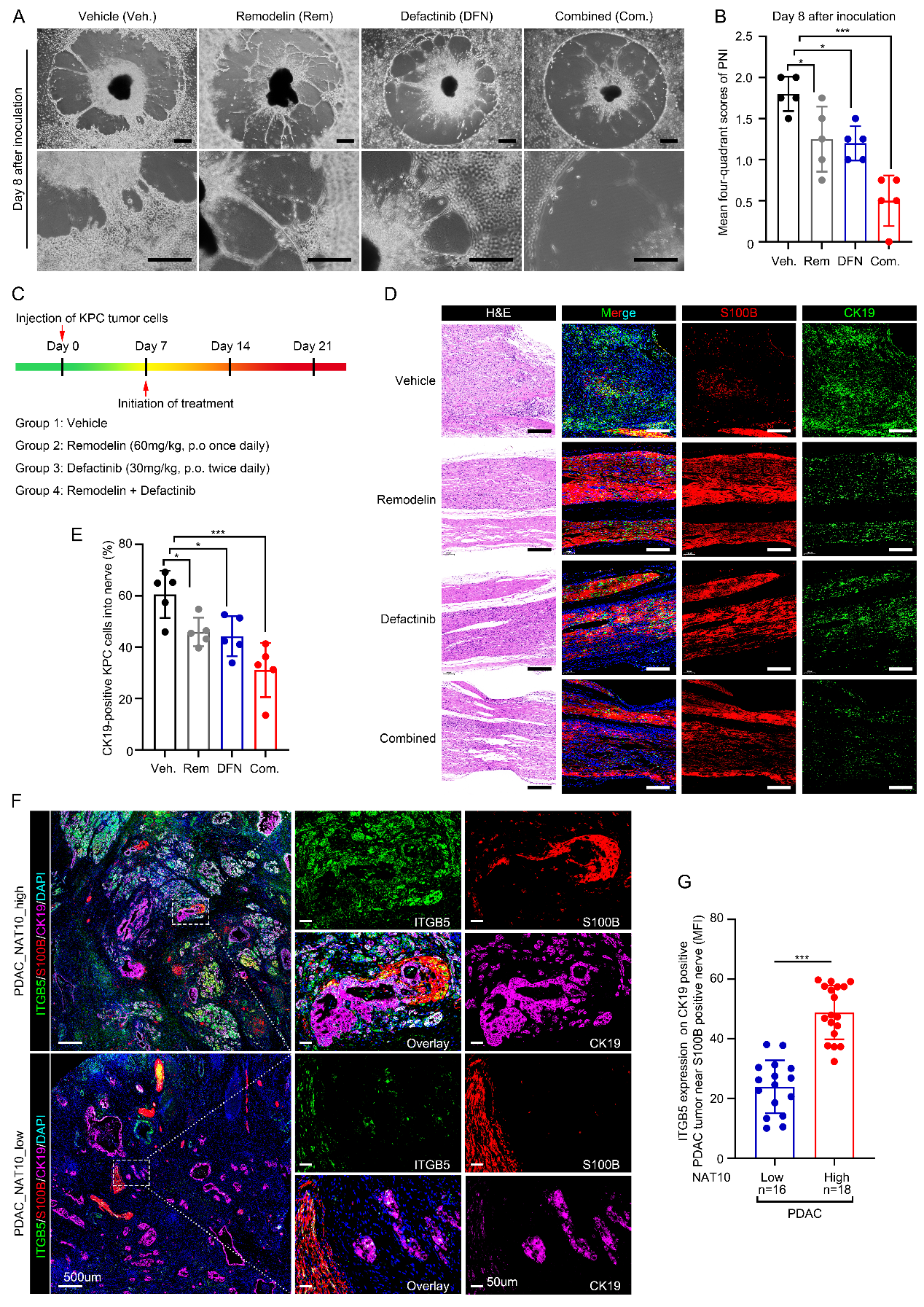
**

**Fig. 7 Clinical relevance of the NAT10-ITGB5-focal adhesion axis in the PNI of PDAC. (A-B)** Representative image (A) and quantification (B) of the DRG model treated with the small-molecule inhibitor Remodelin (Rem), FAK inhibitor defactinib (DFN), and a combination of Rem and DFN. Scale bar: 200 μm. n=5. **(C)** Schematic illustration of KPC tumor cells in the sciatic nerve of C57/Bl6 mice. **(D)** Representative immunofluorescent image of PNI in the sciatic nerve from the above groups. S100B, Schwann cells; CK19, PDAC cells. Scale bar: 200 μm. **(E)** Percentage of CK19-positive PDAC cells in the sciatic nerve from (D). n=5. **(F-G)** Representative mIHC images (F) and statistical analysis (G) of ITGB5 expressed in CK19-positive cells of PDAC tissues from patients with NAT10-high or NAT10-low PDAC (revealed by IHC). Scale bar (left): 500 μm. Scale bar (middle and right): 50 μm. **p*<0.05, ***p*<0.01, ****p*<0.001. (B), and (E) One-way ANOVA. (G), unpaired Student’s test.
