# Supplementary material for: N4-acetylcytidine modification of ITGB5 mRNA mediated by NAT10 promotes perineural invasion in pancreatic ductal adenocarcinoma
Source: J Exp Clin Cancer Res. 2025 Mar 22;44:103. doi: 10.1186/s13046-025-03362-2 (PMC11929334; doi:10.1186/s13046-025-03362-2)
Supplement: Supplementary file 3 — Supplementary Material 3 [file 13046_2025_3362_MOESM3_ESM.pdf]

POINT-BY-POINT RESPONSE TO REVIEWERS' COMMENTS

Reviewer #1 (Comments to the Author)

This is an interesting article where the authors report the role of Nat10 in N4-acetylcytidine modification of ITGB5 mRNA in pancreatic cancer. The authors claim that perineural invasion of the pancreatic ductal adenocarcinoma (PDAC) is mediated by Nat10- N4-acetylcytidine of ITGB5 in vitro and in vivo and in clinical tumor samples. The experimental design is sound, and the data supports the conclusion. However the major concern for publishing the work in JECCR is lack of novelty.

The role of Nat10 in pancreatic cancer is reported previously, where the study found a different oncogene (AXL) mRNA is stabilized by Nat10-mediated ac4C modification (PMID: 37156457). Apparently, Nat10 is not specific for a given mRNA or at least, it has several other target mRNAs. There are several studies that report the role of ac4C acetylation that regulates mRNA stability (PMID: 37484432, PMID: 38531654, PMID: 39030964) and play certain roles in cancer in general. That way, the study lacks novelty. Why the authors have focused on ITGB5 mRNA is not clear.

Response: Thank you very much for finding "The experimental design is sound, and the data supports the conclusion". We greatly appreciate the opportunity to address your concerns and provide clarification regarding the novelty of our study and the focus on *ITGB5* mRNA. Below, we outline our responses and highlight the unique contributions of our work.

The role of Nat10 in pancreatic cancer is reported previously, where the study found a different oncogene (AXL) mRNA is stabilized by Nat10-mediated ac4C modification (PMID: 37156457).

Response: We thank you for the thoughtful comments. We have cited this study in our previous manuscript **(Ref. 23, Line 99, 463, and 482**). While we agree that Nat10 has been previously reported in pancreatic cancer, we believe our study offers significant novelty for the following reasons:

1. Clinical relevance and focus on perineural invasion (PNI):

Perineural invasion (PNI) represents a critical clinical challenge in pancreatic cancer. It is a distinct pathological feature of pancreatic cancer that is closely linked to poor prognosis, significantly contributes to the low resection rate, and, as noted by Reviewer #2, may be a key factor in local recurrence after resection. Recent advances in cancer neuroscience (*Cell*, 2023; *Nature*, 2023; *Cancer Cell*, 2023) [1-3] also highlight the critical role of tumor-nerve crosstalk in tumor progression, yet the specific mechanisms underlying PNI remain poorly understood.

While Nat10 may regulate various genes and pathways in pancreatic cancer progression, our study focuses specifically on its role in regulating PNI, which, to our knowledge, has not been reported before even in cancer. Here, we identified ITGB5 as a key downstream of in this context, providing a novel and specific mechanistic insight into PNI and highlighting its potential as a therapeutic target in pancreatic cancer.

2. Identify RNA modification in PNI patients:

Rather than directly focusing on Nat10 or ac4C modifications, we began with an unbiased investigation of RNA modification changes in PDAC patients with and without PNI across two independent cohorts. Using UPLC/MS-MS, we comprehensively analyzed 55 types of RNA modifications and identified a significant upregulation of ac4C modification in the PNI group in both cohorts **(Fig. 1A-C)**. This finding prompted us to further explore its role and underlying mechanisms, ultimately identifying Nat10 as a key regulator in this process.

This methodological approach, which begins with clinical specimens and progresses to mechanistic studies, set out our study from previous study in PDAC (PMID: 37156457) that primarily focused on Nat10's broader roles in pancreatic cancer. By linking ac4C modification and NAT10 specifically to PNI, our study provides a novel perspective on Nat10's functions, and emphasize its translational relevance in the context of PNI in PDAC.

3.Technical Advancements and Reliability:

To identify the significant RNA modification associated with PNI, as mentioned above, we first employed **UPLC/MS-MS** to comprehensively explore reported RNA modifications in PDAC patients with or without PNI **(Fig. 1A-C)**. Then, to further investigate the regulatory mechanism of NAT10-mediated ac4C modification, we employed the well-reported technique on ac4C modification based on RNA immunoprecipitation (RIP), i.e. **acRIP-seq** in NAT10 knockdown PANC-1 cells **(Fig. 3B-C)**.

Considering the susceptibility of false positives with conventional acRIP-seq and its inability to detect ac4C at nucleotide resolution (*Nat Protoc*, 2021; *Nature, 2020*) [4,5] [4,5]. we combined it with **single-nucleotide resolution ac4C-seq** in NAT10-KD PANC-1 cells. This allows us to find the potential target of NAT10-modified mRNAs.

By building on these recent advancements in ac4C modification detection, our approach represents a methodological improvement over earlier studies. Leveraging these advanced techniques, we were able to reliably identify ITGB5 as a downstream target of Nat10 in PNI. This robust and innovative strategy not only strengthens the validity of our findings but also underscores the technical rigor of our study, further supporting its significance in understanding PNI mechanisms in PDAC.

**Aspect**	**Previous report (PMID: 37156457, *Exp Cell Res*. 2023)**	**Our study**
**Clinical question**	Focusing on NAT10 in PDAC for intervention, and broader function of cancer.	Focusing on PNI, a unique pathological characteristic, which strongly related to poor prognosis in PDAC.

**RNA modification**	Directly evaluated NAT10 levels in PDAC to determine its role in pancreatic cancer.	Began with an unbiased exploration of RNA modifications alteration in PDAC with PNI.<br>We found that RNA modifications, especially ac4C levels were upregulated, then to explore NAT10
**ac4C methods**	1. RNA-seq to identify *AXL* as a downstream<br>2. acRIP-qPCR to validate	1. UPLC/MS-MS to analyze RNA modification in PDAC tissues with and without PNI;<br>2. acRIP-seq to identify focal adhesion pathway;<br>3. ac4C-seq to further validate ITGB5 as the downstream when combined the results with acRIP-seq and RNA-seq;<br>4. acRIP-qPCR to validate

Apparently, Nat10 is not specific for a given mRNA or at least, it has several other target mRNAs. There are several studies that report the role of ac4C acetylation that regulates mRNA stability (PMID: 37484432, PMID: 38531654, PMID: 39030964) and play certain roles in cancer in general. That way, the study lacks novelty. Why the authors have focused on ITGB5 mRNA is not clear.

Response: Thank you for your constructive suggestion. We agree that Nat10 is not specific to a single mRNA and likely regulates multiple target transcripts. In fact, this is an important consideration that we had carefully addressed during the initial design of our study, and it has been discussed in the manuscript.

As previously described in the manuscript, we first utilized acRIP-seq and ac4C-seq to identify ac4C-modified transcripts enriched in the focal adhesion pathway. This analysis revealed five candidate genes **(Fig. 4E)**. We further validated these findings using acRIP-qPCR, which confirmed that the ac4C levels of three transcripts **(*ITGB5*, *JUN*, and *BCAR1*)** were significantly reduced in NAT10-KD cells **(Line 288, Fig. S4B)**. The details are provided below for your reference:

Line 284: "To identify specific targets within the focal adhesion pathway regulated by ac4C modification, we overlapped acRIP-seq and ac4C-seq data, focusing on ac4C-positive genes from the focal adhesion pathway based on KEGG enrichment results **(Fig. 3G-H)**. This analysis highlighted *BCAR1*, *JUN*, *ITGB5*, *PPP1R12C*, and *DIAPH1* as potential candidates **(Fig. 4E)**. acRIP assays confirmed decreased mRNA ac4C enrichment for *ITGB5*, *JUN* and *BCAR1* in NAT10-KD cells **(Fig. S4B)**."

Additionally, as you mentioned, the general mechanism of RNA ac4C acetylation in tumors involves regulating mRNA stability, which has also been reported in

pancreatic cancer. Given that, we further integrated RNA-seq data to identify downstream genes within the focal adhesion pathway whose mRNA stability might be regulated by ac4C modification. This analysis revealed four can*didate genes (MBD4, ITGB5, DHX30, and KDM4A), among which ITGB5 exh*ibited the highest ac4C peak level.

Line 290: "To further narrow down our transcriptional targets, we combined these sequencing data with our RNA-seq data, specifically examining downregulated transcripts associated with hypoacetylation (FDR<0.05, log2 (FC) <-1), which revealed a set of genes, including *MBD4*, *ITGB5*, *DHX30*, and *KDM4A* **(Fig. 4F)**, with *ITGB5* exhibiting the highest ac4C peak **(Fig. S4C-F)** among these transcripts."

Considering the three aspects you mentioned, our sequencing results and multiple experimental validations consistently demonstrated that ac4C modification regulates the focal adhesion pathway. Among the identified targets, ITGB5 was confirmed as a critical downstream of NAT10, mediating the activation of FAK-Src phosphorylation.

In summary, sincerely appreciate your insightful comments, which align closely with the considerations we had incorporated into our study design. We respectfully submit that our study offers novelty and significance from multiple perspectives. We sincerely hope this response sufficiently addresses your concerns Thank you again for your valuable feedback, which has helped us further clarify and strengthen our study..

Reviewer #2 (Comments to the Author)

This is a very interesting work that demonstrated N4-acetylcytidine modification of ITGB5 mRNA mediated by NAT10 promotes perineural invasion in pancreatic ductal adenocarcinoma. The perineural invasion of pancreatic cancer is one of its important biological behaviors, which plays a significant role in the low resection rate of pancreatic cancer and may be a key factor in local recurrence after resection. Intervention targeting its perineural invasion may have an important role in improving the resection rate and prognosis of pancreatic cancer.

Response: We sincerely appreciate your positive feedback and for describing our work "very interesting". Thank you for highlighting the critical role of perineural invasion in pancreatic cancer. We fully agree that targeting perineural invasion represents a promising strategy, and we hope our findings contribute to advancing this important area of research.

Here are some minor suggestions.

1. Can the authors analyze the difference in NAT10 expression between patients with and without local neurovascular invasion in the primary lesion of stage IV pancreatic cancer?

Response: Thank you for this important suggestion. Following your request, we analyzed NAT10 expression in the primary lesions of stage IV pancreatic cancer. Clinically, local vascular invasion is primarily assessed using imaging techniques such as CT scans, which are commonly used to evaluate the tumor resectability and

potential vascular reconstruction. However, accurately assessing local perineural invasion through imaging before surgery remains a significant challenge.

In our cohorts, we identified 8 cases with local vascular invasion. Among these, 6 cases showed high NAT10 expression, as below **(Revised Table. 1-2)**. Due to the limited sample size, statistical analysis would not yield meaningful results at this stage. Additionally, patients with vascular invasion outside the tumor generally lose the opportunity for surgical resection. We acknowledge the need and we plan to expand our sample size in future studies to further evaluate this important relationship. Thank you again for your valuable suggestion, which will help guide the direction of our future research.

Revised Table. 1 Correlation of NAT10 expression with local vascular invasion in PDAC patients from cohort 1.

Characteristics	Number of cases	NAT10 expression	
		Low (n=30)	High (n=32)
Local vascular invasion			
No	60	29	31
Yes	2	1	1

Revised Table. 2 Correlation of NAT10 expression with local vascular invasion in PDAC patients from cohort 2.

Characteristics	Number of cases	NAT10 expression	
		Low (n=109)	High (n=123)
Local vascular invasion			
No	226	108	118
Yes	6	1	5

2. Can the authors provide information on neural invasion and local recurrence in patients with resectable pancreatic cancer who have different levels of NAT10 expression?

Response: Following your request, we analyzed the relationship between NAT10 expression levels and neural invasion as well as local recurrence in patients with resectable pancreatic cancer. Below, we provide the relevant data and observations for your reference **(Revised Table. 3)**:

Revised Table. 3 Correlation of NAT10 expression with perineural invasion or local recurrence in PDAC patients from cohort 2.

Characteristics	Number of cases	NAT10 expression		*p* value
		Low (n=109)	High (n=123)	
Perineural invasion				
No	91	52(22.4%)	39(16.8%)	**0.013***
Yes	141	57(24.6%)	84(36.2%)	
Local recurrence				
No	83	39(16.8%)	44(19.0%)	0.999
Yes	149	70(30.2%)	79(34.1%)	

*p<0.05.

Reviewer #3 (Comments to the Author)

This manuscript by Huang et al. presents an intriguing study. The authors demonstrate the role of RNA modification in PDAC patients with PNI. They demonstrate that NAT10 expression in tumor cells promotes PNI via the ac4C-ITGB5-pFAK-pSrc axis, highlighting the crucial role of ac4C modification in PNI, providing a therapeutic target for preventing PNI and improving the prognosis of patients with PDAC. These conclusions are well-supported by reliable in vitro and in vivo experimental validations. However, there are still several minor concerns that need to be addressed.

Response: We sincerely appreciate you for finding our study "intriguing" and "conclusions are well-supported". Thank you for taking time to evaluate our manuscript and for your positive appraisal of our work.

1. Annotated legend, figure 1D, PNI group, figure 5I, no annotated legend, please add. The position marked in supplementary figure 1B is inappropriate, please adjust it. Figure 1 legend, repeated 1I but not 1H, please revise.

Response: Thank you for pointing out these issues. Following your suggestion, we have carefully revised the figures and corrected them in the revised manuscript as follows. We have updated the legend for Figure 1D. The revised figure and legend are provided below for your reference **(Fig. 1D** and **Revised Fig. 1)**.

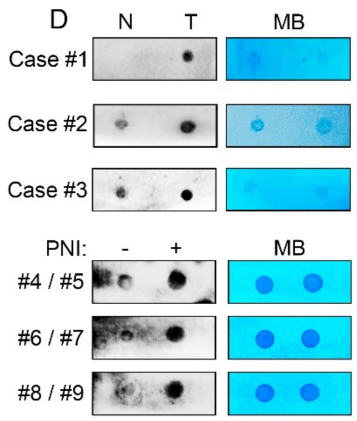

Revised Fig. 1 Related to Fig. 1D. (D) RNA dot blot assays indicating a consistent increase in ac4C levels in three paired PDAC tissues (T) when compared to levels in paired normal (N) pancreas (upper panel). The lower panel compares between PDAC tissues with PNI and those without PNI. MB: Methylene Blue.

We have updated the annotated legend for Figure 5I. The revised figure is provided below for your reference **(Fig. 5I** and **Revised Fig. 2)**.

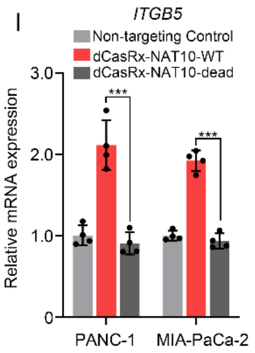

Revised Fig. 2 Relate to Fig. 5. (I) Relative ITGB5 mRNA expression in NAT10-KD PDAC cells transfected with indicated dCasRx fusion proteins and sgRNA (a). ****p*<0.001.

We have adjusted the position to more accurately reflect the intended region of Pancreatic adenocarcinoma (PAAD) **(Fig. S1B** and **Revised Fig. 3)**. The marking is now appropriate and clearly highlights the relevant area as follows:

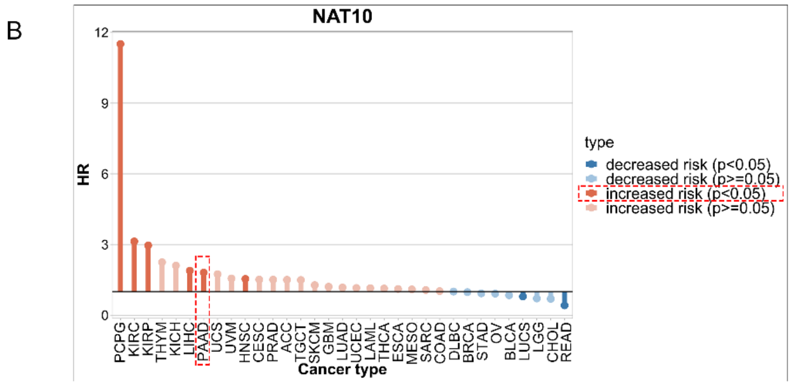

Revised Fig. 3 Related to Fig. S1B. (B) Analysis using TISCH showed the NAT10's hazard ratio (HR) and p-value across 33 cancer types in TCGA.

We thank you for finding the repeated to "Figure 1I" in the Figure 1 legend, and it has now been corrected to "Figure 1H". The legend now accurately corresponds to the figures presented **(Line 750)**. We apologize for the typos and appreciate your careful review. All corrections have been updated in the revised manuscript.

2. As shown in Figure 1I, the expression level of NAT10 in PNI patients was divided into two parts in WB. Are they different cohorts? Or some other reason?

Response: Thank you for your comments and suggestion. The data presented in Figure 1I are from two independent cohorts of patients. The first two pairs of samples are from cohort 1 (Sun Yat-sen Memorial Hospital), which was part of the initial patient cohort included in our study. The third pair of samples is from cohort 2 (Guangdong Provincial People's Hospital), which was subsequently included during the course of the study to expand the sample size and further validate our findings.

To ensure consistency, we used the reagents from the same source and the same

antibody concentrations for both cohorts. GAPDH was used as the control for normalization of NAT10 expression. Additionally, the original Western blot images are provided in the Supplementary Materials for transparency. We appreciate your careful review and hope this addresses your concerns.

3. In Figure 2, the authors knockdown NAT10 in cells with high expression of NAT10, proving that NAT10 KD inhibited PNI of tumor cells. Are tumor cells with low NAT10 expression less invasive than high NAT10 expression cell lines?

Response: Thank you for your thoughtful question. As shown in our observations **(Fig. 2A)**, compared to the pancreatic ductal epithelial hTERT-HPNE cell line, PANC-1, MIAPaCa-2, and SW1990 exhibit higher NAT10 expression levels, while CFPAC-1 shows relatively lower NAT10 expression.

According to the ATCC database, the CFPAC-1 line was derived from liver metastases of a cystic fibrosis pancreatic adenocarcinoma, and the SW1990 line was established from spleen metastasis of pancreatic adenocarcinoma (https://www.atcc.org/products, crl-1918 and crl-2172), indicating their inherent metastatic potential. However, compared to other commonly used pancreatic cancer cell lines, these two cell lines do not always exhibit the strongest invasive abilities *in vitro* and *in vivo*.

The process of invasion, involves tumor cells acquiring the ability to penetrate surrounding tissues, including the basement membrane and extracellular matrix, which is a critical step preceding intravasation and subsequent metastasis (*Nat Rev Cancer*. 2014; 14: 495-501) [6] . However, the invasive potential of a cell line *in vitro* does not always directly correlate with its metastatic origin or capacity, highlighting the complexity and the limitation to governing cancer invasion and metastasis.

To further investigate the role of NAT10 in invasion, we overexpressed NAT10 in CFPAC-1 cells **(Revised Fig. 3A-B)**, which naturally exhibit low NAT10 expression. After 24h of culture, we found that NAT10 overexpression significantly enhanced the invasive capacity of CFPAC-1 cells *in vitro* **(Revised Fig. 3C-D)**. We hope this addresses your question on the relationship between NAT10 expression and invasive potential in tumor cells.

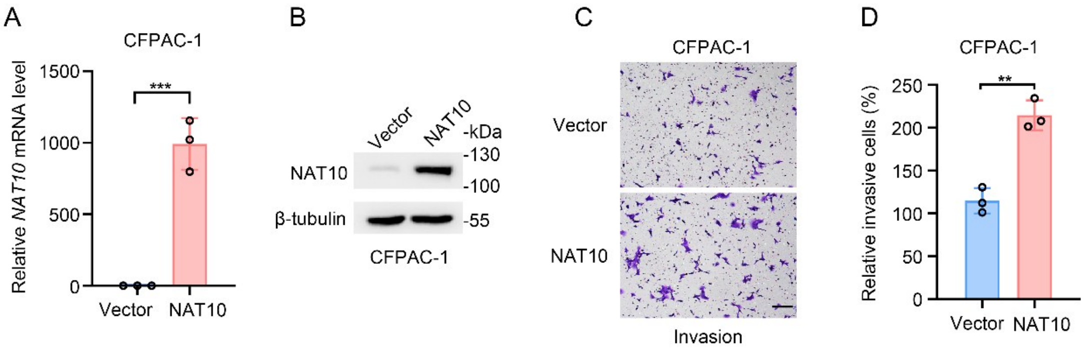

Revised Fig. 3 Overexpression (OE) of NAT10 promotes tumor invasion in CFPAC-1. (A-B) Relative NAT10 mRNA expression (A) and representative immunoblots (B) in NAT10 OE and vector CFPAC-1 cells. **(C-D)** Representative

image (C) and quantification (D) of Transwell migration and invasion in NAT10 OE and vector CFPAC-1 cells. Scale bar: 200 μm. *p<0.05; **p<0.01; ***p<0.001.

4. Figure 3. The authors found that NAT10 mRNA expression was positively correlated with CDC42 and PXN, and NAT10-KD could lead to up-regulation of E-cadherin inhibitor and decrease of MMP9. Then, does NAT10-KD inhibit the expression of CDC42 and PXN? Are NAT10 mRNA levels correlated with E-cadherin and MMP9?

Response: Thank you for your comments. Our results demonstrate that NAT10-KD leads to a significant downregulation of CDC42 protein expression **(Fig. 3I and Revised Fig. 4A)** but does not affect PXN protein levels **(Revised Fig. 4B)**. These findings support our conclusion that NAT10 promotes pancreatic cancer invasion and PNI through *ITGB5* mRNA ac4C modification, which enhances ITGB5 expression and activates the FAK-Src signaling pathway. This is consistent with the established role of CDC42 as a downstream effector of integrin signaling (*Nat Rev Mol Cell Biol*, 2005; 6, 56–68) [7] , which is disrupted by NAT10-KD. PXN, on the other hand, is primarily regulated by its phosphorylation state (Tyr31 and Tyr118) (*Nat Cell Bio*, 2004; 6, 154–161) [8] , which is the downstream of FAK-Src signaling. Our result show that NAT10-KD inhibits FAK and Src phosphorylation, which may explain why PXN total protein levels remain stable in NAT10-KD cells.

From database analysis, NAT10 mRNA levels are positively correlated with CDC42, PXN, MMP9, and CDH1 mRNA levels **(Revised Fig. 4C-F)**. The positive correlation with MMP9 aligns with our experimental finding that NAT10-KD reduces MMP9 expression, consistent with its role in promoting extracellular matrix degradation and invasion. Interestingly, while NAT10 mRNA levels are positively correlated with CDH1 mRNA levels **(Revised Fig. 4F)**, our experimental results show that E-cadherin protein levels are upregulated following NAT10-KD **(Fig. S3E)**. This discrepancy likely reflects the the complex multi-level regulation of E-cadherin expression, such as the post-transcriptional of mRNA and protein degradation. According to our acRIP-seq and ac4C-seq data **(GSE185960 and GSE275963)**, CDH1 mRNA is not affected by ac4C modification. The exact mechanism requires further investigation. In this study, NAT10 may promotes invasion through ac4C-ITGB5-FAK-Src signaling, which indirectly suppresses E-cadherin protein levels, despite the positive correlation between NAT10 and CDH1 mRNA levels in the database. We hope this explanation addresses your concerns.

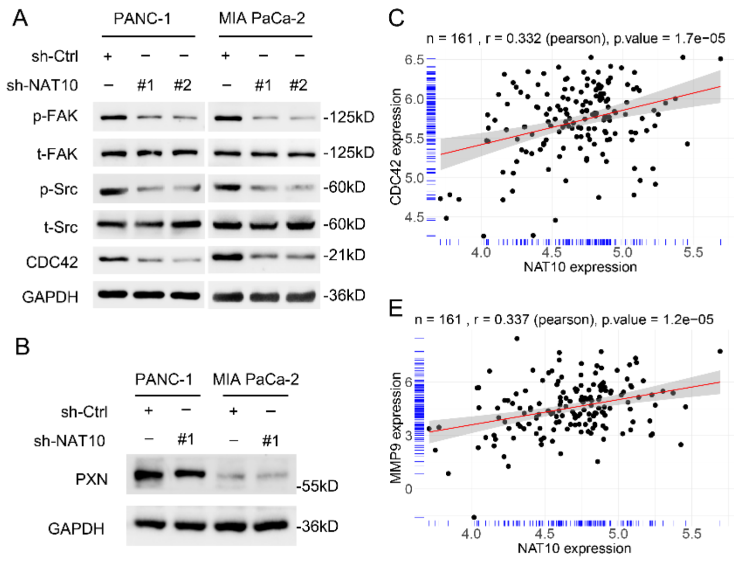

Revised Fig. 4 The protein expression and database analysis between NAT10 and the FAK-Src signaling pathway. (A-B) Immunoblot images of FAK, Src, phospho-FAK, phospho-Src, CDC42 (A) and PXN (B), in NAT10 KD and control PDAC cells. **(C-F)** Correlation between NAT10 expression and CDC42 (C), PXN (D), MMP9 (E), or CDH1 (F) from the Clinical Proteomic Tumor Analysis Consortium (CPTAC) database.

5. Supplementary figure 4B, described by the author as "acRIP assays confirmed decreased mRNA ac4C enrichment for ITGB5, JUN and BCAR1 in NAT10-KD cells". The picture shows changes in four genes but does not show ITGB5.

Response: Thank you for your comment. We apologize for any confusion in the figure description. To clarify, the acRIP assay results for ITGB5 were previously presented in Fig. 4E, and we have now included these data in Supplementary Figure 4B for completeness and consistency. The revised figure is provided below for your reference **(Fig. 4E and Revised Fig. 5)**.

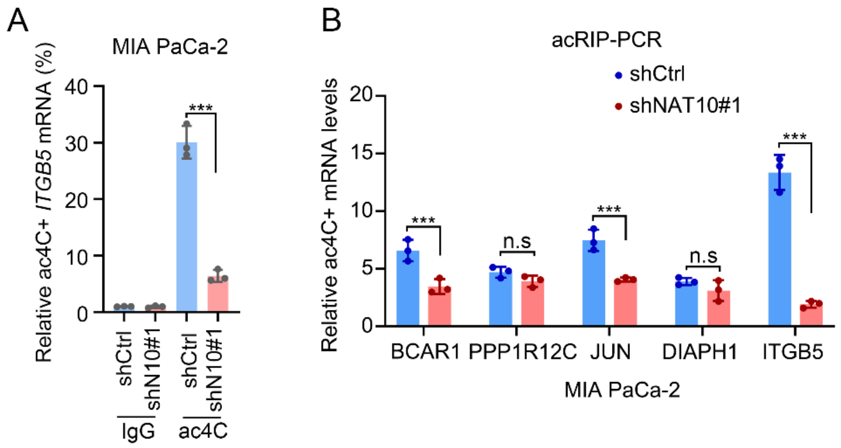

Revised Fig. 5 Related to Fig. 4 and Fig. S4 (A) The unchanged image Fig. 4E showing the relative ac4C+ *ITGB5* level in MIA PaCa-2 cells. ***p<0.001; n.s, not

significance.

6. In Figure 6, ITGB5 promotes the activation of cell adhesion pathways to promote tumor progression. The authors prove that ITGB5 is the key downstream of NAT10. Will the change of NAT10 expression after ITGB5 knockdown affect the invasion ability of tumor cells?

Response: Thank you for your question. To address this, we performed additional experiments examining the effects of individual and combined knockdown of NAT10 and ITGB5. First, we validated the knockdown efficiency **(Revised Fig. 6A-D)**. Our results show that the invasive ability of tumor cells with simultaneous knockdown of NAT10 and ITGB5 (siNAT10+siITGB5) is comparable to that observed with knockdown of either siNAT10 or siITGB5 alone **(Revised Fig. 6E-H)**. These findings suggest that the knockdown of ITGB5 slightly affect NAT10 mRNA levels, and ITGB5 is a key downstream effector of NAT10 in promoting tumor cell invasion. This further support our conclusion that NAT10 promotes PDAC invasion and PNI primarily through ITGB5-mediated activation of FAK-Src pathways.

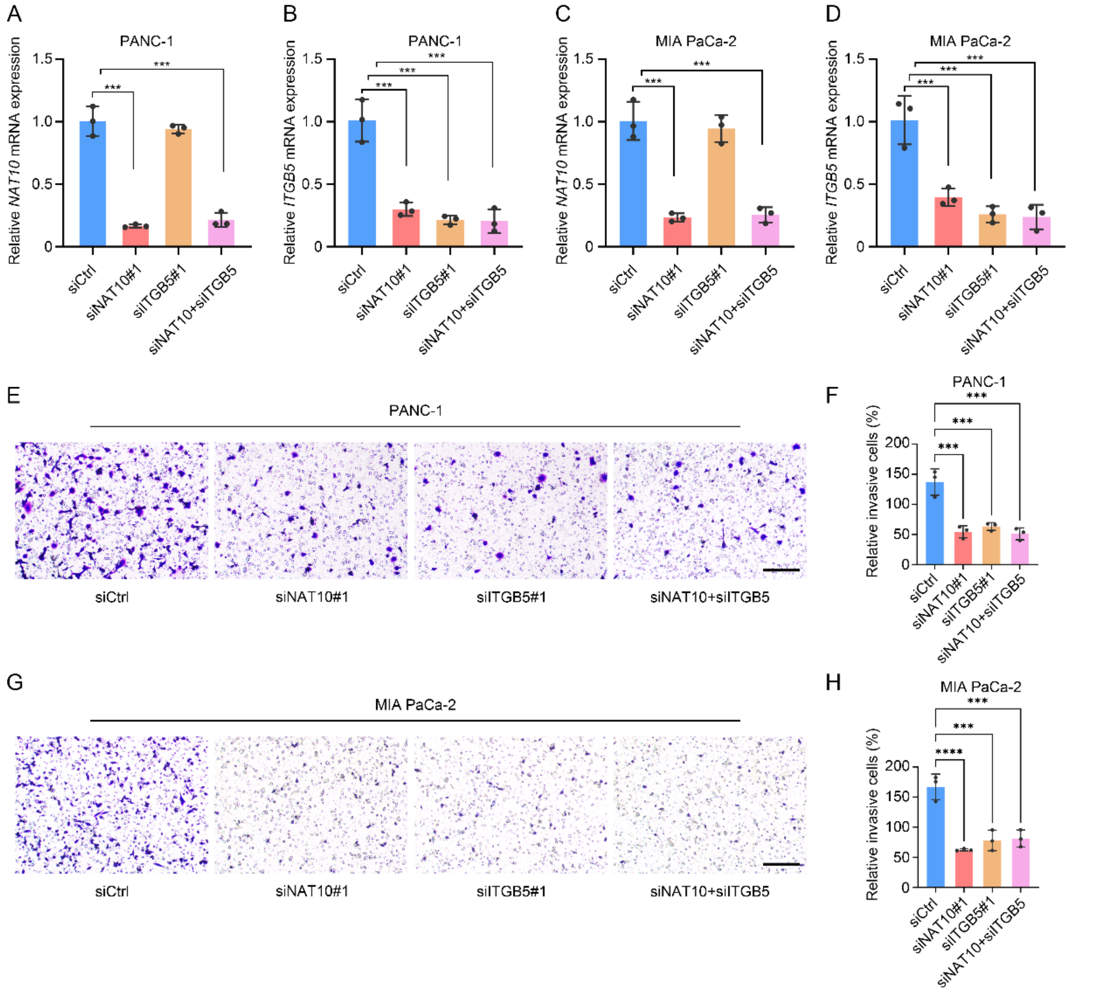

Revised Fig. 6 Combined knockdown of NAT10 and ITGB5 shows no additive effect on pancreatic cancer cell invasion. (A-D) qRT-PCR analysis confirming the knockdown efficiency of NAT10 and ITGB5 in PANC-1 (A-B) and MIA PaCa-2 (C-D)

cells transfected with siControl, siNAT10, siITGB5, or siNAT10+siITGB5. **(E-H)** Representative images and quantification of transwell invasion assays in PANC-1 (E-F) and MIA PaCa-2 (G-H) cells transfected with siControl, siNAT10, siITGB5, or siNAT10+siITGB5. Scale bar, 200 µm. ***p<0.001.

7. The relationship between ITGB5 and Integrin αvβ5 shall be explained.

Response: Thank you for your suggestion. We have explained the relationship in **Line 299**, as followed:

"ITGB5 encodes the β5-subunit of the integrin, a transmembrane receptor that exclusively pairs the β5 subunit with the αv subunit, promotes tumor progression[35] and is associated with poor prognosis in PDAC[36]."

References:

1. Winkler F, Venkatesh HS, Amit M, Batchelor T, Demir IE, Deneen B, et al. Cancer neuroscience: State of the field, emerging directions. Cell. 2023; 186: 1689-707.
2. Mancusi R, Monje M. The neuroscience of cancer. Nature. 2023; 618: 467-79.
3. Hanahan D, Monje M. Cancer hallmarks intersect with neuroscience in the tumor microenvironment. Cancer Cell. 2023; 41: 573-80.
4. Thalalla GS, Sas-Chen A, Schwartz S, Meier JL. Quantitative nucleotide resolution profiling of RNA cytidine acetylation by ac4C-seq. Nat Protoc. 2021; 16: 2286-307.
5. Sas-Chen A, Thomas JM, Matzov D, Taoka M, Nance KD, Nir R, et al. Dynamic RNA acetylation revealed by quantitative cross-evolutionary mapping. Nature. 2020; 583: 638-43.
6. Slattum GM, Rosenblatt J. Tumour cell invasion: an emerging role for basal epithelial cell extrusion. Nat Rev Cancer. 2014; 14: 495-501.
7. Mitra SK, Hanson DA, Schlaepfer DD. Focal adhesion kinase: in command and control of cell motility. Nat Rev Mol Cell Biol. 2005; 6: 56-68.
8. Webb DJ, Donais K, Whitmore LA, Thomas SM, Turner CE, Parsons JT, et al. FAK-Src signalling through paxillin, ERK and MLCK regulates adhesion disassembly. Nat Cell Biol. 2004; 6: 154-61.
